# Zn-Based Coatings and Durability of High-Strength Pearlitic Bridge Wires: A Critical Evidence Appraisal

**DOI:** 10.3390/ma19214692

**Published:** 2026-11-04

**Authors:** Pei Li, Guoyang Li, Feiling Sun, Xiaofeng Ren, Chaolei Zhang, Guilin Wu

**Affiliations:** Institute for Carbon Neutrality, University of Science and Technology Beijing, Beijing 100083, China; lp1277504201@163.com (P.L.); guoyang1877075@163.com (G.L.); 18811345724@163.com (F.S.); 15624090073@163.com (X.R.); zhangchaolei@ustb.edu.cn (C.Z.)

**Keywords:** galvanized wire, critical evidence appraisal, local corrosion, hydrogen, corrosion fatigue, fretting, cable assessment

## Abstract

Zn-based coatings delay corrosion, while local steel condition and loading determine the mechanical response of bridge wires. This critical evidence appraisal uses 61 selected study families to clarify what coating, hydrogen and fracture measurements establish. Tested products differ in when visible red rust or brown products appear. After steel exposure, fatigue tests assess a different response: with a prescribed 100 μm deep cut, mean life at a stress range of 360 MPa was 203,498 cycles in air and 85,538 cycles in 3.5% NaCl spray. Controlled charging and desorption establish hydrogen-sensitive mechanical responses in the tested pearlitic states; selective desorption distinguishes the mechanical relevance of retained populations. Matched pre-corrosion comparisons and contact tests show why continued wetting and local contact conditions matter. These comparisons guide evidence selection for the observed wire state and response being assessed. The synthesis is interpretive; cable assessment also requires spatial exposure, restraint and load-transfer information, with validation matched to the intended mechanical output.

## 1. Introduction

The durability of high-strength pearlitic bridge wires depends on both the Zn-based coating and the load-bearing steel. Zn-based coatings limit attack on the steel; drawing gives the substrate its strength and strongly oriented microstructure. As the coating is consumed or locally damaged, assessment shifts to the local condition of exposed steel and its response to loading. Within a cable, moisture, interwire contact and restraint add spatial and mechanical variation.

This critical evidence appraisal asks what coating, corrosion, hydrogen and mechanical measurements establish and how their results can inform durability assessment. The principal material unit is the finished bridge wire. Evidence from machined pearlitic specimens, model coatings and cable calculations is used for the questions those studies address, with their material states and endpoints identified.

Visible-rust tests compare the tested coating preparations within a reported exposure protocol; fatigue assessment after local steel exposure requires evidence matched to defect geometry and the loading environment. Desorption–recovery tests support hydrogen involvement, whereas protective-trapping claims require mechanical controls beyond retention observed in separately prepared APT specimens. Validation of wire strength, life or break cycles supports those specific outputs; assessing retained axial force requires assembly-force measurements, and residual ultimate load-bearing capacity requires ultimate-load tests.

## 2. Literature Selection and Evidence Appraisal

### 2.1. Scope and Assessment Questions

Jie et al. [1] review corrosion-fatigue mechanisms and life assessment; Hu et al. [2] review hydrogen transport, trapping and mechanical response; and Zhao et al. [3] link corrosion, fretting and cable assessment. Local iron-bearing products observed before an average-depth criterion is reached and a contact crack with zinc elsewhere in the scar show why average coating endpoints need local steel-damage information. The appraisal distinguishes hydrogen participation supported by desorption–mechanical recovery from retention in separately prepared APT specimens and links each model check to the output actually measured (Supplementary Note S1H).

Field and recovered-wire reports establish the damage encountered in bridges [4,5,6,7]. The principal unit of appraisal is a finished bridge wire; machined pearlitic specimens, related coated materials and models are identified by their material state and endpoint. Quantitative comparisons retain the reported preparation, exposure, loading reference, independent experimental unit and control. Incompatible protocols are not pooled. Table S1 indexes 61 selected families, Table S1E records endpoints and specimen linkage, Tables S2A–S2C retain numerical source records, and Table S3 gives the focused study-by-study appraisal.

### 2.2. Search and Selection

This critical evidence appraisal uses a targeted literature search and source-level comparison. On 12 October 2026, four English bibliographic queries were submitted to the Crossref REST API: ‘galvanized high strength steel wire coating corrosion’; ‘pearlitic steel wire hydrogen embrittlement’; ‘high strength steel wire corrosion pitting fatigue’; and ‘bridge cable strand wire corrosion fatigue load redistribution’. Journal records dated 1 January 2000–12 October 2026 were eligible, without a language filter; the first-100 relevance-ranked records per query were screened.

**Figure 1:**
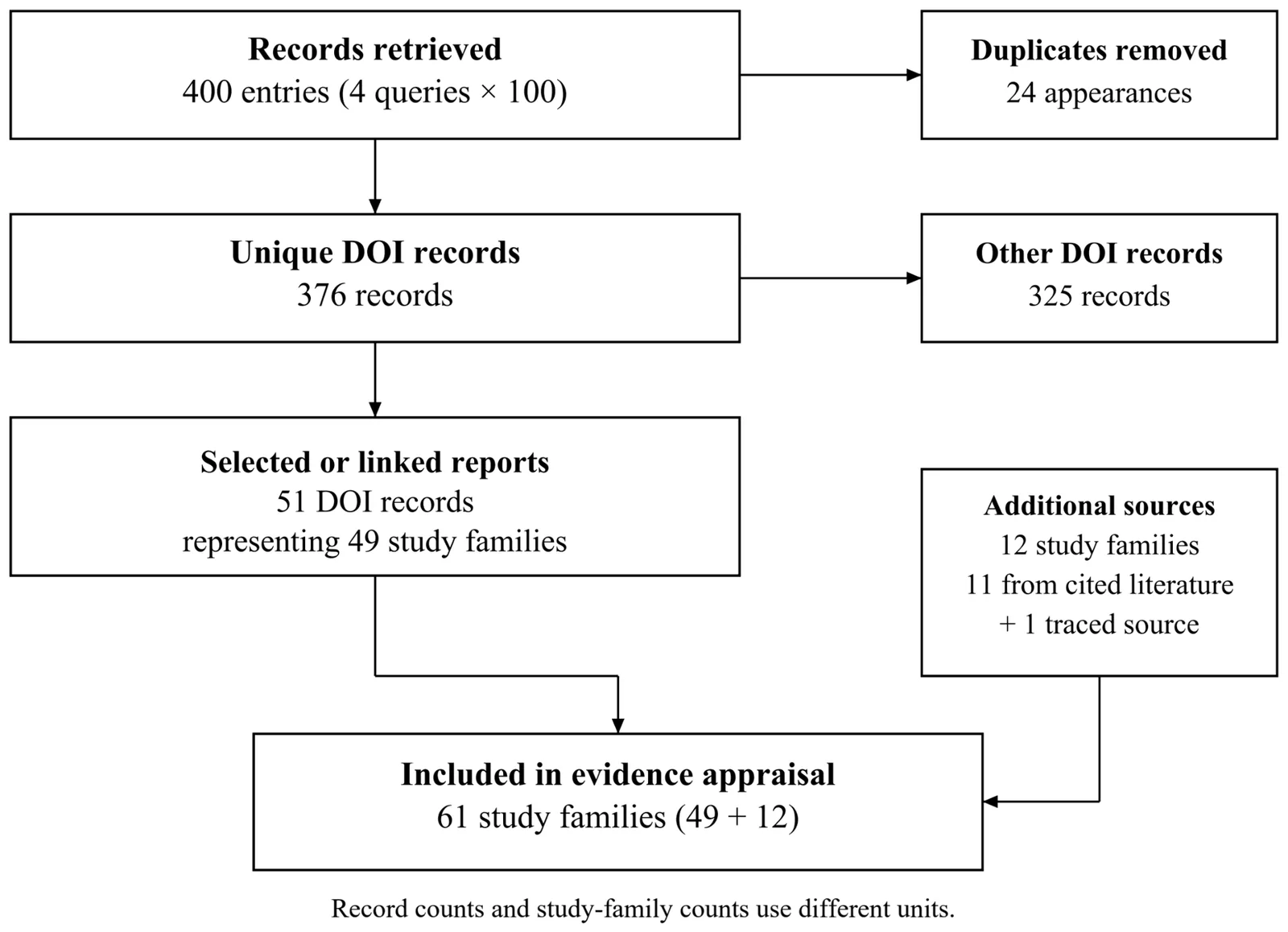
Literature-selection flow for the targeted critical evidence appraisal. Record and study-family counts use different units: 51 selected or linked DOI reports represent 49 protocol families after grouping a language version and reports sharing a protocol; 12 additional families give 61 selected families. The other 325 DOI records retain their recorded screening, contextual, eligibility or unresolved roles and are not collectively full-text exclusions.

Figure 1 shows the selection flow. The 400 retrieved entries yielded 376 unique DOI records after removal of 24 duplicate appearances. Fifty-one selected or linked report records represent 49 experimental or modeling families after shared protocols and a language version are grouped. Twelve additional families—11 from cited literature and one from targeted source tracing—give 61 selected families. A family is a documented protocol, not a count of independent experimental replicates. Selection required an identifiable material state and a locatable corrosion, hydrogen, mechanical, contact or assembly endpoint; comparisons were interpreted within their preparation, exposure, loading and control conditions. Tables S1 and S2 identifies the selected families, and Table S3 records study-specific appraisal details.

Crossref was the only database searched. English queries, incomplete indexing and the first-100 relevance cutoff can miss relevant work and influence the selected evidence. Citation checks add sources but do not remove this coverage limitation. The review is therefore a targeted critical appraisal, not an exhaustive systematic survey. The accompanying search records preserve the exact requests, raw responses and record dispositions.

Reports sharing specimens, a protocol or a language version are linked rather than counted as independent confirmation. Conflicting results are retained and interpreted against their own starting states and controls. The coated/stripped FIP study by Zhang et al. [8] is retained only as a preparation-and-loading-control case in Table S3; its fracture-time entries were not used quantitatively. Hydrogen methods and specimen histories are cross-referenced in Table S1M.

## 3. From Coating Protection to Local Steel Damage

A coated wire inherits its substrate state from drawing and thermal processing. Drawing refines and aligns the ferrite–cementite structure [9,10,11], contributes to strength [12] and introduces residual stress [13]. The coating morphology and inventory are therefore interpreted alongside the condition of the underlying steel. The evidence below separates manufacturing observations, visible-rust endpoints and measurements of the steel geometry that carries load.

### 3.1. Processing and Coating Structure

The principal coating families are Zn, Zn–Al and Zn–Al–Mg. These labels describe nominal chemistry, not measured phase fractions. Zhu’s two-temperature dipping comparison showed different Al-rich morphologies and reaction-layer structures [14]; its processing and strength details are recorded in the Supplementary Appraisal. Interfacial reactions provide a physical basis for considering bath temperature and thermal history alongside chemistry [15].

Corrosion-product studies on model Zn–Mg alloys [16], coated sheets [17] and finished Zn–Al–Mg wire [18] provide different levels of evidence. The finished-wire study follows products and electrochemical responses during salt spray. Model alloys and sheets help interpret product chemistry, while the wire establishes the relevant specimen geometry and surface history.

Visible-rust observations show substantial differences between tested preparations. Hu et al. reported first visible red rust after 8, 19, 24, 25 and 57 days for five Zn, Zn–Al and Zn–Al–Mg preparations within a reported 50–60 µm thickness band [19]. Zhao’s Zn and Galfan products, with reported coating masses of 336 and 337 g/m^2^, first showed sampled brown products at 384 and 1016 h [20]. Table 1 preserves these product and preparation endpoints; they are not a common composition-only ranking.

**Figure 2:**
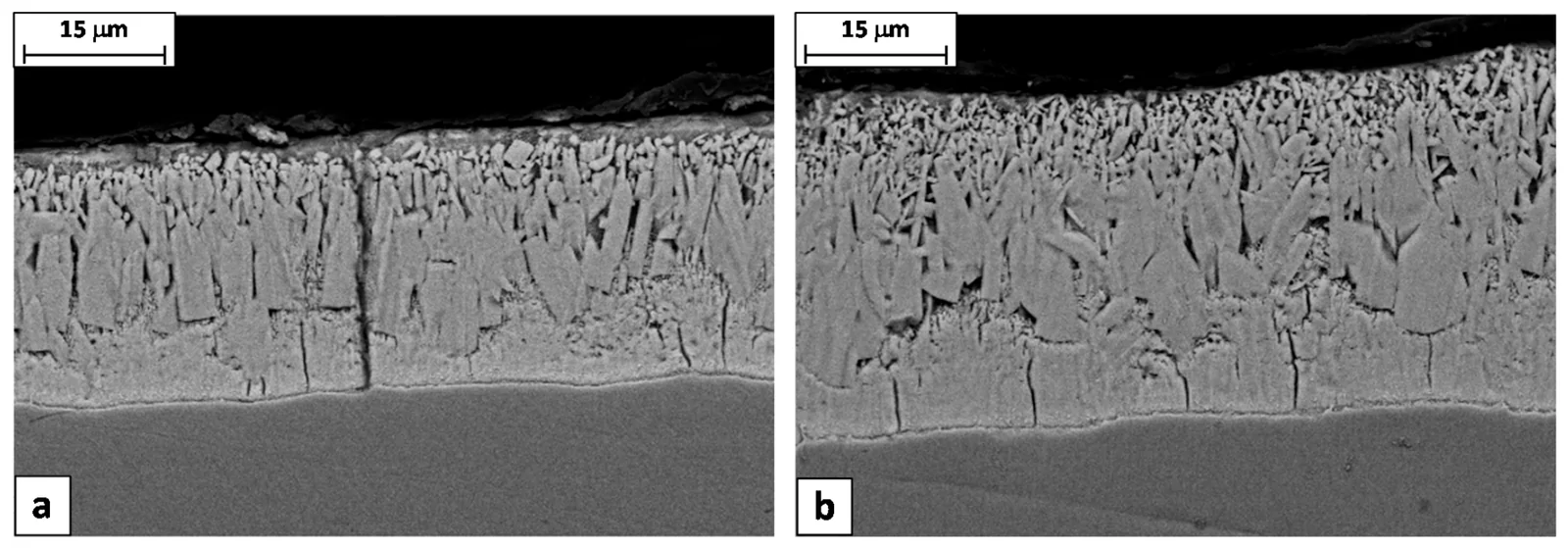
Longitudinal coating-section SEM images of galvanized C82D samples from the same steel batch before wire drawing: (**a**) standard air–water spray cooling; (**b**) spray switched off. Source panel labels and 15 µm scale bars are retained. Reproduced from Gelfi et al. [21], Figure 11; © 2017 the authors, CC BY 4.0.

Manufacturing observations also show that coating defects can precede environmental attack. In Gelfi’s galvanize-before-drawing C82D route, the same-batch air–water-spray comparison showed through-thickness coating cracks with spray cooling and cracks confined to the delta layer when the spray was switched off [21] (Figure 2). These observations make the initial coating state part of the comparison, alongside the chemistry and amount of coating.

### 3.2. Coating Reactions and Electrochemical Measurements

Zinc-based coatings protect by limiting electrolyte contact and by dissolving sacrificially when electrically connected to exposed steel [22]. Corrosion products modify that reacting surface [23]. A discontinuous coating can still participate in the electrochemical response. Polarization resistance, impedance and a Stern–Geary current estimate [24] consequently describe the combined specimen under the measurement assumptions, rather than the remaining load-bearing steel section.

Sacrificial protection requires a zinc-rich net anode, electrical continuity with steel and a shared conductive electrolyte. Surface films can change this polarity. Szabó and Bakos proposed atmospheric Zn/steel reversal involving zinc passivation and oxygen activation by corrosion products [25], with the oxygen-activation interpretation subsequently reiterated [26]. In a reversal, steel becomes the net anode; zinc depletion or electrical disconnection can end protection without reversal. Identifying the anodic member requires galvanic-current direction under stated film, wetting and oxygen conditions, supported by Zn- and Fe-specific dissolution measurements. This proposed explanation is not a demonstrated reversal in the bridge-wire tests appraised here.

The reference area in j = I/Aref must be stated: nominal wetted area, exposed steel area or another defined area. Mass-loss and electrical-resistance comparisons [27], imposed-current wire tests [28] and polarization/impedance measurements on coated preparations [19] describe the overall response without separating zinc and iron contributions. The complementary measurements answer different questions. Coating thickness or mapped continuity identifies the coating state. Constituent-resolved release and registered surface maps help identify the reacting material, while local Fe-bearing products provide spatial chemical evidence. Remaining steel geometry and a matched force response address mechanical protection.

Product comparisons must retain the coating inventory and substrate geometry. The galvanized and Zn–Al wires in Liu et al. differed in nominal diameter and coating thickness [29]. Such observations concern the tested product configurations, with their preparation and loading details kept in the Supplementary Appraisal.

### 3.3. Wetting and the First Indications of Steel Corrosion

A coating defect is part of an electrically connected surface: the electrolyte can link exposed steel to surrounding zinc. Confined-site models describe changes in pit chemistry and repassivation [30]. Thin-film transport [31,32], rust-layer reactions [33] and chloride-dependent iron oxyhydroxide formation [34] show why water retention and oxygen supply matter alongside nominal salt concentration and humidity.

Jarwali et al. provide a direct wetting comparison in 19-wire Zn- and Zn–10Al-coated strands at 40 °C after application of 0.01% NaCl [35]. Examined surfaces remained largely intact at 60% RH through 150 days. At 100% RH, attack was confined to the coating without visible iron rust; continuously wet gauze produced greater damage, including red rust on Zn-coated strands at 150 days. These observations distinguish persistent local wetting from high bulk-air humidity. Field guidance likewise directs inspection to water entry, failed sealing, low points, saddles and anchorages [36].

**Figure 3:**
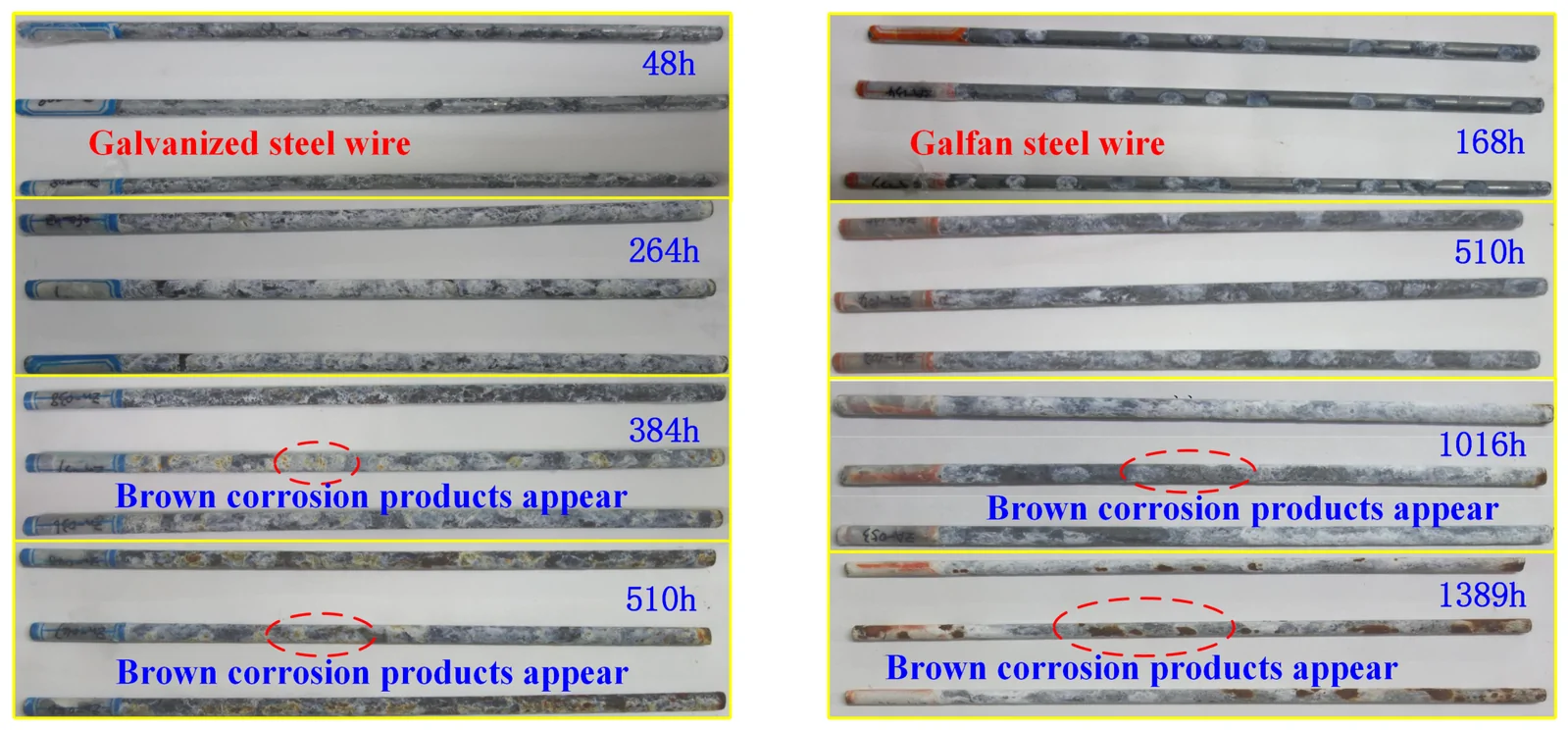
Galvanized and Galfan finished-wire surfaces sampled during neutral salt spray. The source-marked brown products first appear at 384 and 1016 h, respectively. These are sampled visible-product endpoints for different products and specimens. Adapted from Zhao et al. [20], Figure 3 (two source image objects joined; pixels and annotations unchanged); © 2023 the authors, CC BY 4.0.

Accelerated loaded/unloaded comparisons report earlier visible rust in the more highly loaded groups [37] or under cyclic loading [38]. Zhao’s sampled product photographs (Figure 3) illustrate the visible-rust endpoint [20]. These observations follow changes in surface appearance; first visible rust and the first microscopic steel attack are different events.

Local observations can precede an average coating endpoint. In Galfan wire exposed to continuous salt spray, Yuan et al. identified local Fe-bearing products during 510 < t ≤ 784 h [39], before the source’s later 1088 h criterion based on average cleaned mass-loss depth and appearance. This contrast supports measurement of the damage site alongside the average coating inventory; the selected studies do not supply a transferable thickness threshold for first steel attack.

### 3.4. Local Damage and the Load-Bearing Steel Section

After steel attack, mass loss, pit geometry and minimum remaining section describe different aspects of damage. Simulated acid-rain tests on polished wire regions linked static loading conditions to endpoint mass loss and iron-bearing products [40]; another stressed-wire study varied solution conditions [41]. Both concern corrosion under load, distinct from fatigue of a separately pre-corroded wire.

Scanned pits and minimum sections have been related to tensile response in finished galvanized wires [42], while a separate salt-spray series recorded declining strength and elongation with section loss [43]. Recovered and subsequently re-exposed wires describe an already aged starting state [44]. In recovered-wire fatigue tests, pitted specimens had shorter lives than uniformly corroded specimens under matched study loads [45]. These results make the local steel geometry relevant to mechanical interpretation, beyond visible rust or whole-specimen current.

**Figure 4:**
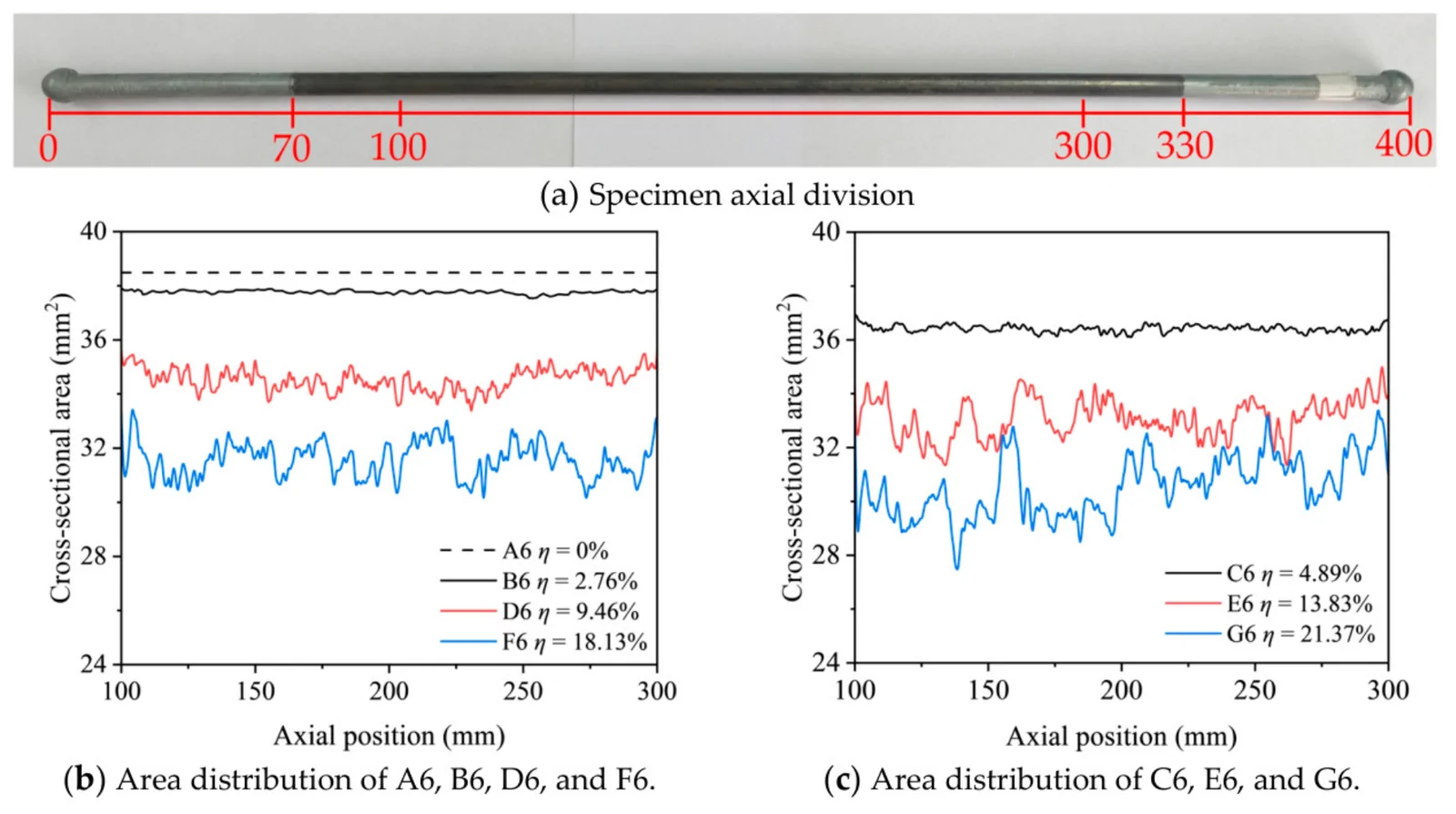
Axial cross-sectional-area profiles of 7 mm, 1770 MPa grade galvanized wires after electrochemical pre-corrosion. (**a**) Specimen and central analysis region. (**b**,**c**) Profiles sampled at 0.2 mm intervals from individual specimens A6–G6. Axial position is in mm; η denotes the source figure’s mass-loss labels. Reproduced from Yang et al. [46], Figure 7; © 2026 the authors, CC BY 4.0.

Axial profiles show where the remaining section is smallest. Yang et al. reconstructed profiles along electrochemically pre-corroded 7 mm wire [46] (Figure 4), sampling at 0.2 mm intervals over 200 mm. The displayed profiles represent one selected wire per corrosion group. Geometry thus adds spatial information to total mass loss.

Pit depth d is measured normal to a reconstructed uncorroded surface; width w requires a stated axial or circumferential direction, giving d/w. Spacing requires a center-to-center or edge-to-edge definition. Asteel(x) denotes remaining steel area and Amin its minimum over the examined length; local section loss is 1 − Asteel(x)/Asteel,0. Zinc and deposits are excluded from load-bearing area. Under a common axial force F, F/Amin is the largest section-averaged axial stress in the measured segment. Pit-root stress additionally depends on curvature and neighboring defects. Projected area and √area require a stated plane. These geometric quantities describe the resulting steel damage, whereas ISO 9223 classifies atmospheric corrosivity [47].

## 4. Hydrogen Supply, Trapping and Mechanical Response

### 4.1. Surface State and Hydrogen Measurements

Hydrogen entry depends on the reacting surface and its environment. Permeation follows hydrogen transported through a specimen [48]. Wet–dry tests on iron show that uptake can peak during drying as surface pH and corrosion potential change [49], and atmospheric uptake has been measured in high-strength steel [50]. These observations explain why an intact coating, corrosion products and exposed steel must be distinguished when discussing hydrogen supply to a galvanized wire.

The measurements describe different parts of that history. Diffusible hydrogen is released or transported in a specified extraction or permeation procedure; retained hydrogen remains after a stated transfer or desorption treatment. A trapped population exchanges more slowly with the lattice on the relevant timescale. Thermal desorption measures release during heating, permeation measures downstream transport, and atom-probe tomography (APT) locates detected species in a prepared tip. A controlled change in mechanical response when hydrogen supply or removal is varied supports hydrogen involvement in that response. Table S1M records the specimen, supply, transfer and loading histories needed to relate these measurements.

Finished-wire corrosion and imposed charging give complementary evidence. Nakamura and Suzumura measured less than 0.2 ppm low-temperature released hydrogen in their corrosion-level-2 wires, with and without tension [51]. Cao et al. observed section-related mechanical loss without hydrogen-induced or stress-corrosion cracking in their acidic wet–dry protocol [52]. Recio et al. instead observed hydrogen evolution, released diffusible hydrogen and ductility loss in galvanized prestressing wire and strand exposed to highly alkaline pore solution at 80% of ultimate load [53]. Hydrogen effects therefore depend on surface chemistry and mechanical history. The 0.7 ppm comparison in [51] remains specific to its wire and measurement protocol.

### 4.2. Hydrogen Supply During Loading

Controlled supply tests establish that pearlitic steel can become mechanically more susceptible as hydrogen availability increases. In notched cold-drawn pearlitic prestressing steel, fracture-initiation stress decreased as permeation-derived diffusible hydrogen increased [54].

Jia et al. distinguished precharging before loading, charging during loading, and combined precharging and continued charging in specimens machined from drawn wire [55]. In separate parameter series, greater elongation loss was associated with longer precharging at fixed current during loading, or higher current at fixed precharging duration. Similar responses under in situ and combined charging conditions show the importance of continued supply during deformation. Transfer and outgassing between precharge and loading change the hydrogen available at the start of a test. These imposed-charging experiments examine substrate susceptibility; applying their findings to atmospheric exposure requires corresponding surface and supply conditions.

Desorption provides a complementary mechanical contrast. Huang et al. charged unstrained and highly strained pearlite for 24 h at 50 mA/cm^2^ in 0.1 M NaOH + 1 g/L NH_4_SCN [56]. Mean elongation was 7.80% and 5.30% in the charged groups and 9.90% and 5.90% in separate groups tested after 12 h of room-temperature vacuum desorption. The desorbed groups approached their own uncharged references of 10.03% and 6.20%. Each condition comprised three tensile specimens; gauge lengths were 50 and 10 mm for the two material states. The within-state recovery supports hydrogen participation, while the different processing states and gauges define separate comparisons.

Table 2 gives means, sample standard deviations and ranges calculated from the three displayed individual observations. Charged-state loss is referenced to each state’s own uncharged condition. Source-reported percentages are retained separately from calculations using rounded individual values; the numerical and preparation details are documented in the Supplementary Appraisal.

### 4.3. Retention and Trapping in Drawn Pearlite

**Figure 5:**
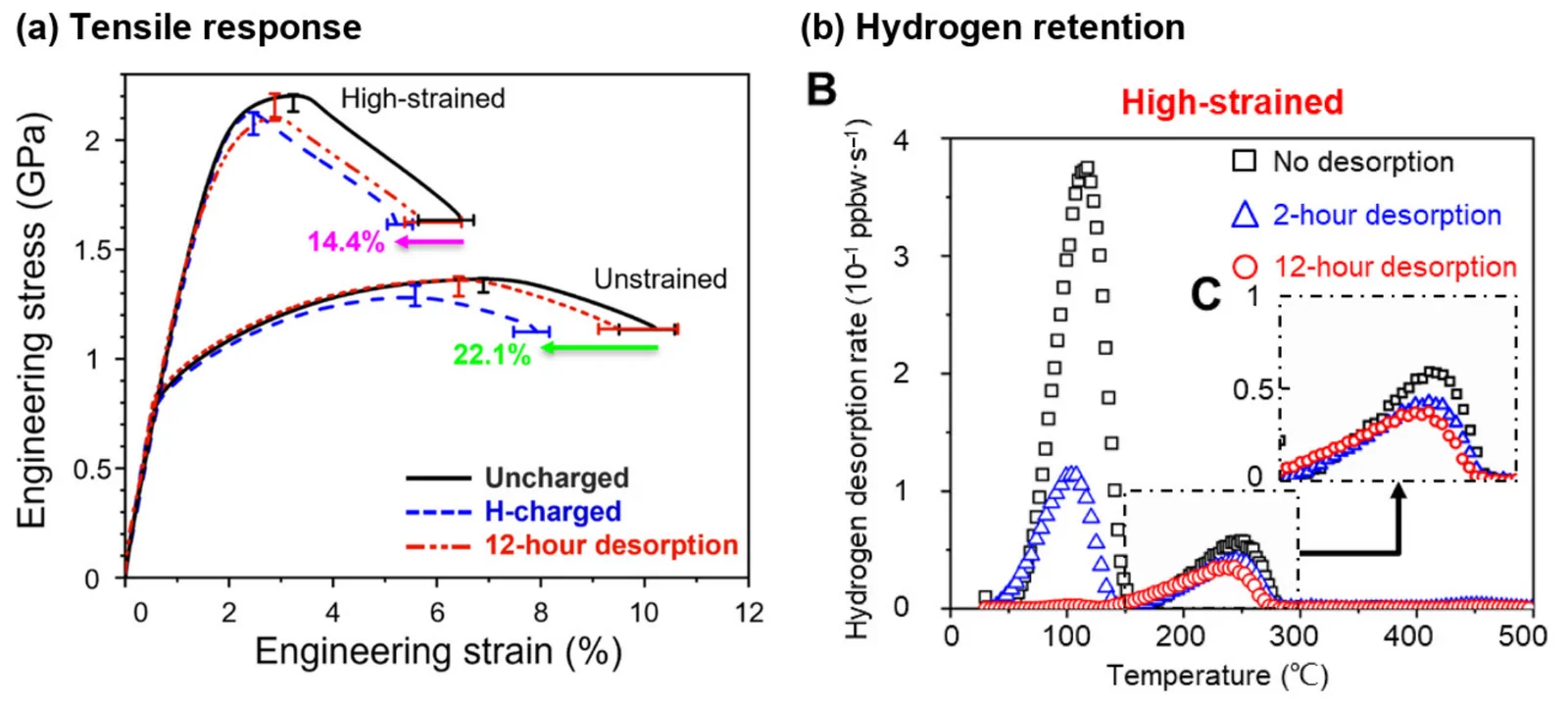
Mechanical response and hydrogen retention in pearlitic steel [56]. (**a**) Stress–strain curves for unstrained and highly strained specimens tested uncharged, after charging, or after charging and 12 h of room-temperature vacuum desorption; bars show ranges of tensile strength and elongation from three tests. (**b**) TDS spectra from separately prepared highly strained specimens after 0, 2 and 12 h desorption, including the source high-temperature-peak inset. The black series is uncharged in (**a**) and charged without a desorption hold in (**b**). Charging: 24 h at 50 mA/cm^2^ in 0.1 M NaOH + 1 g/L NH_4_SCN. Panels rearranged from Huang et al. [56], Figures 4 and 3B; © 2025 The Author(s), CC BY 4.0.

Hydrogen participation and the effect of a retained population can be examined separately. In Huang et al., ductility recovery after desorption accompanied reduced low-temperature release in independently prepared TDS specimens, while a high-temperature peak persisted [56] (Figure 5). The experiments establish tensile-ductility recovery and hydrogen retention under their respective specimen histories. Hu’s review interprets the lower susceptibility of highly drawn pearlite in terms of strong trapping by tangled dislocations [2]. A selective-desorption mechanical control tests the retained population more directly.

Doshida and Takai applied that control to 1838 MPa pearlite [57]. After charging, a 200 °C, 45 min hold removed the source-defined peak 1 hydrogen while retaining approximately 2.6 mass ppm peak 2 hydrogen. The peak-2-only specimens gave stress–strain curves overlapping those of an uncharged control given the same heat treatment. Specimens containing both peaks showed greater ductility loss at −30, 30 and 70 °C; the 30/70 °C tests continued solution charging during loading. This result distinguishes the mechanical relevance of the two source-defined populations with a matched thermal history.

APT adds spatial evidence for retention. Huang et al. located deuterium beside carbon-rich lamellae after 2 h of room-temperature vacuum desorption in independently prepared tips [56]. They associated these locations with dislocation tangles near ferrite–cementite interfaces. The observation supports trapping in the deformation-refined interfacial region. This spatial evidence complements the desorption and mechanical results, which were obtained from separately prepared specimens (Table S1M).

Peak shifts and local retention also require different physical interpretations. A peak-shift activation estimate, a bulk-transport model input and an effective APT-tip detrapping parameter describe distinct quantities [56]. Heating rate, diffusion distance, occupancy and retrapping affect release. The Supplementary Appraisal retains the numerical energy estimates and their methods, so these values are not combined into a single trap-binding energy.

### 4.4. Processing, Local Deformation and Orientation

Hydrogen-enhanced localized plasticity (HELP) describes changes in dislocation motion that concentrate deformation [58], while hydrogen-enhanced decohesion (HEDE) describes reduced separation resistance where hydrogen accumulates [59]. Adsorption-induced dislocation emission is another interpretation considered for precharged pearlitic specimens [55]. These descriptions guide interpretation of deformation and fracture features, rather than assigning a unique mechanism to every hydrogen-affected wire.

Charged pearlitic specimens showed reduced necking, peripheral cracks, lamellar shear and strain concentration near ferrite–cementite interfaces [55]. Crack paths followed colony or nodule boundaries and also crossed lamellae; direct interfacial separation was uncommon in the reported observations. These features support a contribution from localized deformation. The associated single-crystal-ferrite simulation concerns a simpler material state, distinct from the measured pearlitic fracture response.

Processing changes the combination of strength, residual stress and hydrogen response. Stabilization of drawn wire reduced strength and measured surface-ferrite residual stress while increasing the reported thiocyanate fracture time [60]. The treatment and loading reference changed together. This comparison concerns the stabilized state as a whole, with the X-ray measurement confined to surface ferrite.

Aging gives a contrasting strength trend. Hirakami et al. found higher tensile strength and higher charged/uncharged fracture-stress ratios after 10 min aging at 100 or 300 °C [61], together with lower hydrogen associated with the approximately 100 °C desorption peak. Stabilization and aging thus improved their respective hydrogen-related mechanical responses despite opposite strength changes. Strength alone therefore does not rank the hydrogen-related responses in these processing comparisons.

Tomatsu et al. tested miniature specimens machined from drawn-and-annealed wire under in situ plasma charging conditions [62]. Charged axial specimens sustained higher stresses than the charged transverse groups. Specimen geometry and fracture images indicate that the nominal fracture planes of the transversely loaded specimens contained the drawing direction, with local deviations in the crack paths. One of the three fractured axial specimens developed a longitudinal stationary subcrack; this does not establish a common longitudinal final-fracture plane. For axial wire tension, bending or contact, the candidate plane must be interpreted through its resolved normal and shear tractions; the experiment does not determine a universal contact-crack angle.

## 5. Fatigue, Contact Damage and Sustained-Load Cracking

### 5.1. Defect Geometry and the Loading Reference

Interpreting fatigue comparisons requires a defined initial defect and loading reference. Here, the stress range is Δσ = σmax − σmin, amplitude is σa = Δσ/2, and R = σmin/σmax. Force divided by the initial steel area gives nominal stress; division by the measured remaining area gives net-section stress. Neither quantity specifies the local pit-root or crack-tip field. At a fixed range, R still changes the maximum and mean stress. In environmental fatigue, elapsed time complements cycle count because the time available for reactions changes with loading frequency.

Defect depth is only one part of the starting geometry. Root curvature, width, spacing and orientation influence how a pit concentrates stress and develops into a crack. Defect-based fatigue descriptions relate small flaws to fatigue strength [63,64,65], while crack-growth laws describe propagation once a crack exists [66,67]. The local chemical and mechanical conditions can continue to evolve during the pit-to-short-crack transition [68]. Prepared flaws [69,70] and measured pit distributions [71] therefore address different starting states. Multiple-pit interactions [72] and residual-stress-dependent crack-front evolution [73] add features that an equivalent depth alone cannot represent.

**Figure 6:**
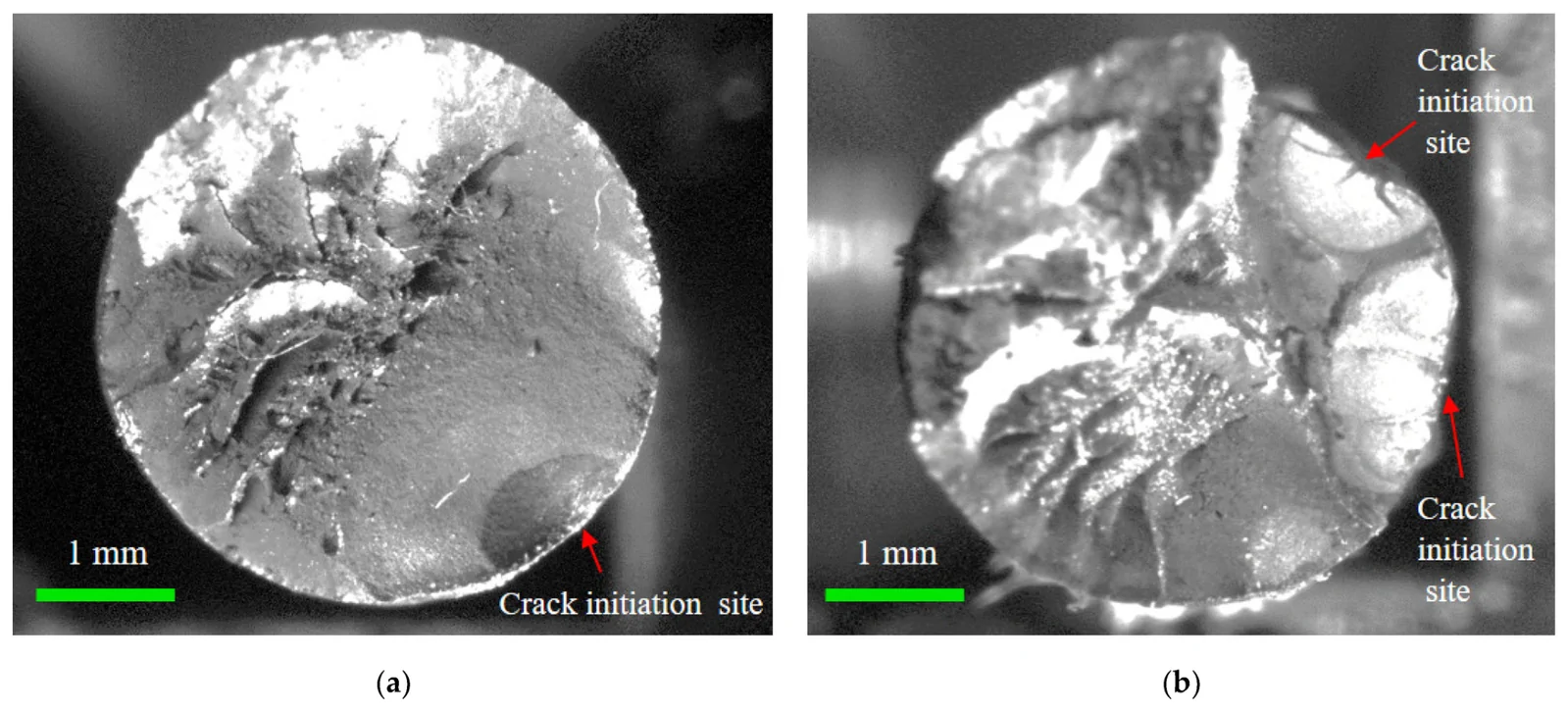
Optical fracture surfaces after salt-spray pre-corrosion and fatigue testing: (**a**) relatively flat fracture with one marked origin; (**b**) stepped fracture with two marked origins. Original annotations and 1 mm scale bars are retained. Reproduced from Liu et al. [29], Figure 12; © 2024 the authors, CC BY 4.0.

Fractography supplies a spatial link between the starting damage and the final failure. After salt-spray pre-corrosion, Liu et al. located fatigue origins near pit bottoms and documented both relatively flat, single-origin fractures and stepped, multiple-origin fractures (Figure 6) [29]. Deeper pits and multiple origins were associated with shorter total lives. These images show where fracture began; they do not time the initiation event separately from subsequent propagation. For fatigue assessment, the useful geometric record combines pit dimensions, neighboring defects, remaining section and the registered fracture origin.

Models likewise need an explicit starting state. Exposed-steel-window tests [74,75], imposed-potential wire-life tests [76], within-source life-model checks [77] and fitted beach-mark histories [78] assess different parts of the problem. A model that reproduces terminal life has been checked for that endpoint; the intervening pit and crack history needs its own observations. Table S3 identifies the preparation, experimental family and checked output for each series, while Section 7 separates wire-level and cable-level evidence.

### 5.2. Pre-Corrosion and Continued Environmental Exposure

**Figure 7:**
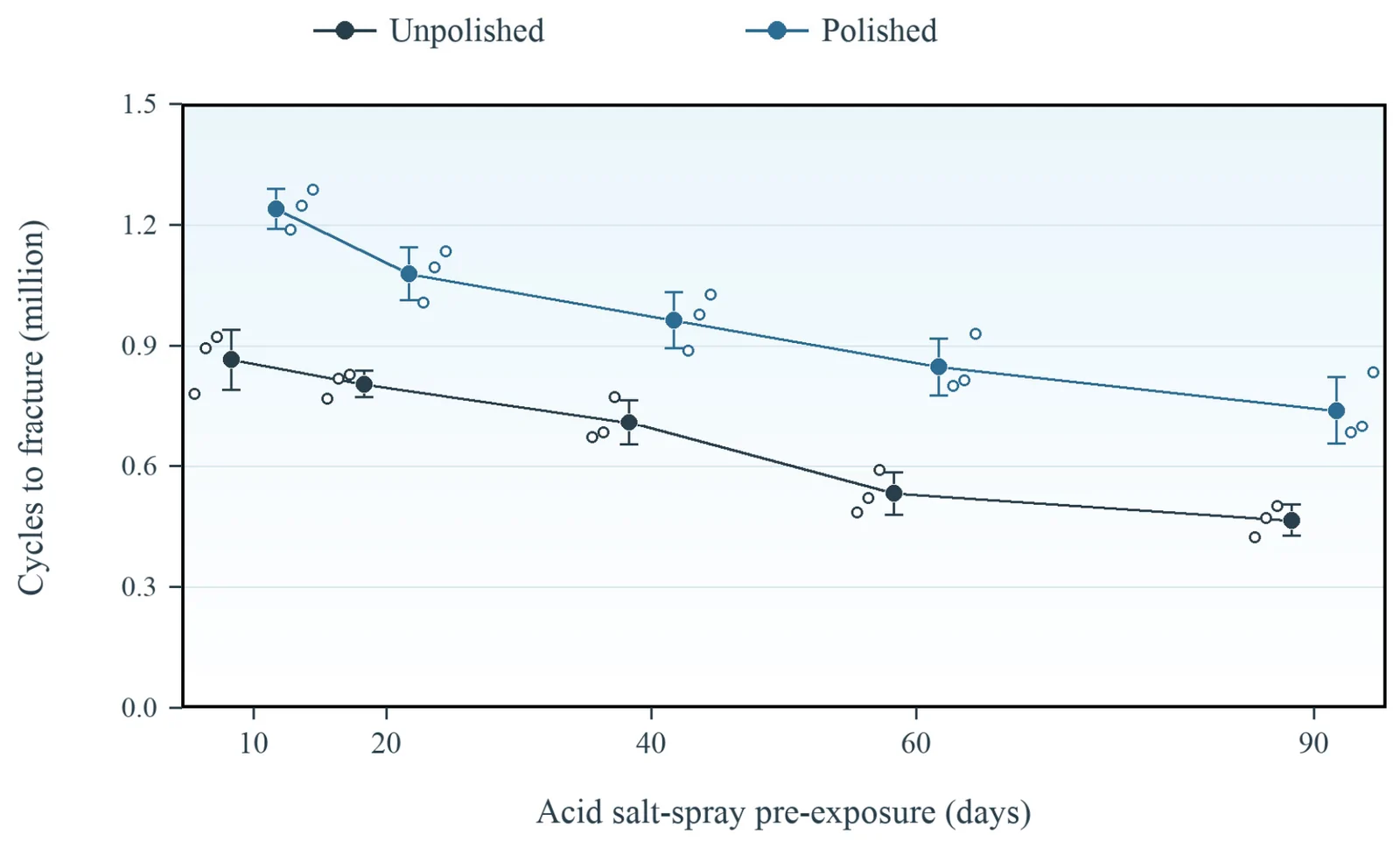
Fatigue after 10–90 days of acid salt spray for recovered 7 mm suspender wires, tested at 25 Hz and an approximately 360 MPa nominal stress range. Open markers show all 30 source-listed wires; filled markers and bars show mean ± sample SD for three different wires per group. Separate groups were polished or left unpolished before cycling. Data redrawn from Deng and Deng [79], Table A1; loading definitions are appraised in Table S3.

Pre-corrosion followed by cycling starts from inherited surface damage. Deng and Deng tested recovered 7 mm suspender wires after 10–90 days of acid salt spray, then loaded separate polished and unpolished groups at 25 Hz [79]. Figure 7 retains all 30 specimen-level records and the mean ± sample SD of three wires per group. Polished groups had 34–60% higher mean cycles. This prepared-state contrast combines the changed surface geometry and any change in steel section; the approximately 360 MPa nominal range is resolved from the source definition and reported loading quantities in Table S3.

Survival at a stopping limit is different from an observed fracture. The salt-spray studies of Liu et al. [29] and Yang et al. [46] include 2 × 10^6^-cycle runouts. These are retained as right-censored observations, Nf ≥ 2 × 10^6^, rather than assigned a failure life. When comparing damage levels or fitting fatigue curves, runouts must remain distinct from failures rather than being averaged with failure cycles.

Maintained wetting adds environmental action during loading. Nakamura and Suzumura compared wet and dry cycling at matched pre-corrosion grades [51]. Tests ran at 64 Hz and 100 mm grip spacing; dry cycling used relative humidity below 50%, and wetted gauze within a silicone tube maintained the wet condition. Fatigue strength was lower during wet cycling. At the same initial corrosion grade, the subsequent exposure history can therefore change the fatigue response.

**Figure 8:**
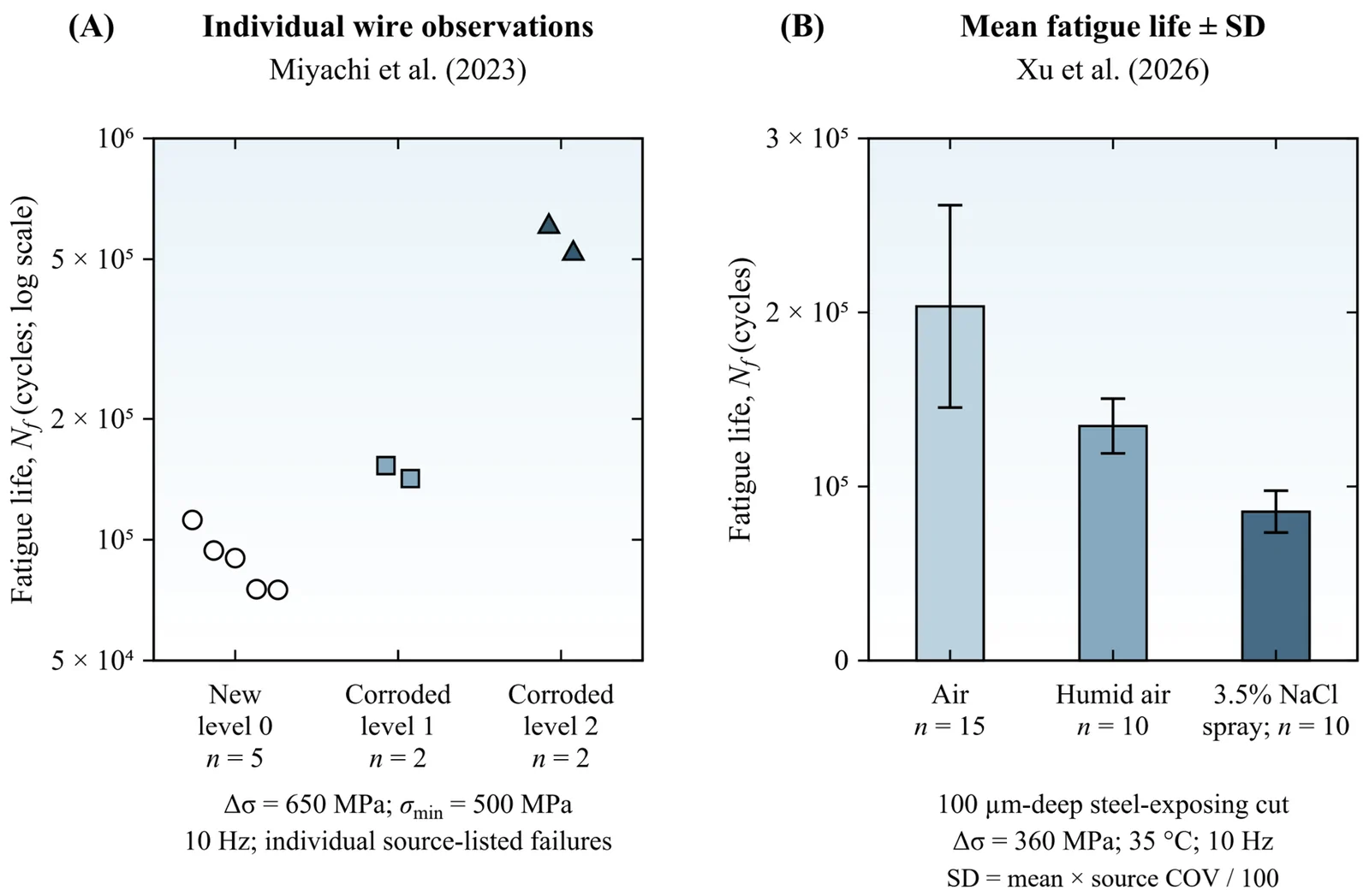
Two separate fatigue protocols. (**A**) Individual lives of five new and four 8300 h salt-spray-corroded 7 mm wires at Δσ = 650 MPa, σmin = 500 MPa and 10 Hz [80]; source: Table 8. (**B**) Mean life ± SD of 5.2 mm wires with a prescribed 100 μm deep steel-exposing cut at Δσ = 360 MPa, σmax = 860 MPa, 35 °C and 10 Hz: air (*n* = 15), 80–85% RH (*n* = 10), and 3.5% NaCl spray (*n* = 10) [84]; source: Tables 4 and 11. SDs are calculated from reported coefficients of variation. Panel (**A**) uses logarithmic cycles and panel (**B**) linear cycles.

The literature also contains reversed or non-monotonic orderings. Miyachi et al. reported 75,000–112,000 cycles for five new wires and 142,000–608,000 cycles for four wires after 8300 h of salt spray, at Δσ = 650 MPa, minimum stress 500 MPa and 10 Hz [80] (Figure 8A). Wang’s condition-labeled cycle entries are higher in 3.5% than in 0.5% NaCl at all four stress ranges [81]; they remain in Table S2A. Potential-controlled tests [82] and a prefatigue–corrosion-fatigue sequence [83] also produced non-monotonic responses. These observations are retained without imposing a general benefit of corrosion, salt concentration or prefatigue; their unresolved causes are recorded in Table S3.

A prepared steel-exposing cut provides a more narrowly defined environmental comparison. Xu et al. tested 5.2 mm wires with a 100 μm deep cut in air, 80–85% relative humidity or 3.5% NaCl spray at 35 °C and 10 Hz [84]. At Δσ = 360 MPa, with σmax = 860 MPa and σmin = 500 MPa, mean lives were 203,498, 134,754 and 85,538 cycles, respectively (Figure 8B). Group sizes were 15 in air and 10 in each wet environment. The result establishes environmental sensitivity after deliberate steel access. It measures endurance of that damaged state, rather than the duration of protection by an initially intact coating.

### 5.3. Coating Deterioration During Cycling

Cyclic loading can also change the coating before the steel is broadly exposed. Under Li et al.’s 20 mA/cm^2^ anodic-polarization protocol in 3.5% NaCl, loaded wires developed coating cracks and reached a source-defined potential-transition endpoint earlier than unloaded wires [85]. The reported endpoint time also increased across the tested 1, 5 and 10 Hz series. This frequency observation is specific to the imposed-current protocol. Table S2B retains the endpoint definition, all reported times and the arithmetic time–cycle–charge conversions; those conversions are not plotted as separate findings. Fatigue after salt-spray corrosion and pickling [86] begins from a different surface state.

### 5.4. Contact Damage and Fretting Fatigue

Relative motion at wire contacts deforms and redistributes zinc, generates debris and changes the stresses transmitted into steel. Normal force, crossing angle, confinement and displacement determine the contact configuration. Montalvo et al.’s interrupted tests on 4.85 mm Zn–5%Al wire found a crack while zinc remained elsewhere in the scar [87]. At the most damaging tangential load, the coated and stripped preparations had similar fretting-fatigue limits. The observations locate the relevant damage within the contact and motivate examination of the crack-origin region together with its remaining coating and slip condition.

Wear measures describe different aspects of that local response. Increasing imposed displacement increased total corrosion–wear volume but reduced the wear coefficient normalized by load and sliding distance in Wang et al.’s reciprocating-contact tests [88]. Surface EDS in saddle and interwire tests documented local zinc depletion and iron enrichment [89,90]. Under Liu et al.’s imposed-current contact protocol, mean fatigue life fell from about 3.98 × 10^5^ to 1.60 × 10^5^ cycles between 0 and 0.015 A at a stated 0.88 mm displacement and 120 N normal load [91]. Dry saddle tests on different strand products also showed a wide spread of galvanized-wire lives [92]. These responses make the local configuration central to interpretation; Tables S1N and S3 retain the specimen and contact details.

### 5.5. Sustained Loading, Hydrogen Supply and Section Loss

Under sustained force, environmental reactions can change both material susceptibility and the section carrying the load. In acid-stripped bridge wire loaded at 70% of the source-specified maximum force in 20 wt.% NH4SCN at 50 °C, Zhang et al. observed a non-monotonic fracture-time response to imposed polarization [93]. Separate cathodically treated specimens released more low-temperature hydrogen than the unpolarized or selected anodic specimens. The series brings hydrogen supply and dissolution into the same mechanical problem, while its separate desorption and fracture preparations remain identified in Table S3.

The same experiment measured a remaining section of about 0.70A_0_ at fracture at +100 A/m^2^ [93]. At constant force, the corresponding mean net-section stress is F/(0.70A_0_), approximately 1.43F/A_0_. This mechanical consequence follows directly from the reduced section; local pit-root and crack-tip stresses additionally depend on shape, plasticity and residual stress. Related cold-drawn high-carbon-wire studies [94,95] reinforce the need to specify material and environment together. For coated bridge wire, local coating condition, remaining steel geometry and hydrogen-supply history provide the starting description for interpreting sustained-load response.

## 6. What the Evidence Establishes

The reviewed experiments establish four findings within their test conditions. First, coating preparations differ in when red rust or brown products are reported [19,20]. Table 3 separates product exposure, prepared coating breaches and fatigue after deliberate steel access, helping readers choose an endpoint for the performance being assessed.

Second, continued exposure changes fatigue after damage. Wires with a prescribed cut had lower mean lives in humid air and salt spray than in air [84]; another experiment found lower fatigue strength during wet cycling than during dry cycling at matched pre-corrosion grades [51]. Together, these comparisons show why fatigue assessment includes both the initial steel geometry and the environment maintained during loading.

Third, controlled charging and desorption alter the tensile response of the tested pearlitic states: charging reduces elongation, and 12 h vacuum desorption partly restores it [56]. Thermally matched selective desorption distinguishes the responses associated with the source-defined hydrogen populations [57]. These mechanical controls establish hydrogen participation and population-specific responses within their protocols; APT adds spatial information on retention.

Fourth, contact damage is localized. A crack was observed while zinc remained elsewhere in the contact scar [87], and imposed-current contact tests gave a lower mean fatigue life at the higher tested current [91]. Assessment therefore includes coating distribution and geometry at the damage site, contact force and relative motion.

## 7. Wire- and Cable-Level Evidence

At cable scale, the environment and force carried by each wire depend on its position, contacts and restraint. Sheathing and dehumidification influence moisture entry [96], while cable bands and saddles govern contact and force transfer [36,97]. Single-wire measurements describe local steel condition and resistance. Relating them to a cable response additionally requires spatial wetting, stiffness, boundary conditions and the positions of broken wires.

Published model checks support specific quantities. Yang et al. evaluated six terminal wire lives independently of calibration under a solution-exposed-window protocol [76], while Yuan et al. compared predicted yield and ultimate strengths with wire-level tests [98]. At cable scale, An et al. checked selected third and seventh wire-break cycles against two published corroded 69-wire cable tests [99]. Xue and Shen recorded the first nine breaks in a 121-wire cable under tension, bending and external wetting conditions [100]. Their restraint was adjusted to stress measurements from that same cable, so the comparison reproduces that assembly’s response. Table S1E identifies each check’s endpoint and relation to calibration.

**Figure 9:**
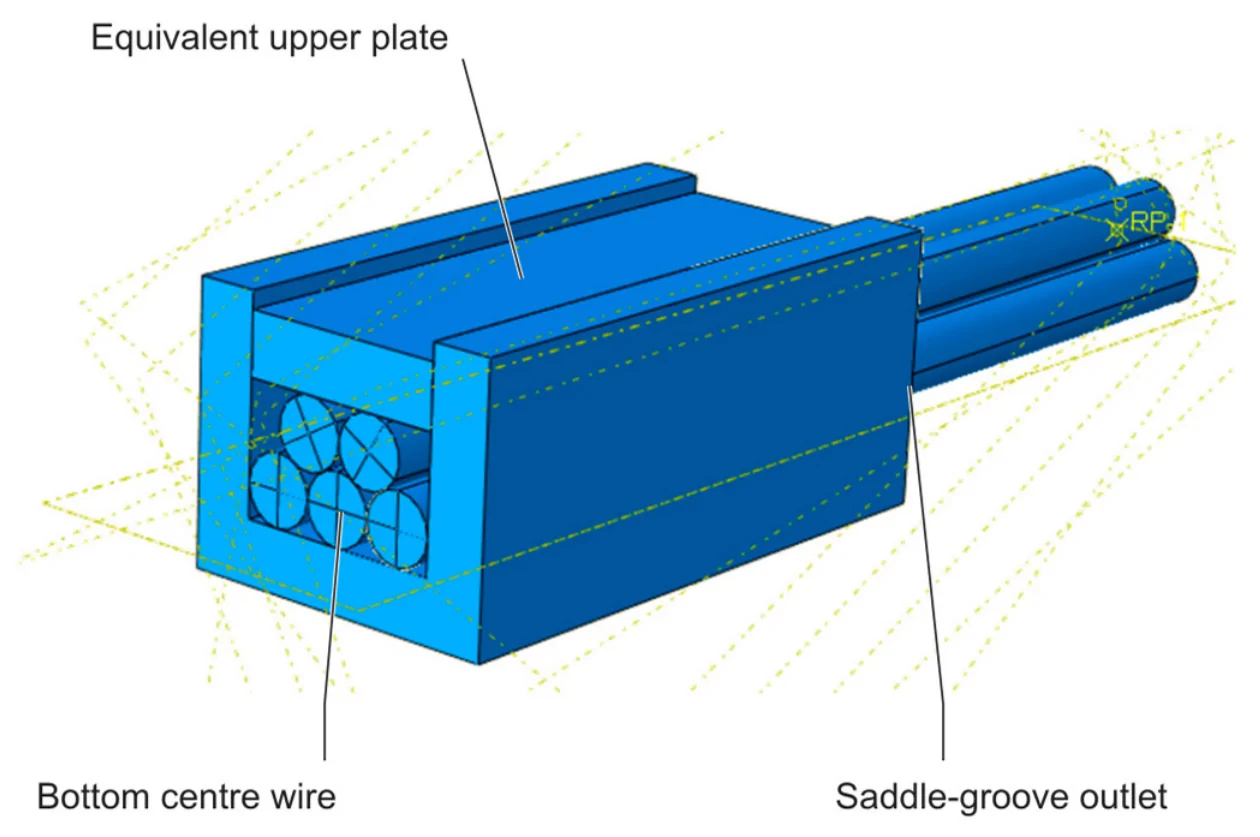
Local finite-element model of contact at a main-cable saddle outlet. Five 5.65 mm wires form two layers in a U-shaped groove; the upper plate represents overlying-wire pressure. The bottom wire–groove contact at the outlet is hidden in this view. Adapted from Li et al. [97], Figure 10 (enlarged; explanatory labels and leader lines added; source geometry and markings retained); © 2023 the authors, CC BY 4.0.

Geometry-specific models show how local responses can interact. Lan et al.’s parallel-wire stay model redistributes load after successive breaks under equal-sharing assumptions [101]. Li et al.’s saddle-outlet model places five 5.65 mm wires in two layers of a groove and locates the critical modeled fatigue site at the bottom wire–groove contact (Figure 9) [97]. Ding et al. used measured pit morphologies to define idealized strand defects; at fixed pit dimensions and prescribed end rotation, moving the pit toward the anchorage increased calculated stress concentration [102]. These examples identify contact location, restraint and defect position as necessary assembly inputs. Source-specific details are collected in Table S3.

Retained axial force, residual ultimate load-bearing capacity and fatigue life are separate assessment targets. Retained force describes the wire-force distribution under specified load or displacement, restraint and damage conditions and is checked against corresponding force measurements. Residual ultimate capacity is the maximum load sustained by the damaged assembly with a defined loading procedure and failure criterion; it requires ultimate-load evidence. Field frequency and deflection comparisons check bridge structural response [103], while wire-break records check break timing. Each validation measurement must correspond to the assessment target.

Engineering guidance supplies configuration-specific requirements: NCHRP Report 534 for main-cable inspection and strength evaluation [36], fib Bulletin 89 for prestressing-steel cable systems [104], PTI DC45.1 for stay cables [105], and BS EN 10264-2 for general wire-rope wires [106]. Electromagnetic tests on retired-wire bundles address corrosion detectability at the tested depth and condition [107]. A prospective panel-force study is proposed in Section 8 to connect local wire resistance with retained force under controlled damage and boundary conditions.

## 8. Future Research Directions

Further progress requires observations that connect the same local surface with its mechanical history. Interrupted tests on finished wires should map coating continuity, Fe-specific attack, pit geometry and crack detection to common surface coordinates, with a pre-exposure defect baseline and stated detection limits. Independent wires and processing runs must be distinguished from repeated scans or measurements on one specimen. These data would test how the established local responses interact during continued loading.

Controlled comparisons should use a common steel heat and finished-wire geometry while varying coating condition, deliberate steel exposure, hydrogen supply and loading. Surface-preparation controls should separate coating removal from acid and polishing effects. Bath composition, thermal history, cooling and coating thickness require separate contrasts when a composition or processing effect is claimed.

One prospective test could ask whether coating-continuity and first-iron-detection histories improve prediction of first subsequent substrate-crack detection beyond pit geometry, remaining steel section and load. At a fixed, predeclared landmark, only wires without a detected crack would enter the comparison, and both models would use information available at that time. Predictions would be evaluated in independent wire batches with a predeclared censoring-aware error measure. A minimum useful gain must be specified before fitting: an upper confidence limit below that gain would reject this protocol-specific prediction claim, whereas an interval spanning it would be inconclusive. Inspection intervals and detection limits define the endpoint; crack detection is not the physical instant of initiation. This untested proposal evaluates added predictive information, not the causal validity of a schematic sequence.

An engineering extension could test a specified main-cable panel between adjacent bands after a wire break. The target would be retained axial-force distribution under prescribed end displacement and cable-band restraint conditions. Local steel geometry and exposure/loading history would guide the selection of damaged-wire resistance measurements; break position, broken-end gap, spatial stiffness and tension recovery would supply assembly inputs. Predictions should be checked against independently measured wire forces from an assembly outside the calibration set. This proposed study concerns retained force; residual ultimate capacity requires a separately defined ultimate-load test.

**Figure 10:**
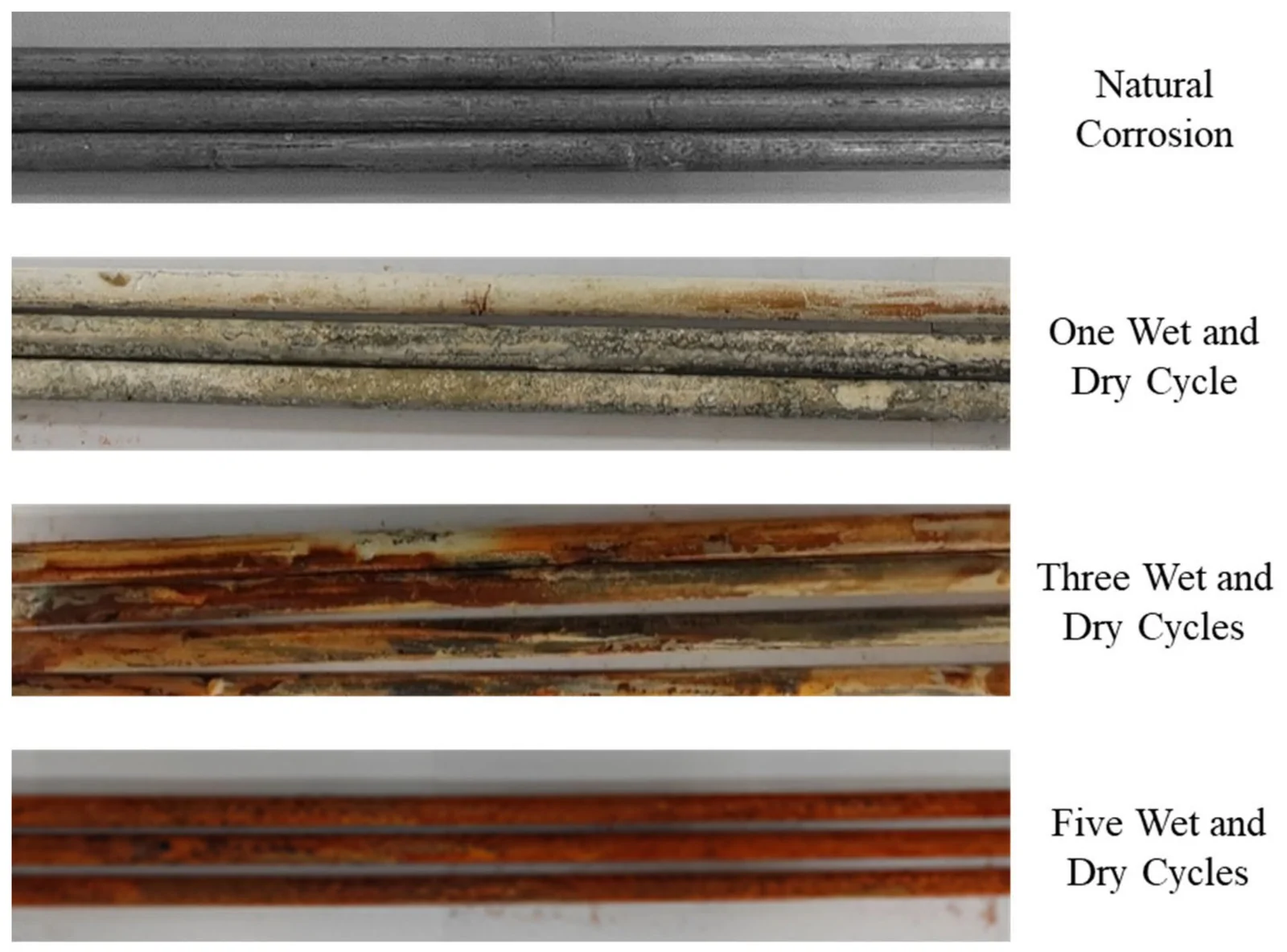
Recovered 7 mm galvanized stay-cable wires: as-recovered specimens and separate groups after one, three or five imposed-current wet/dry cycles. Each group contains three different wires; corrosion was present in the recovered baseline. Reproduced from Zhou et al. [109], Figure 4; © 2024 the authors, CC BY 4.0.

The starting state also needs to be measured in recovered and manufactured wires. Repeated wear measurements in a wire-contact experiment [108] describe locations within the tested specimens. The recovered-wire groups in Figure 10 already showed corrosion before additional accelerated exposure [109]. Drawing-sequence SEM likewise records inherited intercolonial damage [110]. Linking such observations to wire and processing-run identities would distinguish new environmental damage from defects present before the test. Additional wire-corrosion experiments and hanger and cable models are appraised in the source notes to Table S1 [111,112,113,114,115,116,117].

Shared records should retain individual pit topography, hydrogen signals with their measurement histories, force/displacement records, failures and runouts, and identifiers linking companion specimens. This would allow checks of both endpoint predictions and the intermediate damage history.

## 9. Conclusions

This critical evidence appraisal identifies four supported aspects of coated-wire durability: product-specific timing of visible corrosion products, mechanical response after local steel damage, hydrogen-history effects in the tested pearlitic states and localized contact damage.

Red rust or brown products appear at different times in the tested products and preparations under each exposure protocol. After steel exposure, defect geometry and remaining section influence the mechanical response; continued wetting can reduce fatigue endurance at fixed initial damage [51,84].

Charging–desorption contrasts establish hydrogen involvement in the tested pearlitic states. Selective-desorption controls distinguish the mechanical relevance of retained populations, while independently prepared APT tips characterize local retention [56,57]. Each observation is interpreted within its own specimen and loading history.

Local wire measurements can inform cable assessment when spatial wetting, contact, restraint and load transfer are specified. Predictions of retained axial force and residual ultimate load-bearing capacity each require validation of the corresponding assembly response. The proposed predictive tests and main-cable assessment study remain future research directions.

## Figures and Tables

**Table 1:** First visible red-rust (Hu) or brown-product (Zhao) observations in seven tested coating preparations or products. Exposure protocols and processing histories differ; these are source-specific endpoints rather than a cross-study ranking.

Source/Preparation	Exposure	Coating Inventory	First Sampled Endpoint
Hu [19], Zn	Acetate salt spray	50–60 µm selected area	8 d
Hu [19], Zn–5Al	Acetate salt spray	50–60 µm selected area	19 d
Hu [19], Zn–10Al	Acetate salt spray	50–60 µm selected area	24 d
Hu [19], Zn–6Al–1Mg	Acetate salt spray	50–60 µm selected area	25 d
Hu [19], Zn–10Al–3Mg	Acetate salt spray	50–60 µm selected area	57 d
Zhao [20], Zn	Neutral salt spray	336 g/m^2^	384 h
Zhao [20], Galfan	Neutral salt spray	337 g/m^2^	1016 h

**Table 2:** Elongation in the unstrained and highly strained pearlitic material states studied by Huang et al. [56] (three tensile specimens per condition).

Material State (Gauge)	Tensile State	Elongation Mean ± Sample SD (Range), %	Source-Reported Charged Loss, %
Unstrained (50 mm)	Uncharged	10.03 ± 0.57 (9.4–10.5)	—
Unstrained (50 mm)	H-charged	7.80 ± 0.36 (7.4–8.1)	22.1
Unstrained (50 mm)	12 h desorbed	9.90 ± 0.78 (9.4–10.8)	—
Highly strained (10 mm)	Uncharged	6.20 ± 0.52 (5.6–6.5)	—
Highly strained (10 mm)	H-charged	5.30 ± 0.26 (5.1–5.6)	14.4
Highly strained (10 mm)	12 h desorbed	5.90 ± 0.53 (5.5–6.5)	—

**Table 3:** Starting states, measured responses and the assessment questions addressed by selected experiments.

Test State and Source	Observed Endpoint	Source Result	Assessment Use
Different Zn/Galfan products [20]	First sampled brown products	Zn: 384 h; Galfan: 1016 h	Visible-product timing in finished products
Prepared coating defect [19]	Notch appearance/SEM–EDS at 500 h	Zn–Al–Mg: no visible red rust, Zn/Mg products; Zn–Al: red rust	Local surface response
100 μm deep steel-exposing cut [84]	Mean fatigue life at Δσ = 360 MPa	Air: 203,498; humid air: 134,754; salt spray: 85,538 cycles	Post-damage environmental fatigue
Pre-corrosion levels 2/3 [51]	Wet/dry fatigue strength	Wet lower than dry within grade	Continued environmental action

## Data Availability

No new data were created or analyzed in this study. Data sharing is not applicable to this article.

## Supplementary Materials

The following supporting information can be downloaded at: https://www.mdpi.com/article/10.3390/ma19214692/s1. Supplementary Evidence Appraisal and Source Records: Table S1, selected source families and source notes; Table S1E, endpoints and specimen/site linkage; Table S1F, evidence roles; Table S1G, candidate transitions and measurement gaps; Table S1M, hydrogen methods and specimen histories; Table S1N, selected fretting-fatigue records; Tables S2A–S2C, numerical fatigue and potential-transition records; Note S1H, alternative interpretations; Table S3 and Appraisal Notes S3, study-by-study appraisal; Notes S3H, S3P and S3L, desorption-energy quantities, comparison identities and equal-load-sharing arithmetic.

## References

[1] Jie Z., Zhang Z., Susmel L., Zhang L., Lu W. (2024). Corrosion fatigue mechanisms and evaluation methods of high-strength steel wires, a state-of-the-art review. Fatigue Fract. Eng. Mater. Struct..

[2] Hu C., Zhang M., Zhu X., Xue H., Lu P., Zhou F., Xu K. (2026). Hydrogen embrittlement in hard wire steel, mechanisms, influencing factors and future research directions. Mater. Technol..

[3] Zhao Y., Guo M., Feng H., Zhu Y., Huang P., Yuan Y. (2026). Corrosion fatigue degradation mechanisms and damage assessment methods of bridge cables, A state-of-the-art review. Constr. Build. Mater..

[4] Eiselstein L.E., Caligiuri R.D., Dean S.W., Lee T.S. (1988). Atmospheric corrosion of the suspension cables on the Williamsburg Bridge. Degradation of Metals in the Atmosphere.

[5] Roffey P. (2013). The fracture mechanisms of main cable wires from the forth road suspension. Eng. Fail. Anal..

[6] Barton S.C., Vermaas G.W., Duby P.F., West A.C., Betti R. (2000). Accelerated corrosion and embrittlement of high-strength bridge wire. J. Mater. Civ. Eng..

[7] Betti R., West A.C., Vermaas G., Cao Y. (2005). Corrosion and embrittlement in high-strength wires of suspension bridge cables. J. Bridge Eng..

[8] Zhang Z., Wang L., Song S., Tang L., Zhang H., Zhou H., Fang F. (2023). Effect of coating on stress corrosion performance of bridge cable steel wire. Coatings.

[9] Borchers C., Kirchheim R. (2016). Cold-drawn pearlitic steel wires. Prog. Mater. Sci..

[10] Li Y.J., Choi P., Borchers C., Westerkamp S., Goto S., Raabe D., Kirchheim R. (2011). Atomic-scale mechanisms of deformation-induced cementite decomposition in pearlite. Acta Mater..

[11] Zelin M. (2002). Microstructure evolution in pearlitic steels during wire drawing. Acta Mater..

[12] Zhang X., Godfrey A., Huang X., Hansen N., Liu Q. (2011). Microstructure and strengthening mechanisms in cold-drawn pearlitic steel wire. Acta Mater..

[13] Elices M. (2004). Influence of residual stresses in the performance of cold-drawn pearlitic wires. J. Mater. Sci..

[14] Zhu X., Shen Y., Ge Y., Zhang S. (2022). Effect of hot dip plating process parameters on microstructure and properties of zinc-10% aluminum-mischmetal alloy coated for bridge cable steel wire. Metals.

[15] Marder A.R. (2000). The metallurgy of zinc-coated steel. Prog. Mater. Sci..

[16] Prosek T., Nazarov A., Bexell U., Thierry D., Serak J. (2008). Corrosion mechanism of model zinc–magnesium alloys in atmospheric conditions. Corros. Sci..

[17] Holzner T., Luckeneder G., Strauß B., Valtiner M. (2022). Environmentally friendly layered double hydroxide conversion layers, formation kinetics on Zn–Al–Mg-coated steel. ACS Appl. Mater. Interfaces.

[18] Gu Z., Zhang Y., Zhang P., Wang Y., Fang F., Zhang D., Zhou D. (2026). Corrosion behavior of high-strength steel wire with hot-dipped Zn-Al-Mg coating in chlorine-containing environment. J. Electroanal. Chem..

[19] Hu D., Song S., Zhang Z., Wang L. (2023). Experimental study on corrosion resistance of Zn–Al–Mg alloy coating of high-strength steel wires for bridge cables. Anti-Corros. Methods Mater..

[20] Zhao Y., Su B., Fan X., Yuan Y., Zhu Y. (2023). Corrosion fatigue degradation characteristics of galvanized and Galfan high-strength steel wire. Materials.

[21] Gelfi M., Solazzi L., Poli S. (2017). Influence of the Manufacturing Process on Defects in the Galvanized Coating of High Carbon Steel Wires. Materials.

[22] Revie R.W., Uhlig H.H. (2008). Corrosion and Corrosion Control, An Introduction to Corrosion Science and Engineering.

[23] de la Fuente D., Castaño J.G., Morcillo M. (2007). Long-term atmospheric corrosion of zinc. Corros. Sci..

[24] Stern M., Geary A.L. (1957). Electrochemical polarization, I. A theoretical analysis of the shape of polarization curves. J. Electrochem. Soc..

[25] Szabó S., Bakos I. (2005). Regarding the polarity reversal of the zinc/steel galvanic couple. Corros. Rev..

[26] Szabó S. (2015). Metal corrosion and its relation to other fields of science. Int. J. Corros. Scale Inhib..

[27] Jiang W., Fan P., Wei C. (2024). Corrosion test method of cable wire for cable bridge. J. Highw. Transp. Res. Dev. Engl. Ed..

[28] Zhang H., Liu H., Chen F., Luo Y., Xiao X., Deng Y., Lu N., Liu Y. (2024). Microscopic characteristics and corrosion rate modeling in galvanized high-strength steel wires. J. Mater. Res. Technol..

[29] Liu Z., Guo T., Li W., Zhang Q., Cheng B., Correia J. (2024). Fatigue behavior and fracture surface analysis of corroded high-strength bridge cable wires. Materials.

[30] Galvele J.R. (1976). Transport processes and the mechanism of pitting of metals. J. Electrochem. Soc..

[31] Stratmann M., Streckel H. (1990). On the atmospheric corrosion of metals which are covered with thin electrolyte layers—II. Experimental results. Corros. Sci..

[32] Stratmann M., Streckel H. (1990). On the atmospheric corrosion of metals which are covered with thin electrolyte layers—I. Verification of the experimental technique. Corros. Sci..

[33] Stratmann M., Hoffmann K. (1989). In situ Mössbauer spectroscopic study of reactions within rust layers. Corros. Sci..

[34] Rémazeilles C., Refait P. (2007). On the formation of β-FeOOH (akaganéite) in chloride-containing environments. Corros. Sci..

[35] Jarwali T., Nakamura S., Suzumura K. (2014). Corrosion resistance of bridge steel wire strands galvanized with zinc-aluminium alloy. J. Jpn. Soc. Civ. Eng. Ser. A1.

[36] Mayrbaurl R.M., Camo S. (2004). Guidelines for Inspection and Strength Evaluation of Suspension Bridge Parallel Wire Cables.

[37] Li X.M., Zhou J.M., Liu Q., Kong L.F., Zhou J.T. (2007). Effect of Tension on Corrosion Behavior of Galvanized Steel Wire for Cable-Stayed Bridge. J. Electrochem..

[38] Guo Z., Chen H., Chen H., Yao G. (2026). Quantitative decoupling of load-environment effects on steel wire corrosion-fatigue degradation. Case Stud. Constr. Mater..

[39] Yuan Y., Liu X., Pu G., Wang T., Guo Q. (2021). Corrosion features and time-dependent corrosion model of Galfan coating of high strength steel wires. Constr. Build. Mater..

[40] Yang W.J., Yang P., Li X.M., Feng W.L. (2012). Influence of tensile stress on corrosion behaviour of high-strength galvanized steel bridge wires in simulated acid rain. Mater. Corros..

[41] Li R., Miao C., Wei T. (2020). Experimental study on corrosion behaviour of galvanized steel wires under stress. Corros. Eng. Sci. Technol..

[42] Yan K., Liu G., Li Q., Jiang C., Ren T., Li Z., Xie L., Wang L. (2024). Corrosion characteristics and evaluation of galvanized high-strength steel wire for bridge cables based on 3D laser scanning and image recognition. Constr. Build. Mater..

[43] Wan S., Zhou H., Li L., Wang C., De Filippo M., Gong F. (2022). Degradation of artificially corroded galvanized high-strength steel wires: Corrosion morphology and mechanical behavior. Constr. Build. Mater..

[44] Gao J., Zhang X., Wang H., Chen L. (2023). Random corrosion characteristics of high strength steel wire after 18 years of service and the numerical reconstruction method. Structures.

[45] Li R., Miao C., Feng Z., Wei T. (2021). Experimental study on the fatigue behavior of corroded steel wire. J. Constr. Steel Res..

[46] Yang G., Lu D., Jin L., Zou Y. (2026). Mechanical property degradation behavior and fatigue life analysis of corroded high-strength steel wires. Materials.

[47] (2012). Corrosion of Metals and Alloys—Corrosivity of Atmospheres—Classification, Determination and Estimation.

[48] Devanathan M.A.V., Stachurski Z. (1962). The adsorption and diffusion of electrolytic hydrogen in palladium. Proc. R. Soc. Lond. A.

[49] Tsuru T., Huang Y., Ali M.d.R., Nishikata A. (2005). Hydrogen entry into steel during atmospheric corrosion process. Corros. Sci..

[50] Akiyama E., Matsukado K., Wang M., Tsuzaki K. (2010). Evaluation of hydrogen entry into high strength steel under atmospheric corrosion. Corros. Sci..

[51] Nakamura S., Suzumura K. (2009). Hydrogen embrittlement and corrosion fatigue of corroded bridge wires. J. Constr. Steel Res..

[52] Cao Y., Vermaas G.W., Betti R., West A.C., Duby P.F. (2003). Corrosion and degradation of high-strength steel bridge wire. Corrosion.

[53] Recio F.J., Alonso M.C., Gaillet L., Sánchez M. (2011). Hydrogen embrittlement risk of high strength galvanized steel in contact with alkaline media. Corros. Sci..

[54] Enos D.G., Scully J.R. (2002). A critical-strain criterion for hydrogen embrittlement of cold-drawn, ultrafine pearlitic steel. Metall. Mater. Trans. A.

[55] Jia L., Hu C., Zhang M., Dong H., Zhu X., Cheng L., Xue H., Zhao J., Wu K. (2025). Interplay between H atoms and characteristic microstructure features in 2100 MPa grade full pearlite steel wire for bridge cable. Int. J. Hydrogen Energy.

[56] Huang C., Cui C., Niu R., Lu F., Wu C.-Y., Zhu X., Lu H., Zhang Y., Liu P.-Y., Dong B. (2025). Strong hydrogen trapping by tangled dislocations in cold-drawn pearlitic steels. Acta Mater..

[57] Doshida T., Takai K. (2014). Dependence of hydrogen-induced lattice defects and hydrogen embrittlement of cold-drawn pearlitic steels on hydrogen trap state, temperature, strain rate and hydrogen content. Acta Mater..

[58] Birnbaum H.K., Sofronis P. (1994). Hydrogen-enhanced localized plasticity–a mechanism for hydrogen-related fracture. Mater. Sci. Eng. A.

[59] Oriani R.A. (1972). A mechanistic theory of hydrogen embrittlement of steels. Ber. Bunsenges. Phys. Chem..

[60] Elices M., Ruiz J., Atienza J.M. (2004). Influence of residual stresses on hydrogen embrittlement of cold drawn wires. Mater. Struct..

[61] Hirakami D., Manabe T., Ushioda K., Noguchi K., Takai K., Hata Y., Hata S., Nakashima H. (2016). Effect of Aging Treatment on Hydrogen Embrittlement of Drawn Pearlitic Steel Wire. ISIJ Int..

[62] Tomatsu K., Chiba T., Chida T., Aoki T., Maeda N., Omura T., Hatta A. (2025). Anisotropy in Hydrogen Embrittlement Resistance of Drawn Pearlitic Steel Wire Evaluated by in-situ Miniature Test and Scanning Electron Microscopy during Plasma Hydrogen Charging. ISIJ Int..

[63] El Haddad M.H., Topper T.H., Smith K.N. (1979). Prediction of non propagating cracks. Eng. Fract. Mech..

[64] Murakami Y., Endo M. (1994). Effects of defects, inclusions and inhomogeneities on fatigue strength. Int. J. Fatigue.

[65] Sadananda K., Vasudevan A.K. (2020). Analysis of pit to crack transition under corrosion fatigue & the safe-life approach using the modified Kitagawa-Takahashi diagram. Int. J. Fatigue.

[66] Paris P., Erdogan F. (1963). A critical analysis of crack propagation laws. J. Basic Eng..

[67] Li C.X., Tang X.S., Xiang G.B. (2010). Fatigue crack growth of cable steel wires in a suspension bridge, Multiscaling and mesoscopic fracture mechanics. Theor. Appl. Fract. Mech..

[68] Turnbull A. (2014). Corrosion pitting and environmentally assisted small crack growth. Proc. R. Soc. A.

[69] Wang Y., Zheng Y.Q., Zhang W.H., Lu Q.R. (2020). Analysis on damage evolution and corrosion fatigue performance of high-strength steel wire for bridge cable, Experiments and numerical simulation. Theor. Appl. Fract. Mech..

[70] Jiang J.H., Ma A.B., Weng W.F., Fu G.H., Zhang Y.F., Liu G.G., Lu F.M. (2009). Corrosion fatigue performance of pre-split steel wires for high strength bridge cables. Fatigue Fract. Eng. Mater. Struct..

[71] Jiang C., Wu C., Jiang X. (2018). Experimental study on fatigue performance of corroded high-strength steel wires used in bridges. Constr. Build. Mater..

[72] Zhang H., Liu H., Deng Y., Cao Y., He Y., Liu Y., Deng Y. (2025). Fatigue behavior of high-strength steel wires considering coupled effect of multiple corrosion-pitting. Corros. Sci..

[73] Toribio J., Matos J.-C., Gonzalez B., Escuadra J. (2015). Influence of residual stress field on the fatigue crack propagation in prestressing steel wires. Materials.

[74] Lan C., Feng A., Zhang Y., Ma J., Wang J., Li H. (2024). Evaluation of corrosion fatigue life for high-strength steel wires. J. Constr. Steel Res..

[75] Bing H., Li S., Lan C. (2025). Investigation of the pitting-to-crack transition in corrosion-fatigue of high-strength steel wires based on the phase-field method. Int. J. Fatigue.

[76] Yang Y., Zhang Q., Xing S., Wang X. (2026). Peridynamic modeling and experimental validation of corrosion–fatigue process in high-strength steel wires. Int. J. Fatigue.

[77] Zheng Y., Wang Y. (2020). Damage evolution simulation and life prediction of high-strength steel wire under the coupling of corrosion and fatigue. Corros. Sci..

[78] Ma Y., He Y., Wang G., Wang L., Zhang J., Lee D. (2023). Corrosion fatigue crack growth prediction of bridge suspender wires using Bayesian Gaussian process. Int. J. Fatigue.

[79] Deng Y., Deng L. (2022). Corrosion fatigue test and performance evaluation of high-strength steel wires based on the suspender of a 11-year-old concrete-filled steel tube arch bridge. Coatings.

[80] Miyachi K., Saimoto S., Oki Y. (2023). Correlation between corrosion level and fatigue strength of high-strength galvanized steel wires used for suspension bridge cables. Bridge Struct..

[81] Wang Y., Zhang W., Zheng Y. (2020). Experimental study on corrosion fatigue performance of high-strength steel wire with initial defect for bridge cable. Appl. Sci..

[82] Lan C., Feng A., Zhang Y., Ding S., Ma J., Wang J. (2026). Generalized Probabilistic Model for the Corrosion Fatigue Life of High-Strength Steel Wires Considering Combined Effects of Corrosion and Alternating Stress. Fatigue Fract. Eng. Mater. Struct..

[83] Xue S., Shen R. (2025). Analysis of the Failure of High-Strength Steel Wires in Suspension Bridge Hangers under the Coupling Effect of Corrosion Fatigue after Prefatigue Load. J. Bridge Eng..

[84] Xu B.-H., Yao C., Zhang J.-N., Gong J., Zhang B. (2026). Influence of Corrosion, Fatigue, and Their Coupled Effects on High-Strength Galvanized Steel Wires. J. Mater. Civ. Eng..

[85] Li Y., Zhou H., Lin K., Xu S., Zhong X. (2025). Corrosion behavior of galvanized coating layer on high strength steel wire under fatigue load. Constr. Build. Mater..

[86] Liu Z., Guo T., Yu X., Huang X., Correia J. (2021). Corrosion fatigue and electrochemical behaviour of steel wires used in bridge cables. Fatigue Fract. Eng. Mater. Struct..

[87] Montalvo S., Fouvry S., Martinez M. (2024). Effect of zinc coating on the fretting fatigue endurance of crossed steel wires. Wear.

[88] Wang B., Wang D., Chong H., Xie G., Zhang D., Ge S. (2023). Tribo-corrosion interaction of the parallel steel wires in the suspension bridges. Friction.

[89] Wang B., Wang D., Tang L., Chong H., Xu W., Zhang X., Zhang C., Tan D., Zhao X., Zhang D. (2023). Bending tribo-corrosion-fatigue behaviors between cable wire and saddle material in the suspension bridge. Tribol. Int..

[90] Chong H., Wang B., Wang D., Ye J., Li C., Sun Y., Shen X., Zhang D. (2024). Tribo-corrosion-fatigue behaviors of the parallel wires of main cable in the suspension bridge. Wear.

[91] Liu Z., Guo T., Han D., Li A. (2020). Experimental study on corrosion-fretting fatigue behavior of bridge cable wires. J. Bridge Eng..

[92] Chehrazi A., Liang W., Huda N., Walbridge S. (2023). Fretting fatigue tests and microstructure analysis of bridge stay cable wires. Fatigue Fract. Eng. Mater. Struct..

[93] Zhang Z., Wang L., Huang W., Min X., Luo G., Wang H., Zhou L., Xie Z., Fang F. (2025). Stress corrosion cracking mechanisms in bridge cable steels, anodic dissolution or hydrogen embrittlement. Int. J. Hydrogen Energy.

[94] Wu S., Li J., Guo J., Shi G., Gu Q., Lu C. (2020). Stress corrosion cracking fracture mechanism of cold-drawn high-carbon cable bolts. Mater. Sci. Eng. A.

[95] Wu S., Chen H., Ramandi H.L., Hagan P.C., Crosky A., Saydam S. (2018). Effects of environmental factors on stress corrosion cracking of cold-drawn high-carbon steel wires. Corros. Sci..

[96] Furuya K., Kitagawa M., Nakamura S.-i., Suzumura K. (2000). Corrosion mechanism and protection methods for suspension bridge cables. Struct. Eng. Int..

[97] Li H., Zhou Z., Liu S., Wei L., Zhao J., Su H. (2023). Study on fatigue performance of 2200 MPa high-strength wire of main cables based on FE-SAFE. Coatings.

[98] Yuan Z., Wang H., Wang L., Li R., Mao J., Invernizzi S. (2025). Data-driven numerical simulation and evaluation methods for mechanical properties of corroded high-strength steel wire for bridge cable structure. Anti-Corros. Methods Mater..

[99] An Y., Li C., Mei Z., Xue S. (2026). Multiscale corrosion fatigue life prediction of parallel wire cables with stochastic pitting. J. Constr. Steel Res..

[100] Xue S., Shen R. (2024). Tensile–Bending–Corrosion Fatigue Analysis of the Parallel Steel Wire Cable in Suspension Bridges. J. Bridge Eng..

[101] Lan C., Xu Y., Liu C., Li H., Spencer B.F. (2018). Fatigue life prediction for parallel-wire stay cables considering corrosion effects. Int. J. Fatigue.

[102] Ding S., Peng X., Xu J., Ding D., Sun H., Cai S. (2026). Corrosion-induced stress concentration characteristics study of steel strands in bridge cables under tension-bending coupling loads. Materials.

[103] Zhao Y., Guo X., Su B., Sun Y., Li X., Bejinariu C. (2023). Evaluation of Flexible Central Buckles on Short Suspenders’ Corrosion Fatigue Degradation on a Suspension Bridge under Traffic Load. Materials.

[104] fib (2019). Acceptance of Cable Systems Using Prestressing Steels.

[105] Post-Tensioning Institute Recommendations for Stay Cable Design, Testing, and Installation.

[106] British Standards Institution (2022). Steel Wire and Wire Products—Steel Wire for Ropes—Part 2, Cold Drawn Non-Alloy Steel Wire for Ropes for General Applications.

[107] He D. (2023). Surface Corrosion Evaluation of Steel Cable for Suspension Bridge Using Electromagnetic Method. Surf. Sci. Technol..

[108] Wang B., Wang D., Ye J., Tang L., Chong H., Xu W., Zhang X., Chen J., Deng H., Zhang J. (2024). Effect of fatigue load on the bending tribo-corrosion-fatigue behaviors between the main cable wires. Friction.

[109] Zhou L., Yao G., Zeng G., He Z., Gou X., He X., Liu M. (2024). Cellular Automata-Based Experimental Study on the Evolution of Corrosion Damage in Bridge Cable Steel Wire. Buildings.

[110] Toribio J., Ayaso F.-J., Rodríguez R. (2023). Intercolonial Microdamage and Cracking Micromechanisms during Wire Drawing of Pearlitic Steel. Materials.

[111] Xue S., Shen R., Chen W., Miao R. (2020). Corrosion fatigue failure analysis and service life prediction of high strength steel wire. Eng. Fail. Anal..

[112] Cai P., Hu C., Zhu X., Zhang S., Xiao G., Chen J., Xue H., Zhao J., Misra R.D.K., Wu K. (2026). Degradation behavior and prediction of corrosion fatigue life of Zn-10Al-RE coated ultra-high strength steel wire in extreme corrosion environment. Corros. Sci..

[113] Duan Y.F., Wu S.K., Zhang H.M., Zhang R., Yun C.B., Cheng J.J.R., Wang S.M. (2024). Development of corrosion-fatigue analysis method of cable hangers in tied-arch bridges considering train-bridge interaction. Eng. Struct..

[114] Guo J., Liu J., Zhong Y. (2025). Assessment for the service life of cable wire under corrosion fatigue coupling effect in extreme marine environment. Eng. Fract. Mech..

[115] Wang Q., Yao G., He X., Yang S., Hou M., Zhang T. (2026). Time-dependent corrosion-fatigue reliability of parallel-wire stay cables based on weibull life modeling and system load redistribution. Constr. Build. Mater..

[116] Yao G., Zeng G., He X., Wang Q., Wu Y., Jiang E., Zhu Y., Wang B. (2025). Study on corrosion damage mechanisms and fatigue life of corroded steel wires in suspension bridge cable strands. Case Stud. Constr. Mater..

[117] Xue S., Shen R., Xue H., Zhu X., Wu Q., Zhang S. (2021). Failure analysis of high-strength steel wire under random corrosion. Structures.

