# Supplementary material for: Zn-Based Coatings and Durability of High-Strength Pearlitic Bridge Wires: A Critical Evidence Appraisal
Source: Materials (Basel). 2026 Nov 4;19(21):4692. doi: 10.3390/ma19214692 (PMC13765254; doi:10.3390/ma19214692)
Supplement: Supplementary file 1 [file materials-19-04692-s001.zip › materials-4629753-supplementary.pdf]

Supplementary Evidence Appraisal and Source Records

Table S1. Sixty-one source families selected for detailed comparison and supporting evidence on coating, hydrogen, fatigue, contact, models and assemblies. C1: coating attribution; C2: hydrogen and mechanical histories; C3: inherited damage versus continued exposure. Linked reports or reused results are one experimental family unless independent specimens are established. NR means not reported.

Study family	Comparison / role	Source notes
Gelfi et al. (2017) [21]	C1 — manufacturing state	S1-01
Zhang et al. (2023) [8]	Qualitative desorption; preparation/loading-control case	S1-02
Zhu et al. (2022) [14]	C1 — coating-process pair	S1-03
Zhao et al. (2023) [20]	C1 — salt-spray product pair	S1-04
Hirakami et al. (2016) [61]	C2 — aging and hydrogen response	S1-05
Enos and Scully (2002) [54]	C2 — hydrogen and crack initiation	S1-06
Jia et al. (2025) [55]	C2 — hydrogen-supply histories	S1-07
Huang et al. (2025) [56]	C2 — deformation and retention	S1-08
Nakamura and Suzumura (2009) [51]	C2 / C3 — hydrogen and wet/dry loading	S1-09
Elices et al. (2004) [60]	C2 — stabilization and residual stress	S1-10
Liu et al. (2024) [29]	C3 — pre-corrosion and fracture origins	S1-11
Yang et al. (2026) [46]	C3 — remaining-area geometry	S1-12
Wang et al. (2020) [81]	C3 — notches in air and solution	S1-13
Miyachi et al. (2023) [80]	C3 — contrary fatigue ordering	S1-14
Li et al. (2025) [85]	C3 / C1 — coating endpoint under loading	S1-15
Deng and Deng (2022) [79]	C3 — pre-corrosion, polishing, dry fatigue	S1-21
Yang et al. (2012) [40]	C1 — static-stress acid-rain exposure	S1-26
Gu et al. (2026) [18]	C1 — finished Zn–Al–Mg wire, salt spray	S1-30
Wan et al. (2022) [43]	C1 / C3 — galvanized wire section and tensile loss	S1-31
Cao et al. (2003) [52]	C2 / C3 — section loss and fracture morphology	S1-32
Xue and Shen (2020) [111]	C3 — salt spray then dry fatigue	S1-33
Cai et al. (2026) [112]	C3 — alloy-coated wire, salt spray then fatigue	S1-34
Montalvo et al. (2024) [87]	Supporting — contact	S1-16
Zhang et al. (2025) [93]	Supporting — sustained load	S1-17
Ding et al. (2026) [102]	Supporting — strand model	S1-18
Lan et al. (2018) [101]	Supporting — successive wire failures	S1-19
Li et al. (2023) [97]	Supporting — saddle contact	S1-20
Jarwali et al. (2014) [35]	Supporting — 19-wire strand wetting	S1-22
Tomatsu et al. (2025) [62]	Supporting — notched miniature specimen orientation	S1-23
Wang et al. (2024) [108]	Supporting — laboratory wire-contact wear	S1-24
Zhou et al. (2024) [109]	Supporting — accelerated exposure of already-corroded galvanized wire	S1-25
Zhao et al. (2023) [103]	Supporting — prior coating-to-suspender model	S1-27
Xue and Shen (2024) [100]	Supporting — one-cable wire-break sequence	S1-28
He et al. (2023) [107]	Supporting — laboratory corrosion-state inspection	S1-29
Jiang et al. (2024) [27]	Supporting — resistance/gravimetry method	S1-35
Recio et al. (2011) [53]	Supporting — alkaline-medium hydrogen exposure	S1-36
Chehrazi et al. (2023) [92]	Supporting — dry saddle-contact fretting	S1-37
Duan et al. (2024) [113]	Supporting — prior hanger-stage model	S1-38
Guo et al. (2025) [114]	Supporting — prior galvanized-wire/cable model	S1-39
Li et al. (2007) [37]	C1 — loaded-wire rust detection	S1-40
Zhang et al. (2024) [28]	C1 — imposed-current mass loss	S1-41
An et al. (2026) [99]	Supporting — prior pit-to-cable model	S1-42
Lan et al. (2026) [82]	C3 — potential/life counterexample	S1-43
Liu et al. (2020) [91]	C3 — interwire corrosion-fretting	S1-44
Wang et al. (2026) [115]	Supporting — prior cable reliability model	S1-45
Xue and Shen (2025) [83]	C3 — prefatigue then seawater fatigue	S1-46
Guo et al. (2026) [38]	C1 / C3 — paired coating-to-fatigue series	S1-47
Yan et al. (2024) [42]	C1 — finished-wire pit/section–tensile series	S1-48
Gao et al. (2023) [44]	C1 — field-origin geometry contrast	S1-49

Study family	Comparison / role	Source notes
Lan et al. (2024) [74]; Bing et al. (2025) [75]	C3 — linked exposed-window fatigue and model reports	S1-50
Wang et al. (2020) [69]	C3 — machined-notch saline/air contrast	S1-51
Yang et al. (2026) [76]	C3 — independent exposed-spot model endpoint check	S1-52
Wang et al. (2023) [89]	C3 — local saddle-contact zinc loss	S1-53
Chong et al. (2024) [90]	C3 — wire/saddle versus wire/wire contact	S1-54
Yao et al. (2025) [116]	C1 / C3 — mixed-coating stage and fatigue report	S1-55
Xue et al. (2021) [117]	C1 / C3 — corroded-wire geometry and tensile response	S1-56
Yuan et al. (2021) [39]	C1 — Galfan corrosion and local iron products	S1-57
Xu et al. (2026) [84]	C3 — exposed-notch environmental fatigue contrast	S1-58
Hu et al. (2023) [19]	C1 — five coating products and prepared-notch products	S1-59
Yuan et al. (2025) [98]	Supporting — wire-strength model versus proposed cable workflow	S1-60
Doshida and Takai (2014) [57]	C2 — selective desorption with thermal control	S1-61

Source notes for Table S1

S1-01. Gelfi et al. (2017) [21] — C1

DOI: 10.3390/ma10030264

Source locations: PDF pp. 3 and 8–10, including the same-batch spray-on/off comparison and coating micrographs.

Test conditions: C82D steel; air–water spray cooling was switched on or off within steel Batch 9 along the galvanize-before-drawing route. SEM observations of the coating were made before drawing. Endpoints were coating cracks and torsion response.

Observation: Cracks existed before torsion. With spray cooling, cracks extended through the coating; without spray cooling, they remained within the delta layer.

Limitations: The galvanize-before-drawing route differs from coating finished bridge wire. This is a manufacturing-state comparison, without an atmospheric corrosion-onset or service-life endpoint. The source also describes a separate Steel 1/2 example involving 7 mm rod drawn to 4.5 mm; those dimensions are not assigned to Batch 9.

S1-02. Zhang et al. (2023) [8] — qualitative desorption and preparation/loading controls

DOI: 10.3390/coatings13081339

Source locations: Methods and material compositions; FIP results on PDF pp. 4 and 6–7, Table 2 and Figure 5; electrochemical and TDS results in Sections 3.2–3.4 and Figure 17.

Test conditions: Nominal 6 mm, 2060 MPa-grade ZnAl and ZnAlMg products with approximately 50 µm coatings and different wire-rod compositions. Stripped states underwent HCl removal, water rinsing and 2000-mesh polishing. Exposure used 20 wt.% NH_4SCN at 50 °C. FIP loads were 70% and 80% of $F_{max} = A\sigma_{grade}$, where A is area and σ_{grade} is the strength grade; they were not separately measured state-specific maxima. Desorption specimens were prepared separately and exposed for 24 h.

Observation: Coated and stripped specimens show different source-reported desorption signals before and after exposure; ZnS and Al_2O_3 were identified during exposure. These are qualitative surface-state observations. The FIP preparation and loading definitions provide a control-design case; no fracture-time effect estimate is assigned.

Interpretation limits: Stripping and polishing change surface geometry and chemistry, while the products differ in wire-rod composition. Sixteen FIP specimens are reported overall, but allocation to the eight conditions, plotted aggregation and error-bar definitions are unresolved. The state-specific force/area basis is also unresolved. Desorption signals are not integrated hydrogen contents and do not identify hydrogen in a particular fractured specimen. The electrochemical method gives a saturated-calomel reference and graphite counter electrode without the exposed length, masking boundary and state-specific area needed to partition Zn and Fe reactions. This evidence supports neither a quantitative coating effect nor a composition or atmospheric-life ranking.

S1-03. Zhu et al. (2022) [14] — C1

DOI: 10.3390/met12081257

Source locations: Methods, tensile procedure and Table 3; Table 5 on PDF p. 5; Section 3.3 and strength values on PDF pp. 8–9; processing comparison in the Conclusions.

Test conditions: 6.0 mm high-strength bridge wire (nominal strength grade not reported); nominal Zn–10 wt.% Al–0.08 wt.% mischmetal precursor ingots; 450 or 475 °C immersion for 45 s, followed by water cooling; three tensile specimens per state. The nominal precursor formulation is not a measurement of the finished-coating composition.

Observation: For 450-45-W and 475-45-W, coating thicknesses were 37.94 and 53.29 μm and mean tensile strengths were 2031 and 1988.3 MPa, respectively. SEM/EDS showed dispersed Al-rich regions at 450 °C and lamellar, locally clustered Al-rich regions with uneven reaction layers at 475 °C. Temperature, coating development and substrate thermal history varied together.

Interpretation limits: The 42.7 MPa difference uses three tensile specimens per state. The error-bar statistic and numerical dispersion are undefined, and no difference test is given. Visually separated error bars do not establish significance. Quantified phase fractions and specimen-by-specimen morphology–strength linkage are absent. The comparison establishes neither a single-factor strength effect nor a corrosion-onset or sacrificial-capacity threshold.

S1-04. Zhao et al. (2023) [20] — C1

DOI: 10.3390/ma16020708

Source locations: Methods, sampling Table 1, Section 3.1 and the paragraphs reporting the initial coating thicknesses and first visible reddish-brown corrosion products.

Test conditions: Neutral salt spray on galvanized and Galfan products, 5.34 and 5.25 mm in diameter, with measured tensile strengths of 1895 and 1926 MPa and coating mass per area of 336 and 337 g/m², respectively; nominal wire strength grades were not reported. Source coating labels list Zn ≥99 wt.% for galvanized and Zn ≤95 wt.%, Al ≤5 wt.% for Galfan. The source's calculated thickness values cannot be reconstructed from its tabulated masses by an ordinary mass–density conversion and are not used as a thickness-matched control in this review. Visible steel-corrosion products were assessed at sampling points.

Observation: The main results report reddish-brown corrosion products at 384 and 1016 h, respectively.

Limitations: The source conclusion gives 386 h, whereas the main results and sampling sequence use 384 h; the main-results value is retained and the discrepancy recorded. This is a visible, product-specific onset. Coating mass, composition and substrate effects are not isolated, and the comparison is not a lifetime multiplier.

S1-05. Hirakami et al. (2016) [61] — C2

DOI: 10.2355/isijinternational.ISIJINT-2015-735

Source locations: PDF pp. 2–4, Table 2, Figure 1 and Section 3.2; charging, transfer and thermal-desorption methods.

Test conditions: 5 mm SWRS82B wire: as-drawn versus aging at 100 or 300 °C for 10 min. Notched mechanical tests used precharging and continued charging; the TDA heating rate was 100 °C/h.

Observation: The respective strengths were 1943, 2029 and 2038 MPa, and low-temperature saturated hydrogen contents were 4.0, 2.5 and 1.0 mass ppm. Aging improved the charged-to-uncharged fracture-stress ratios.

Limitations: The English 2016 and Japanese 2015 publications form one experimental family, not independent corroboration. The results establish a processing-state association, not a universal hydrogen threshold or proof that all hydrogen in a TDA peak is active in fracture.

S1-06. Enos and Scully (2002) [54] — C2

DOI: 10.1007/s11661-002-0217-z

Source locations: Materials, notched CERT and permeation methods; fracture-initiation results and critical-strain interpretation on pp. 7–8 and 13.

Test conditions: Drawn pearlitic prestressing material with blunt or sharp notches and precracks; controlled cathodic hydrogen supply. Breaking response was measured, hydrogen was derived from permeation, and strain was calculated by finite elements.

Observation: Higher hydrogen availability accompanied lower fracture-initiation stress for the specified geometry.

Limitations: Critical local strain was calculated, not measured directly at the notch. The results concern susceptibility of a notched substrate, not atmospheric hydrogen flux into a finished coated wire or a universal threshold.

S1-07. Jia et al. (2025) [55] — C2

DOI: 10.1016/j.ijhydene.2025.151226

Source locations: Methods on pp. 2–3; Figure 4 on p. 5; Figures 5–8 on pp. 6–8; Discussion on p. 11.

Test conditions: Source wire of 7 mm diameter and 2100 MPa grade; tensile parallel section of 3 mm diameter. Ex-situ, in-situ and combined hydrogen histories; precharging at 1 mA/cm²; testing at 0.01 mm/min in 0.2 mol/L NaOH + 0.5 g/L thiourea at 298 K; three tensile tests per condition.

Observation: Within each parameter series, elongation loss and fracture features depended on the hydrogen-supply history.

Limitations: The condition assigned to 57% differs between the p. 5 prose and Figure 4a, so condition-specific numbers are omitted. Jia reported tensile and TDS tests, not APT; its independently prepared plate specimens do not identify fracture-active hydrogen in the tensile tests. The separate APT study is Huang [56].

S1-08. Huang et al. (2025) [56] — C2

DOI: 10.1016/j.actamat.2025.121231

Source locations: Main article Sections 2.3–2.5 on p. 3; Section 3.2 and Figure 3 on p. 6; Section 3.3 and Figure 4 on p. 7; publisher Supplementary Table S2 for the individual SSRT observations; separate APT and size-model discussion.

Test conditions: Unstrained and highly strained pearlite. Tensile and separately prepared TDS specimens were hydrogen-charged for 24 h at 50 mA/cm² in 0.1 M NaOH + 1 g/L NH4SCN; tensile gauge lengths were 50 and 10 mm, with three measurements per condition. TDS used separate 10 × 5 × 1 mm cuboids for the 0, 2 or 12 h room-temperature retention comparison; the highly strained specimens' source Figure 3D/E series used ramps of 100, 150 and 200 °C/h to 500 °C for Kissinger detrapping-energy estimation. Deuterium APT tips followed a different, short 2.2 V charging and cryotransfer protocol (Table S1M).

Observation: Publisher Supplementary Table S2 lists three individual elongations for each uncharged, H-charged and 12 h-desorbed tensile condition in both material states. Main-text Table 2 gives their sample means, standard deviations and ranges. Source-reported charged-state relative losses of 22.1% and 14.4% use $100(\varepsilon u - \varepsilon H)/\varepsilon u$, with εu equal to each state's own uncharged mean. Mean elongation increased after 12 h desorption. Separately prepared TDS specimens retained a higher-temperature release feature, and separate APT tips showed local deuterium retention.

Interpretation limits: The two tensile states have different processing histories and gauge lengths. Source Figure 4 shows ranges; Table 2 sample SDs are calculated from the three rounded individual values per condition in publisher Supplementary Table S2. The source's Error (%) field is undefined and is not treated as SD. Its desorbed-loss percentages do not consistently reconstruct from the displayed triplets and are not used. Separate TDS and APT specimens cannot identify hydrogen active in tensile fracture; within-state normalization does not establish an absolute cross-material susceptibility ranking.

S1-09. Nakamura and Suzumura (2009) [51] — C2 / C3

DOI: 10.1016/j.jcsr.2008.03.022

Source locations: Section 2.3 and Figures 10–11 on PDF pp. 3–4; Sections 3.1–3.2 and Figures 12–16 on PDF pp. 4–5 (printed pp. 272–273).

Test conditions: Hydrogen tests on corrosion-level-2 wires with and without tension; separate dry/wet fatigue tests on levels 2 and 3. Grip spacing was 100 mm and frequency 64 Hz. Dry relative humidity was below 50%; wet tests used gauze enclosed in a silicone tube.

Observation: Diffusible hydrogen was below 0.2 ppm in the corrosion-level-2 tension comparison. Within the reported corrosion levels, wet fatigue strength was lower than dry fatigue strength.

Limitations: Hydrogen was not measured in the fatigue specimens during loading. The 0.7 ppm criterion is source-specific, and no hydrogen value is transferred to level 3. Separate finished-wire test sets do not establish universal exclusion of hydrogen damage or quantify the dissolution/hydrogen partition.

S1-10. Elices et al. (2004) [60] — C2

DOI: 10.1007/BF02481676

Source locations: Mechanical-property table and XRD/FIP methods and results on PDF pp. 2–4; specimen-count narrative on printed p. 307 and Tables 3–4 on p. 308.

Test conditions: 5.20 mm drawn and stabilized wire; loading at 80% of each state's maximum force. Longitudinal stress in surface ferrite was measured by Cr Kα XRD in Ω mode using $\sin^2\psi$. Mixture-rule macro-residual-stress estimates are distinct from this measured phase stress.

Observation: Strength changed from 1940 to 1850 MPa, surface-ferrite residual stress from 260 ± 20 to 30 ± 5 MPa, and mean FIP fracture time from 2.0 to 4.2 h.

Limitations: The source narrative states six specimens per state; Table 3 lists eight drawn-wire rupture times at 0.80 Fm, whereas Table 4 lists six stabilized-wire times. The source-reported means 2.0 and 4.2 h are retained without assigning a reconciled sample count. The counts 41 and 82 refer to residual-stress measurements, not established independent wire counts. Strength, stress and test force changed together; neither a residual-stress-only mechanism nor a common quantitative susceptibility ranking with other experiments is established.

S1-11. Liu et al. (2024) [29] — C3

DOI: 10.3390/ma17081724

Source locations: Salt-spray, cleaning and dry-loading methods, and Figures 10 and 12 with their captions, including the reported pit and fracture-origin observations.

Test conditions: 5.0 mm galvanized and 5.4 mm ZnAl products with different coating inventories; pre-corrosion followed by cleaning and dry cycling.

Observation: Pit-associated crack origins and the exposure sequence remain informative. The uncorroded datum at 2×10^6 cycles is a runout.

Limitations: The 360 MPa amplitude/range and force–area descriptions remain unresolved, so the stress-labelled curve is excluded from quantitative matching. Geometry–fracture association does not provide a measured initiation fraction or a coating-composition-only fatigue effect.

S1-12. Yang et al. (2026) [46] — C3

DOI: 10.3390/ma19102099

Source locations: Sections 2.2–2.3, Tables 2–4 and Figure 7 on PDF pp. 3–5 and 11.

Test conditions: 7 mm, 1770 MPa galvanized wire; electrochemical pre-corrosion in 5 wt.% NaCl; loading between 437 and 797 MPa at 10 Hz. There were 42 wires in seven groups, with three fatigue wires per group.

Observation: Controls A4–A6 and pre-corroded B4–B6 all ran out at 2×10^6 cycles; B4–B6 had mass losses of 2.73%, 2.79% and 2.76%. These are right-censored outcomes. Scanned profiles provided remaining-area geometry. The loading extrema give $\Delta\sigma = 360$ MPa, $\sigma a = 180$ MPa and $R \approx 0.548$.

Interpretation limits: One wire was scanned per group; profiles were sampled every 0.2 mm over 200 mm. Sampling interval is not scanning accuracy, and filtering may suppress narrow features. Series-connected corrosion does not establish independent process runs. Cycling atmosphere and stress-area convention are not established. Source Figure 7 labels D6 $\eta = 9.46\%$, whereas Tables 2 and 3 give 9.49%. These results support within-series damage comparisons, not a universal threshold or a matched environmental comparison across studies.

S1-13. Wang et al. (2020) [81] — C3

DOI: 10.3390/app10072293

Source locations: Groups A and D, loading in Table 3 and cycles in Table 4 on pp. 10–12; notch geometry and local solution-supply methods.

Test conditions: Identically notched 7 mm wire; air or 0.5/3.5 wt.% NaCl at pH 7; 0.5 Hz and R = 0.44; reported stress ranges of 360–720 MPa.

Observation: Reported lives in both solutions were shorter than in air, while the 3.5 wt.% entries were longer than the 0.5 wt.% entries.

Limitations: Per-condition replication and aggregation remain unresolved, and oxygen was not measured. Specimen identifiers do not establish n = 1. These are descriptive within-study orderings, without an effect-size ratio, concentration law or partition of mechanisms.

S1-14. Miyachi et al. (2023) [80] — C3

DOI: 10.3233/BRS-230214

Source locations: Publisher proof PDF, Sections 4.1–4.2, Figure 13 and Table 8 on PDF pp. 9–11.

Test conditions: Corrosion levels 1 and 2 and new wire; minimum stress 500 MPa, stress range 600–700 MPa and frequency 10 Hz. The small series used a stopping rule of 2×10^6 cycles.

Observation: In its tested series, the source reports fatigue strength of corroded wire exceeding that of new wire.

Limitations: Sampling variation and limited corrosion are proposed explanations, neither demonstrated. The table comparison remains descriptive. The contrary observation is retained without claiming beneficial corrosion or an improvement in the population.

S1-15. Li et al. (2025) [85] — C3 / C1

DOI: 10.1016/j.conbuildmat.2025.142613

Source locations: Methods, Table 3 and Figures 4 and 6 on PDF pp. 2–5; Section 3.7 and Figure 14 on p. 8; values and Figures 15–17 on p. 9.

Test conditions: 7 mm, 1670 MPa galvanized wire; exposed window 0.5 cm^2; neutral 3.5% NaCl at approximately 25 °C; imposed anodic current density 20 mA/cm^2. Stress ranges were 180, 360 and 540 MPa, with $\sigma_{max} = 0.6\sigma_u$.

Observation: The fitted potential-segment D/E intersection gave 10,241 s without load; 9577, 6612 and 3400 s at 10 Hz; and 4193, 5898 and 6612 s at 1, 5 and 10 Hz, respectively, for the 360 MPa range.

Limitations: Endpoint aggregation and dispersion are unknown; two curves at one condition do not establish uncertainty for all rows. The mass basis of 3.5% is unstated, and the source's $0.3/0.33\sigma_u$ inconsistency is not used. The operational electrochemical endpoint is not complete zinc removal or fatigue failure and cannot be converted to atmospheric performance.

S1-16. Montalvo et al. (2024) [87] — supporting contact evidence

DOI: 10.1016/j.wear.2024.205383

Source locations: HAL v2 repository PDF pp. 3–5, Sections 2.1–2.2, Eqs. (4)–(6) and Table 2; pp. 10–11, Section 3.4 and Figure 16; p. 10, Section 3.3.2 and Figure 15; pp. 13–14, Section 3.6. Selected numeric records are in Table S1N.

Test conditions: 4.85 mm Zn–5%Al-coated wire, approximately 50 μm coating, or inhibited-HCl-stripped product; 30° crossed contact, P = 1400 N, ambient air and no grease. Table S1N uses σ_m = 320 MPa and σ_a = 160 MPa. δ^* is a rig-dependent effective actuator amplitude after fatigue-elongation compensation, not local slip.

Observation: The source reports a partial/gross-slip transition coefficient near 0.8 in fretting fatigue and near 0.9 in separate 10^4-cycle plain fretting. Gross-slip $Q^*/P \approx 0.7$ in the latter is interpreted as μ_{GS}. Source-listed scar areas and cycles retain different effective displacements and/or tangential forces; they are not coating-only effects. A crack was observed while zinc remained elsewhere.

Limitations: Much of the bright-wire series was previously published and reused, so the rows are not all new independent evidence. This offshore-rope contact geometry does not directly qualify a bridge detail, and force alone does not establish local contact pressure. Scar ellipse area is not a measured pressure field; the effective actuator amplitude, local slip and friction quantities have different definitions.

S1-17. Zhang et al. (2025) [93] — supporting sustained-load evidence

DOI: 10.1016/j.ijhydene.2024.11.408

Source locations: Methods on PDF p. 2, Figure 4 on p. 4 and Discussion on p. 8; separate 2 h TDA and constant-load FIP schedules.

Test conditions: 7 mm, 1770 MPa acid-stripped wire; 20 wt.% NH_4SCN at 50 °C; loading at 70% F_{max} with varied imposed current. Separate 2 cm TDA specimens were treated for 2 h.

Observation: The fracture endpoint varied nonmonotonically with anodic current; hydrogen release in the separate specimens varied with polarization.

Limitations: Mechanical and TDA treatments, times and currents were not paired, preventing one-to-one identification of hydrogen associated with fracture. These are accelerated results for a stripped substrate, not a service coating ranking or a wire-to-cable conversion.

S1-18. Ding et al. (2026) [102] — supporting assembly evidence

DOI: 10.3390/ma19040646

Source locations: Methods for salt-spray morphology and the idealized strand model, the model boundary conditions, and the results relating pit position to calculated stress concentration.

Test conditions: Galvanized 1 × 7 strand; salt-spray morphology and idealized elliptical pits. The example uses 44.8 kN tension and 0.6° rotation; the model excludes interwire slip.

Observation: At fixed pit geometry, calculated stress concentration increased as the pit approached the restrained anchorage.

Limitations: The model uses idealized geometry and restricted boundary conditions. A measured surface does not make simulated stress a measured field. This configuration-specific strand model does not validate the complete history from coating degradation to cable response.

S1-19. Lan et al. (2018) [101] — supporting assembly evidence

DOI: 10.1016/j.ijfatigue.2018.05.020

Source locations: Sections 3–4 on PDF pp. 5–7, including equal load sharing, identical stiffness and corrosion, independent initial fatigue lives and progressive wire failure.

Test conditions: Lan et al. represented a parallel-wire stay by 300 wires with an initial nominal stress range of 335 MPa; corrosion level and stiffness were common to all wires, loads shared equally, friction ignored, and initial fatigue lives independently distributed.

Observation: The model updates survivor stress as wires fail. Its equal-sharing calculation and ±1-break input sensitivity are set out in Supplementary Note S3L.

Interpretation limits: The stress update is model arithmetic, not a measured cable stress. Common corrosion, independent initial lives and equal sharing exclude spatially correlated damage and frictional force recovery; no empirical assembly validation or cable-life factor follows.

S1-20. Li et al. (2023) [97] — supporting assembly evidence

DOI: 10.3390/coatings13030646

Source locations: Methods, local model in Section 3 and Figure 10, including the figure geometry and labels.

Model conditions: 5.65 mm, 2200 MPa wire; a local five-wire, two-layer U-groove model at the saddle outlet. The source saddle geometry uses an 18 m centreline curvature radius and a 62.5 by 64 mm strand groove. A first-stage model imposes 40.9 MPa equivalent pressure at the strand top; its calculated 44.7 MPa interlayer compression is applied to the second-stage five-wire model. These pressures are model inputs or outputs, not measured local contact pressures.

Observation: The model places the critical location at the bottom wire near the groove outlet. Parameters were calibrated to a specific single-wire test.

Limitations: Calibration, fitting and the saddle prediction do not provide independent component validation. The results concern only local mechanical demand in the specified configuration.

S1-21. Deng and Deng (2022) [79] — C3

DOI: 10.3390/coatings12101475

Source locations: Recovered suspender-wire material, acid-salt-spray/dry-fatigue methods, Table A1 and Figure 15. Figure 15 repeats an incorrect 10-day label for polished curves; the present Figure 7 uses all 30 specimen-level entries from Table A1.

Test conditions: Intact galvanized 7 mm, nominal 1670 MPa wire from an 11-year suspender; acid salt spray at 35 °C for 10, 20, 40, 60 or 90 days; subsequently dry fatigue at 25 Hz. At each duration three unpolished and three separately prepared polished wires were tested at an approximately 360 MPa nominal stress range. The source inconsistently uses amplitude and range, but defines ΔS as nominal range and gives maximum stress and $R \approx 0.52$ consistent with that assignment to rounding.

Observation: Table A1 contains 30 individual cycles. At all five durations the polished group has a higher mean life than the corresponding unpolished group, by 34–60%; Figure 7 shows each value with the within-group mean and sample SD.

Limitations: Specimens were not paired, and polishing changes surface geometry and potentially net steel section. The cycle difference cannot be assigned solely to pit removal or coating state; differences in specimen history, area basis and source rounding preclude stress-matched comparison with other studies. This is a wire experiment, not cable-life validation.

S1-22. Jarwali et al. (2014) [35] — supporting strand-scale evidence

DOI: 10.2208/jscejseee.70.319

Source locations: J-STAGE article, Japanese Methods and Table 1, Figures 4–6 and English abstract.

Test conditions: Each strand comprised 19 parallel 5 mm, 1665 MPa wires; Zn and Zn–10Al coatings were compared. After 0.01% NaCl application at 40 °C, conditions were 60% RH, 100% RH or continuously wet gauze. One independent strand per coating, environment and sampling time was removed at 30, 90 and 150 days; five wires sampled within a strand are subsamples, not independent strands.

Observation: At 60% RH surfaces remained largely intact through 150 days. At 100% RH attack remained in the coating with no visible iron rust. Continuous wetting caused more marked damage; red rust was visible especially on Zn-coated strands at 150 days, while Zn–Al showed lower reported mass loss under persistent wetting.

Limitations: The study reports coating and visible-corrosion response for specific strand/exposure combinations, with one independent strand at each condition and time. It gives no fatigue, wire-rupture or cable-capacity endpoint and cannot define a universal humidity threshold.

S1-23. Tomatsu et al. (2025) [62] — supporting orientation experiment

DOI: 10.2355/isijinternational.ISIJINT-2024-294

Source locations: Official J-STAGE full PDF, Sections 2.2–3.3 and 4, Table 2 and Figures 4–8, 14–19 (PDF pp. 3–9; printed pp. 157–163).

Test conditions: Drawn and annealed 5 mm pearlitic wire machined into notched miniature coupons with approximately 0.4 × 0.2 mm net section; in-situ plasma hydrogen charging during SEM testing. The tensile direction was perpendicular to the drawing direction for types I/II and parallel for type III. Air controls were one specimen per orientation; charged groups were three, three and four specimens for the reported orientations.

Observation: Charged transverse orientations reached lower fracture stresses than their single air controls; charged axial coupons sustained higher stresses than charged transverse coupons. The specimen geometry and fracture images indicate that the nominal type-I/II fracture planes contain the drawing direction, with local deviations in the fracture paths. One of the three fractured type-III specimens showed a longitudinal stationary subcrack; the authors interpreted it as opening under notch-induced transverse tension.

Limitations: The single air control in each orientation restricts quantitative hydrogen-effect inference. The miniature notch and plasma charge do not reproduce finished coated-wire corrosion or a bridge saddle/anchorage loading limit. The longitudinal subcrack does not define a common final-fracture plane. The source does not determine crack-plane angles under contact compression or frictional shear; the service-plane discussion in Section 4.4 is a conditional mechanics interpretation.

S1-24. Wang et al. (2024) [108] — supporting laboratory contact experiment

DOI: 10.1007/s40544-023-0830-y

Source locations: Experimental methods on pp. 3–4 and Discussion and Conclusions on pp. 18–19.

Test conditions: Same-batch 1.4 mm hot-dip galvanized wire; 2500-mesh polishing before contact testing; bending tribo-corrosion-fatigue rig at 6 Hz, up to 2 × 10^5 cycles, in 3.5 wt.% NaCl or ultrapure water.

Observation: The article reports contact-wear and electrochemical responses under its specified cyclic loads and aqueous media. Its 150 wear-width locations are measurements within tests, not 150 independently treated wires.

Limitations: Independent wire replicate counts are not specified. The reported bulk-composition row is internally implausible and is not reused here. The experiment does not measure fatigue fracture or cable capacity, and polished 1.4 mm contact wires cannot validate a full-size bridge-wire or cable life effect.

S1-25. Zhou et al. (2024) [109] — supporting accelerated finished-wire exposure

DOI: 10.3390/buildings14113354

Source locations: Sections 2.1.3 and 2.2.1, Figures 4–5 and Table 4.

Test conditions: Outer-ring 7 mm, 1770 MPa galvanized wires from the anchorage region of one retired coastal stay cable. Three different wires per state: naturally corroded baseline, and separate groups after one, three or five 24 h imposed-current wet periods alternating with 24 h drying. Wet periods used 5 wt.% NaCl at 35 °C and >75% RH with 2 mA/cm^2 applied anodic current.

Observation: The baseline photographs already show local red rust on two wires. Images of the separate accelerated groups show greater local steel exposure and rust with more imposed-current wet/dry cycles. Table 4 reports group-mean tensile strengths of 1745.13 MPa for the naturally corroded group and 1683.52 MPa for the five-cycle group, with three wires per group; these are descriptive distinct-wire groups.

Limitations: Initial coating thickness and phase distribution were not measured for the contrasted wires; the baseline was already corroded, and three wires per state do not represent three independent exposure runs. The images do not track one

wire through time, identify first steel exposure from an intact coating, isolate a coating-composition effect, or calibrate atmospheric/cable service life.

S1-26. Yang et al. (2012) [40] — C1

DOI: 10.1002/maco.201005921

Test conditions: A 5 mm galvanized bridge wire was polished in its test region and exposed under static stresses of 0–1100 MPa to 20-fold simulated acid rain at pH 4.5. The spray programme ran for 61 cycles of 12 h spray plus 12 h rest at 35 ± 2 °C.

Source locations: PDF pp. 2–5, Methods, Table 1, Table 3 and Figures 5, 6 and 8.

Observation: Endpoint mass loss rose across the imposed stress states, and iron-bearing corrosion products were identified at the highest stress. The reported corrosion current combines reactions on the exposed surface.

Limitations: Three cut segments from one exposed wire are spatial subsamples, not three independent wires. Independent-wire replication, first steel exposure, local Zn/Fe current and remaining steel area were not established; these accelerated results do not define service life.

S1-27. Zhao et al. (2023) [103] — supporting engineering calculation

DOI: 10.3390/ma16010290

Model conditions: A published Galfan-coating loss curve and a conversion based on 25 μm Galfan-coated sheet exposure feed subsequent steel corrosion, maximum-pit statistics, Forman crack growth and traffic-loaded short-suspender calculations.

Source locations: Europe PMC XML, Sections 3.2–3.3 and 5.2–5.3, Tables 2 and 4. The coating-loss input traces to Yuan et al., DOI 10.1016/j.conbuildmat.2021.125534, and a 25 μm coated-sheet-to-wire conversion; these are imported model inputs, not a measured loss history for the modeled suspender.

Observation: The study checks bridge modal frequency and deflection against measurements. Its 150 pit-coefficient draws per condition are model realizations rather than 150 tested wires.

Limitations: Coating-loss history, crack-growth history and predicted suspender life were not independently verified. The modeled years and intervals depend on the imported corrosion inputs and transfer assumptions; they are not observed service lives.

S1-28. Xue and Shen (2024) [100] — supporting assembly experiment

DOI: 10.1061/JBENF2.BEENG-6621

Test conditions: One laboratory cable of 121 wires was subjected to combined tension, end bending and external sponge wetting. The solution composition and Zn-coating state were not specified in the examined methods.

Source locations: PDF pp. 10–16, experimental preparation, Figures 23–29 and 31.

Observation: The first to ninth observed wire breaks occurred between 1,436,936 and 2,300,198 cycles, when the test stopped. A boundary stiffness was adjusted to match the measured wire stress before comparing the model with this same cable.

Limitations: Nine breaks in one cable are correlated observations, not nine cable replicates. The experiment did not measure ultimate cable capacity, remaining cable force after each break or service life; the same-series model comparison is not external validation.

S1-29. He et al. (2023) [107] — supporting inspection experiment

DOI: 10.1007/s44251-023-00018-1

Test conditions: Four visually classified 7 mm galvanized wires recovered from a bridge cable were measured individually and placed at different depths in laboratory wire bundles for 80 kHz electromagnetic response tests.

Source locations: PDF pp. 5–11, Sections 3–4, Figures 8–15 and Conclusions.

Observation: First-layer visual states were distinguishable; second-layer corrosion presence remained detectable without clear state separation, and third-layer severity was unresolved.

Limitations: One wire per visual class and no matched coating or remaining-section measurement preclude a calibrated coating-loss estimate. Bundle response was not an in-service cable-capacity or fatigue-life measurement.

S1-30. Gu et al. (2026) [18] — C1 — finished-wire coating evolution

DOI: 10.1016/j.jelechem.2025.119710

Source locations: PDF pp. 2–5, material and neutral-salt-spray methods, product and coating cross-sections; electrochemical methods and results.

Test conditions: Industrial 5.95 ± 0.02 mm, 2060 MPa wire with about 60 µm hot-dip Zn–Al–Mg coating; 5 wt.% NaCl spray at 35 ± 2 °C. Electrochemical repeats were reported, but independent salt-spray replication by exposure time was not established.

Observation: Coating products and local chloride penetration evolved through 1200 h; Zn–Al layered double hydroxide was reported from 960 h. The electrochemical response concerns the tested surface state.

Limitations: No measured first local steel-exposure time, matched thickness series or wire-strength endpoint is supplied. Its product evolution cannot be merged with another spray protocol into a coating-composition protection law.

S1-31. Wan et al. (2022) [43] — C1 / C3 — section and tensile loss

DOI: 10.1016/j.conbuildmat.2022.128387

Source locations: PDF pp. 2–3 and 9–11, Table 1, 3D morphology and tensile results.

Test conditions: Seven-millimetre, 1770 MPa galvanized wires with 300 g/m² zinc inventory; eleven neutral-salt-spray corrosion groups at 45 °C with three wires per group. Corrosion products were removed before optical scanning and tensile testing.

Observation: Across the accelerated series, reported mean steel-section loss reached 18.79%; relative to the uncorroded reference, elongation, yield strength and source-defined actual ultimate strength fell by 43.65%, 11.51% and 13.48%, respectively.

Limitations: These are within-study advanced-damage endpoints, not a coating-only effect or a measured first-steel-exposure time. The product, exposure and cleaning history differ from other salt-spray studies.

S1-32. Cao et al. (2003) [52] — C2 / C3 — section-loss counterexample

DOI: 10.5006/1.3277586

Source locations: Printed pp. 548–553, experimental procedures, Figures 1–8 and fracture observations.

Test conditions: Nominally 1.8 GPa bridge wires in bare, fully acid-stripped and locally stripped galvanized preparations underwent 6 h wet/dry cycles at 49 °C in dilute chloride or sulfate media at pH 2.0–4.6, with selected preloads. Per-condition independent specimen counts were not specified.

Observation: Ultimate load and elongation losses tracked corrosion depth or remaining section under the sampled conditions; fracture surfaces retained ductile dimples and no intergranular cracking was observed.

Limitations: The authors could not reliably determine bulk hydrogen because residual rust affected measurement. The negative cracking observation is limited to this protocol; acid stripping prevents an isolated intact-zinc comparison.

S1-33. Xue and Shen (2020) [111] — C3 — inherited corrosion before dry fatigue

DOI: 10.1016/j.engfailanal.2020.104440

Source locations: PDF pp. 2–5 and 9, specimen/pre-corrosion methods, Table 3 and within-program model check.

Test conditions: Seven-millimetre zinc-coated wires with approximately 50 µm coating underwent 15–90 d acid salt spray in six duration groups, with five wires per duration, followed by mechanical fatigue after exposure.

Observation: The reported fatigue results and mass-loss-derived mean corrosion depth describe pre-damaged wires; the mean-depth estimate exceeded nominal coating thickness in the advanced groups.

Limitations: Mean depth does not locate the first exposed steel site. Its fitted model is checked on the same experimental program, and a later Xue–Shen publication reuses baseline mechanical entries; neither supplies independent coating-to-service-life validation.

S1-34. Cai et al. (2026) [112] — C3 — pre-corrosion and subsequent fatigue

DOI: 10.1016/j.corsci.2026.113934

Source locations: PDF pp. 4–9 and 13, coating and corrosion methods, Table 4 and fatigue results.

Test conditions: Approximately 7 mm, 2100 MPa Zn–10Al–RE-coated wires were exposed to 5 wt.% neutral salt spray for 0, 90, 120 or 150 d before fatigue at 10 Hz. Table 4 identifies three specimens at each listed duration and nominal stress setting.

Observation: Within this product series, longer pre-exposure accompanies shorter reported fatigue life at the tested settings.

Limitations: The article labels a load quantity inconsistently as amplitude or range; its numerical fatigue points are not pooled with other papers. Neither the first local steel exposure nor cable performance was measured.

S1-35. Jiang et al. (2024) [27] — supporting — average-loss measurement

DOI: 10.26599/HTRD.2024.9480040

Source locations: PDF pp. 1, 3–6, accelerated-wire methods and resistance/gravimetry comparisons. The English article is a translation of the 2023 Chinese publication, one study family.

Test conditions: Forty-eight 5 mm galvanized wire segments were prepared for salt-spray exposure; six lost specimen labels, leaving 42 for the reported method comparison. Wires were cleaned before gravimetry and selected surface scans.

Observation: Reported mean discrepancies between resistance- and mass-loss-based estimates were 2.5% for uniform corrosion and 3.8% for pitting in the tested groups.

Limitations: Agreement between two average-loss proxies does not separate zinc and iron partial currents or measure the minimum remaining steel section; no fatigue or cable-capacity endpoint was tested.

S1-36. Recio et al. (2011) [53] — supporting — alkaline hydrogen boundary

DOI: 10.1016/j.corsci.2011.05.023

Source locations: PDF pp. 2–7, wire/tendon preparation, alkaline exposure and mechanical/hydrogen measurements.

Test conditions: Galvanized 5 mm prestressing wire and 7-wire tendon were studied in alkaline pore solutions at pH 12.5–13.5, including 80% UTS sustained load and slow-strain-rate tests. Mercury-displacement outgassing measured released diffusible hydrogen after separate exposures.

Observation: In the pH 13.5 program, local coating consumption was documented; across the specified 6–48 h exposure series, the largest reported maximum-strain reduction was about 30%.

Limitations: The alkaline concrete-contact exposure is not atmospheric bridge-cable corrosion. Outgassed hydrogen is not the entire retained population or a same-specimen measure of hydrogen active at fracture.

S1-37. Chehrazi et al. (2023) [92] — supporting — dry saddle-contact fretting

DOI: 10.1111/ffe.14064

Source locations: PDF pp. 4–7 and 11–15, specimen origins, Table 1, fretting-life entries and fracture/microstructure discussion.

Test conditions: Bare and galvanized stay-wire specimens came from different strand products and had diameters of 5.24 and 5.39 mm; the galvanized layer was approximately 25–30 µm. A small-scale wire/pad rig tested dry fretting at several contact forces and stress ranges.

Observation: The source reports some bare-wire runouts where galvanized specimens fractured within the test limit, alongside wide life scatter within nominal settings.

Limitations: Diameter, original surface and substrate differences prevent assigning the between-product outcome to zinc alone. The test does not measure corrosion, hydrogen or full-cable capacity.

S1-38. Duan et al. (2024) [113] — supporting — existing hanger model

DOI: 10.1016/j.engstruct.2024.118484

Source locations: PDF pp. 1, 3, 6, 9 and 11, three-stage equations, train–bridge loading and validation comparison.

Test conditions: A tied-arch hanger calculation combines zinc-coating corrosion, pit-to-crack transition and crack growth with train–bridge interaction and evolving wire load/section.

Observation: The published method already implements a coating-to-hanger-stage sequence and compares model output with a continuum-damage calculation.

Limitations: That comparison is between calculations, not a measured zinc-loss-to-wire-break history on the modeled hanger. The scenario lifetime cannot validate a general bridge-cable service-life law.

S1-39. Guo et al. (2025) [114] — supporting — existing wire/cable model

DOI: 10.1016/j.engfracmech.2025.111603

Source locations: PDF pp. 1, 9–11 and 18, model inputs, cellular-automaton comparison and concluding scope.

Test conditions: The hot-dip-galvanized wire/cable calculation separates pre-crack corrosion-fatigue and post-initiation crack growth in a tropical marine scenario, with coating and steel kinetics as inputs.

Observation: The first-stage formulation is compared with a cellular-automaton simulation; the crack-growth component is compared with previously published fatigue tests.

Limitations: These checks do not constitute a prospectively observed coating-to-crack-to-cable-failure sequence or a measured field lifetime. Its stage model is prior work, not a new result of the present review.

S1-40. Li et al. (2007) [37] — C1
DOI: 10.61558/2993-074x.1826
Source locations: Scanned article pp. 297–300, methods and Figures 2–4.
Test conditions: Galvanized 5 mm stay-cable wire was exposed to 5 wt.% NaCl in 12 h spray/12 h stop cycles at applied tensile forces of 0, 10, 15 and 22 kN.
Observation: First visible red rust was reported at 16 cycles for 15–22 kN and 23 cycles for 0–10 kN. The source also reports a higher polarization-derived current at 22 than at 0 kN.
Limitations: The source gives no independent-wire count or uncertainty for rust onset. The sampled visible-rust endpoint is later than possible microscopic steel exposure; this one product/protocol does not establish a thickness threshold or service-life rate.
S1-41. Zhang et al. (2024) [28] — C1
DOI: 10.1016/j.jmrt.2024.11.023
Source locations: PDF pp. 3–4 and 10–15, test design, product analysis and mass-loss correction.
Test conditions: Finished galvanized high-strength steel wires underwent constant-current corrosion over several exposed lengths and target losses. The source reports 195 test sets; the independent specimen denominator is not resolved from the reported grouping.
Observation: Measured mass loss differed from a Faraday-based estimate; microscopy and product analysis recorded later Fe-bearing corrosion products.
Limitations: The measured bulk loss and applied charge do not separately identify Zn and Fe partial currents, first microscopic Fe attack or remaining local load-bearing section. Imposed current does not supply a field time conversion.
S1-42. An et al. (2026) [99] — supporting — existing cable model
DOI: 10.1016/j.jcsr.2026.110540
Source locations: PDF pp. 5–6 and 11–13, model inputs, Tables 3–4, Figure 16 and comparison with cable tests.
Test conditions: A stochastic-pit wire model feeds parallel-wire cable fatigue calculations; published single-wire data inform the wire stage, and two published corroded 69-wire cable specimens are used for a cable-level comparison.
Observation: The study compares predicted cycles to the third and seventh wire breaks with those endpoints in each of two published 69-wire cable tests.
Limitations: The cable calculation starts with pits represented as crack-like defects; no observed coating-loss history or independent full coating-to-capacity validation is supplied. The two-cable comparison is narrower than general cable-level validation.
S1-43. Lan et al. (2026) [82] — C3
DOI: 10.1111/ffe.70274
Source locations: PDF pp. 2–4, test design, Figures 1–4 and Appendix A.

Test conditions: Accelerated corrosion-fatigue tests used a machined 1.5 mm exposed-steel window and imposed anodic potential. Ninety new outcomes were combined with 71 observations from an earlier report on the same wire batch.
Observation: Mean life under one loading setting varied nonmonotonically with the imposed potential difference.
Limitations: The 71 reused observations are not new independent tests. The exposed window bypasses intact Zn, and the proposed competition between dissolution and crack growth was not resolved by a continuous crack-history measurement.
S1-44. Liu et al. (2020) [91] — C3 — corrosion-fretting
DOI: 10.1061/(ASCE)BE.1943-5592.0001642
Source locations: PDF pp. 4–8, wire/contact preparation, Figure 10 and Table 2.
Test conditions: Galvanized 5 mm wires with approximately 0.05 mm Zn coating were tested in interwire contact under imposed anodic currents of 0, 0.005, 0.010 and 0.015 A; five fatigue-life values are reported per current group.
Observation: The group means fell from approximately 3.98 × 10^5 cycles at 0 A to 1.60 × 10^5 cycles at 0.015 A, alongside measured contact scars and cracks.
Limitations: The current, masked exposure and contact fixture define an accelerated laboratory history; the groups do not isolate atmospheric coating loss or quantify a change in full-cable load capacity.
S1-45. Wang et al. (2026) [115] — supporting — existing cable model
DOI: 10.1016/j.conbuildmat.2026.146912
Source locations: PDF pp. 3–4, 8–14 and 16, methods, cable case and Appendix Tables A2–A3.
Test conditions: The model combines published wire data with eight new static and six new simultaneous corrosion-fatigue wire tests, then computes an idealized 100-wire parallel cable.
Observation: The study already implements wire-to-cable load redistribution and reliability calculations under prescribed corrosion states.
Limitations: The six dynamic wire tests lack a coating-to-fracture history; no measured cable failure or retained-capacity history tests the 100-wire case.
S1-46. Xue and Shen (2025) [83] — C3
DOI: 10.1061/JBENF2.BEENG-7148
Source locations: PDF pp. 4–6, experimental methods, Table 4 and Figures 6–12.
Test conditions: Galvanized 5 mm hanger wires were dry precycled at four nonzero levels before seawater corrosion-fatigue; The methods report 20 loading cells × four nonzero precycle levels × five wires (400 tests); Table 4 lists 80 group means, not 400 individual outcomes.
Observation: Several higher precycle levels have longer remaining corrosion-fatigue life, precluding a monotonic-penalty summary.
Limitations: No zero-prefatigue comparator is included. Sheath-breach timing is assumed and pit-to-crack timing modeled; neither measured endpoint maps the test to bridge service.
S1-47. Guo et al. (2026) [38] — C1 / C3
DOI: 10.1016/j.cscm.2026.e06084
Source locations: PDF pp. 3–11, specimen allocation, Figure 4 and Figures 10–14.
Test conditions: Two matched loaded/unloaded CASS series each use nine environments × nine exposure durations = 81 long 5 mm galvanized wires; 162 are reported overall, or one wire per environment–duration–load cell. After exposure each wire was cleaned in 10 wt.% HCl for 20 min and brushed, then cut into a 50 cm fatigue and a separate 70 cm pit/tensile segment.
Observation: White Zn products preceded sampled local red rust; cycling was associated with earlier red rust, and the study also reports pit depth, strength and fatigue endpoints within its protocol.
Limitations: No within-cell independent-wire replication is reported. Repeated diameter readings and separate segments are not additional condition replicates or one registered crack history; post-exposure acid cleaning changes the observed surface. Stress-range/amplitude wording is inconsistent and the endpoints are not a service-time conversion.
S1-48. Yan et al. (2024) [42] — C1 — finished-wire pit/section–tensile series
DOI: 10.1016/j.conbuildmat.2024.135845
Source locations: PDF pp. 2–5 and 14–15, Methods, scanning and tensile results.
Test conditions: Thirteen CASS exposures of 48–1416 h used five exposed 7 mm galvanized wires per time point. Before mass and geometry assessment, 12% HCl was applied for 1–2 min; three unused wires estimated cleaning loss.

Observation: Scanned pit and minimum-section geometry and tensile endpoints changed with corrosion state within this accelerated exposure series.
Limitations: Five wires per time point are exposure allocations, not independent chamber runs or necessarily five valid tensile results. The post-cleaning scan-to-tension protocol does not record first local iron attack or register a measured pit to a crack-initiation site.
S1-49. Gao et al. (2023) [44] — C1 — field-origin geometry contrast
DOI: 10.1016/j.istruc.2023.03.054
Source locations: PDF pp. 1, 5–7 and 12–13, Table 3 and mechanical results.
Test conditions: Wires recovered after 18 years of bridge service underwent six additional salt-spray durations plus a control; five wires were allocated per reported corrosion group.
Observation: The study relates later scanned section geometry to breaking force and strain on already serviced wires.
Limitations: Prior service/coating history is confounded with subsequent salt spray. The source's stated approximately 0.85 mm diameter conflicts with its 2.23–2.44 mm² intact-area table; no normalized cross-product comparison is made.
S1-50. Lan et al. (2024) [74]; Bing et al. (2025) [75] — C3 — linked exposed-window fatigue and model reports
DOI: 10.1016/j.jcsr.2024.108666; 10.1016/j.ijfatigue.2025.109040
Source locations: Lan PDF pp. 2–5; Bing PDF pp. 5–6, experimental data and model method.
Test conditions: A polished 1.5 mm exposed steel window on 5 mm wire was tested under imposed potential in 3.5% NaCl. The reports give matching 71 corrosion-fatigue outcomes plus 12 air runouts; Bing cites Lan's test method.
Observation: Measured endpoint lives and runouts support the exposed-window comparison; the later phase-field paper reconstructs pit-to-crack behaviour from the life series.
Limitations: Specimen-level identity is not certified, so the matching 71 + 12 results are conservatively one experimental family. Initial coating loss is bypassed and the simulated initiation history was not observed.
S1-51. Wang et al. (2020) [69] — C3 — machined-notch saline/air contrast
DOI: 10.1016/j.tafmec.2020.102571
Source locations: PDF pp. 2–5, Methods, Tables 1–2 and damage model.
Test conditions: Galvanized 7 mm wire had a 1 mm machined semi-ellipsoidal notch; air and 3.5 wt.% NaCl droplet-fed fatigue each list one specimen at four source-defined stress ranges.
Observation: Each listed simultaneous saline-fatigue life is shorter than the corresponding air life; a numerical damage model reproduces the endpoint ordering.
Limitations: One listed specimen/condition and a manufactured notch do not resolve natural pit initiation. The source's symbol σa is defined as σmax − σmin, a stress range, not a conventional amplitude.
S1-52. Yang et al. (2026) [76] — C3 — independent exposed-spot model endpoint check
DOI: 10.1016/j.ijfatigue.2026.109618
Source locations: PDF pp. 6 and 9–10, test protocol, Tables 4–5 and model validation.
Test conditions: Six 7 mm wires with 5 mm circular areas exposed to solution were tested in 3.5% NaCl under imposed potential independently of the model-calibration dataset. The initial coating state was not reported.
Observation: Six measured corrosion-fatigue lives and post-fracture features were compared with peridynamic predictions; source-listed life errors span −8.7% to 17.5%.
Limitations: Dynamic pit growth and crack-initiation onset were not observed in situ during cycling. The initial coating state and its evolution were not reported. The validation target is wire life, not cable capacity.
S1-53. Wang et al. (2023) [89] — C3 — local saddle-contact zinc loss
DOI: 10.1016/j.triboint.2023.108711
Source locations: PDF pp. 2 and 8, contact method and Table 5 EDS results.
Test conditions: A 1.4 mm galvanized wire contacted a 55 mm saddle over 48° in 3.5% NaCl; separate tests stopped at different cycle counts.
Observation: Worn-surface EDS zinc mass fraction declined from 94.26% initially to 0.26% at 5 × 10⁴ cycles, with strong local iron enrichment.
Limitations: The source's five described experiments are cycle-stop points, not five independent wires per point. Local EDS does not measure total coating inventory or cable capacity; related contact reports may overlap.
S1-54. Chong et al. (2024) [90] — C3 — wire/saddle versus wire/wire contact
DOI: 10.1016/j.wear.2024.205363

Source locations: PDF pp. 2–4, contact geometries and local EDS measurements.
Test conditions: A 1.4 mm galvanized wire with 7–8 µm zinc was tested in aqueous contact against a saddle or another wire with different contact lengths and slips.
Observation: Local worn-surface zinc fell to 0.13% at 4 × 10^4 cycles in one saddle condition and 0.26% at 7 × 10^4 in a wire–wire condition.
Limitations: Contact geometry and slip co-vary; per-condition independent n is not established. The local scar result is not a counterface-only effect, full-wire coating measure or cable-capacity result.
S1-55. Yao et al. (2025) [116] — C1 / C3 — mixed-coating stage and fatigue report
DOI: 10.1016/j.cscm.2025.e05042
Source locations: PDF pp. 2–3 and 6–10, specimen allocation and stage/fatigue figures.
Test conditions: Five-millimetre wires were exposed in 5% NaCl under 0.4 A; the source allocates 60 polymer-overcoated and 12 galvanized-only wires and later describes 48 fatigue specimens.
Observation: The paper reports corrosion-stage products, tensile loss and fatigue outcomes within a mixed-coating programme.
Limitations: The 48 fatigue specimens are not allocated by coating type, so their values cannot be assigned to the 12 galvanized-only wires or used as a Zn-only fatigue ranking.
S1-56. Xue et al. (2021) [117] — C1 / C3 — corroded-wire geometry and tensile response

DOI: 10.1016/j.istruc.2021.04.082

Source locations: PDF pp. 2–4, Tables 1–2; pp. 5–7, Figures 5–8 for model construction and results.

Test conditions: Five-millimetre galvanized wire, source-reported coating mass 355 g/m²; 40 g/L NaCl acidified to pH 3.1 at 50 ± 5 °C; five wires at each of seven sampled exposure durations (35 wires total), followed by 3D pit scanning and tensile tests.

Observation: Specimen-level pit descriptors and static tensile parameters are reported. Randomized pit geometry is used in finite-element failure calculations.

Limitations: The model uses the reported experimental series for its constitutive and pit inputs, without an independent held-out wire test. The source text and Table 1 disagree on some corrosion percentages; no disputed percentage is pooled. There is no registered first steel attack, fatigue history, hydrogen endpoint or cable-capacity test.

S1-57. Yuan et al. (2021) [39] — C1 — Galfan corrosion and local iron products

DOI: 10.1016/j.conbuildmat.2021.125534

Source locations: PDF pp. 2–3, Tables 1–3 and Figure 5; pp. 4–6, Figures 6–9; pp. 9–11, Figures 13–14 and model application.

Test conditions: Nominal 5 mm (measured 5.25 mm), 1860 MPa Galfan wire, source-reported coating mass greater than 337 g/m²; continuous 5 ± 0.5 wt.% NaCl spray at 45 ± 2 °C; 175 allocated specimens across 20 sampled times.

Observation: Local red or brown products and iron-containing products occur in the 510–784 h interval. This precedes the source-defined 1088 h average-depth coating endpoint; the paper also fits time-dependent uniform-loss and pit-factor models.

Limitations: The 1088 h endpoint uses an approximately 30 µm coating thickness inferred by the source from greater than 337 g/m²; that conversion cannot be reproduced by ordinary coating mass–density arithmetic and is not a measured first local steel exposure. Its sheet-based field-time conversion is not observed bridge life. Yuan is also a Zhao et al. (2023) [20] coauthor, and product specifications overlap; this is a distinct reported exposure/model protocol, not independent product or batch replication. Zhao et al. [103] import this coating-loss relation.

S1-58. Xu et al. (2026) [84] — C3 — exposed-notch environmental fatigue contrast

DOI: 10.1061/jmcee7.mteng-21066

Source locations: PDF pp. 2–3, Tables 2–4 and methods; pp. 7–9, Tables 10–11 and Figure 11.

Test conditions: Finished 5.2 mm galvanized wire with a deliberately machined 100 µm-deep steel-exposing cut before cycling; at Δσ = 360 MPa, 35 °C and 10 Hz, the air, humid and 3.5% NaCl groups report 15, 10 and 10 wires, respectively.

Observation: At the source-defined Δσ = σmax − σmin = 360 MPa, Table 11 reports group-mean lives of 203,498 (air), 134,754 (humid) and 85,538 (salt spray) cycles, with COVs 28.57%, 11.66% and 14.08%.

Interpretation limits: Although the source calls S a stress amplitude, its stated extrema define a full stress range; conventional amplitude is 180 MPa at Δσ = 360 MPa. The exposed spot is pre-cut, so the comparison neither measures natural coating breach nor converts accelerated cycling into bridge life. At Δσ = 240 MPa in air, one 2×10^6-cycle runout was omitted from the source mean; the complete source table is therefore not an uncensored life curve.

S1-59. Hu et al. (2023) [19] — C1 — five coating products and prepared-notch products

DOI: 10.1108/acmm-07-2023-2858

Source locations: PDF p. 3, preparation; p. 5, Figure 4 and salt-spray design; p. 6, Section 3.2, polarization/impedance; pp. 6–8, Figures 7–10 and prepared-notch observations.

Test conditions: Six-millimetre, 2100 MPa-grade galvanized starting wire was partly redipped to make Zn–6Al–1Mg or Zn–10Al–3Mg variants at different bath temperatures (430 and 480 °C). Five product coatings, with corrosion-area thickness described as 50–60 µm, were compared under acetate salt spray; replicate counts and dispersion were not reported. The Zn–6Al–1Mg and Zn–10Al–3Mg preparations also underwent polarization and impedance measurements after 0.5, 24 and 144 h immersion in 3.5% NaCl.

Observation: Figure 4 reports first visible red rust at 8, 19, 24, 25 and 57 days for Zn, Zn–5Al, Zn–10Al, Zn–6Al–1Mg and Zn–10Al–3Mg products. At a separately prepared 2 mm-wide, 1 mm-deep steel-exposing notch after 500 h, Zn–Al–Mg showed no visible red rust and Zn/Mg-containing products, while the Zn–Al comparator showed red rust. The electrochemical comparison concerns overall polarization/impedance response, without Zn/Fe dissolution separation or a retained-section endpoint.

Limitations: The ranking concerns specific products, not an isolated coating-composition factor: second-dip temperatures, coating microstructures and possibly local thickness differ, and independent n is unstated. The prepared-notch contrast does not prove restoration of coating integrity, original steel section or mechanical durability; galvanic-current direction and Zn/Fe dissolution were not partitioned.

S1-60. Yuan et al. (2025) [98] — Supporting — wire-strength model versus proposed cable workflow

DOI: 10.1108/acmm-12-2024-3153

Source locations: PDF pp. 5–7, data augmentation, 80/20 split and 2121 prediction cases; pp. 7–9, wire-model comparisons including Figure 10; pp. 9–10, Figure 11 cable workflow.

Test conditions: A 1910-row data set includes interpolated samples; an internal 80/20 split checks regression outputs. A 101 corrosion-time by 21 pre-strain prediction grid gives 2121 modeled cases. Random-pit and equivalent-section finite-element wires are compared with available wire-level strength tests.

Observation: The source compares predicted yield and ultimate strength with corroded-wire test values and proposes a monitoring-to-wire/cable evaluation workflow.

Limitations: Augmented records and 2121 modeled grid points are not independent tested wires or batches. An internal row split does not establish transfer across batches or cable assemblies. The study checks wire strength and proposes a workflow for cable static-performance assessment (Figure 11); the workflow is not validated against measurements of retained axial force or residual ultimate load-bearing capacity in a cable assembly.

S1-61. Doshida and Takai (2014) [57] — C2 — controlled hydrogen-state comparison in machined pearlite

DOI: 10.1016/j.actamat.2014.07.008

Source locations: Printed pp. 94–96, §§2.1–2.4 and Figs. 4–5, material, hydrogen preparation and controls; pp. 97–99, §3.1 and Figs. 6–8, stress–strain and fracture comparisons; pp. 101–102, §3.3 and Figs. 11–13, temperature/rate/content series.

Test conditions: Cold-drawn SWRS82B pearlite, 1838 MPa, machined from 5 mm wire to 3 mm diameter and 20 mm gauge; #1000-polished surface. Basal immersion charge: 8 mass% NH_4SCN at 30 °C for 96 h, giving approximately 1.9/2.6 mass ppm source-defined peak 1/peak 2. A 200 °C, 45 min hold removed peak 1; its uncharged control received the same hold. Initial strain rates were 8.3×10^{-6} to 8.3×10^{-3} s^{-1} in separate parameter series; temperatures were −196, −30, 30 and 70 °C. Both-peak tests at 30/70 °C continued charging during loading, whereas tests at −196/−30 °C followed precharge. TDA used 100 °C/h from room temperature to 500 °C.

Observation: At 8.3×10^{-6} s^{-1}, peak-2-only specimens with approximately 2.6 mass ppm retained hydrogen had reported stress–strain responses overlapping those of the thermally matched uncharged control at each tested temperature. Both-peak specimens showed greater ductility loss at −30, 30 and 70 °C; susceptibility increased at lower rates and higher peak-1 content in the source's parameter series. The matched thermal control makes this a controlled comparison of the source-defined desorption states, rather than inference from desorption temperature alone.

Limitations: Condition-level n, dispersion and exact precharge-to-tensile transfer intervals are not reported. No statistical equivalence or pooled effect is inferred from overlapping curves. The material was uncoated machined pearlite exposed to NH_4SCN; neither its hydrogen-entry flux nor its peak groups are assigned to an atmospherically corroding galvanized wire. TDA and mechanical specimens are distinct; the controlled preparation supplies a treatment-level contrast, not same-specimen detection of hydrogen at the fracture site. Tracer recharge after imposed strain probes changed trapping capacity; vacancy/cluster identity remains the source's interpretation.

Table S1E. Endpoint, specimen/site linkage and validation crosswalk for all 61 S1 source families, ordered by source-note ID (S1-01 to S1-61). A row is a source family or model check, not an independent experiment; entries state reported endpoints and allocation rather than pooled effects. NR means not resolved from the cited methods and results; it does not establish absence of pairing. Methods and inference limits are detailed in the linked S1 source notes.

S1 family	Direct endpoint / check	Reported allocation / check basis	Specimen/site linkage	Unmeasured link
01 Gelfi [21]	Pre-torsion coating cracks; torsion	Batch 9; spray on/off; wire n NR	SEM/torsion site match NR	Fe onset; service failure
02 Zhang [8]	Qualitative desorption/product signals; FIP control-design case	Separate 24 h desorption; 16 FIP total; condition allocation undefined	Desorption/FIP specimens separate; stripped/polished state; grade-based load	Quantitative coating effect; composition ranking; specimen-linked H/fracture history
03 Zhu [14]	Thickness and tensile strength	3 tensile specimens/state	Thickness/tensile pairing NR	Strength scatter; isolated coating effect
04 Zhao [20]	Brown products at sampled 384/1016 h	Two wire products	Product pair; site match NR	First local Fe attack; fracture
05 Hirakami [61]	Charged fracture and TDA H	Drawn/aged states; n NR	TDA/fracture specimen match NR	Fracture-active H; universal limit
06 Enos/Scully [54]	Notched fracture-initiation stress	Notches; permeation/FE estimates	H/local-strain match NR	Coated-wire atmospheric H flux
07 Jia [55]	Elongation by H-supply history	3 tensile tests/condition	TDS plates separate from tensile	Fracture-active H; coated-wire transfer
08 Huang [56]	Tensile, TDS and APT endpoints	Separate specimens	Separate TDS/APT/tensile specimens	Fracture-active H population
09 Nakamura [51]	Wet/dry fatigue by corrosion level	Separate wire tests	Separate wire tests; site NR	Same-wire H and reaction history
10 Elices [60]	FIP time, XRD stress, strength	6/state in prose; FIP tables 8/6	XRD/FIP specimen match NR	Residual-stress-only cause
11 Liu [29]	Pit-linked origin; dry-fatigue life	Two products; allocation NR	Post-test origin; onset timing NR	Coating-only effect; initiation fraction
12 Yang [46]	Section profile; fatigue/runouts	42 wires/7 groups; 1 scan/group	Group level; scan/failure site NR	Same-site pit-to-fracture history
13 Wang [81]	Notched air/saline cycle entries	Condition n unresolved	Notched conditions; specimen/site NR	Oxygen, replicates, coating state
14 Miyachi [80]	Corroded/new-wire life ordering	At $\Delta\sigma$ = 650 MPa: 5 new and 4 corroded fatigue wires; independent corrosion-batch n NR	Different corrosion states; site NR	Population benefit from corrosion

S1 family	Direct endpoint / check	Reported allocation / check basis	Specimen/site linkage	Unmeasured link
15 Li [85]	Fitted potential-transition time	Imposed-current curves; spread NR	Exposed window; no fracture	Complete Zn loss; service life
16 Montalvo [87]	Fretting endurance, scar, crack	Coated/stripped; some reused data	Initiation-site Zn state NR	Cable-contact capacity effect
17 Zhang [93]	FIP time/current; separate TDA	Stripped wire; allocation NR	FIP/TDA histories unpaired	Fracture-active H population
18 Ding [102]	Calculated SCF vs pit position	1×7 idealized strand FE	No measured site history	Observed fracture/cable capacity
19 Lan [101]	Predicted survivor-wire stress	300-wire equal-sharing model	No measured assembly trajectory	Observed redistribution/capacity
20 Li [97]	Predicted saddle-outlet location	Five-wire model; wire calibration	No component validation	Observed component failure/capacity
21 Deng [79]	Dry fatigue after AASS	3 wires/state/duration	Separate polish groups; site NR	Paired pit and section attribution
22 Jarwali [35]	Strand coating damage and red rust	1 strand/cell; 5 wire subsamples	Different strands/time; site NR	Fatigue/rupture/cable capacity
23 Tomatsu [62]	Charged/air notched fracture stress	Air n=1; charged n=3/3/4	Uncoated coupons; local H NR	Finished coated-wire transfer
24 Wang [108]	Contact wear/electrochemistry	150 wear sites; wire n NR	Within-test sites; no fracture	Fatigue fracture/cable capacity
25 Zhou [109]	Rust class and mean tensile strength	3 wires/state; 1 retired cable	Different wires across states	First Fe attack; service life
26 Yang [40]	Mass loss, Fe products, current	3 segments/1 exposed wire	Segments subsamples; Zn/Fe current NR	First exposure; remaining section
27 Zhao [103]	Modal/deflection model checks	Imported loss; 150 pit-coefficient draws/condition	Response not paired to observed damage	Measured coating/crack history
28 Xue/Shen [100]	First nine wire breaks	One 121-wire cable	Stress fit; same-cable break check	Retained force/capacity
29 He [107]	80-kHz bundle response by class/depth	4 recovered wires; 1/class	Same wires at different depths	Coating loss; retained cable force
30 Gu [18]	Coating products through 1200 h	Finished-wire product	Finished-wire series; site NR	First local Fe attack; fracture
31 Wan [43]	Section and tensile loss	11 groups × 3 wires	Exposure groups; specimen/site match NR	Breach time on tested wires
32 Cao [52]	Load, elongation, depth, fracture	Bare/stripped states; n NR	Specimen/site registration NR	Isolated intact-Zn effect; bulk H
33 Xue/Shen [111]	Post-AASS dry fatigue; mean depth	6 durations × 5 wires	Exposure groups; Fe-onset site NR	First Fe attack; service validation
34 Cai [112]	Dry fatigue after 0–150 d NSS	3 wires/duration/stress	Duration/stress groups; site NR	Breach time; unambiguous $\Delta\sigma$
35 Jiang [27]	Resistance/gravimetric loss	48 segments; 42 retained labels	Segment method pair; min-section site NR	Zn/Fe loss; fatigue/capacity
36 Recio [53]	Strain loss; H outgassing	5-mm wire/7-wire tendon; n NR	Separate exposures; active H NR	Same-specimen active H; field transfer
37 Chehrazi [92]	Dry fretting life/fracture	Different bare/coated products; n NR	Between-product groups; site NR	Zn-only effect; corrosion/cable capacity
38 Duan [113]	Three-stage hanger calculation	Model comparison	Model comparison; site match NR	Observed zinc-stage duration
39 Guo [114]	Wire/cable staged calculation	Simulation/literature checks	Model/literature checks; site NR	Independent full-chain validation
40 Li et al. [37]	Visible red rust: 16 vs 23 cycles	Force groups; n/dispersion NR	Force groups; site match NR	Microscopic onset; thickness effect
41 Zhang et al. [28]	Measured loss versus charge estimate	195 test sets; unit unresolved	Test-set pairing NR	Zn/Fe partial current; local section
42 An et al. [99]	Cycles to 3rd/7th wire breaks; 2 cables	Reused wire data; 2 cable tests	Published cable tests; coating link NR	Observed coating stage; capacity transfer

S1 family	Direct endpoint / check	Reported allocation / check basis	Specimen/site linkage	Unmeasured link
43 Lan et al. [82]	Nonmonotonic mean life vs potential	90 new + 71 reused observations	New/reused tests; pairing NR	Intact-coating stage; crack history
44 Liu et al. [91]	Contact scars and fatigue cycles	5 listed fatigue lives/current group	Current groups; site match NR	Field exposure; cable capacity
45 Wang et al. [115]	Computed 100-wire cable reliability	8 static + 6 dynamic wire tests; model	Wire inputs/model; site NR	Measured cable failure/capacity
46 Xue and Shen [83]	Remaining fatigue after prefatigue	5 wires/cell; 400 stated tests; 80 cell means	Sequential loading per wire; site NR	Zero-prefatigue control; sheath timing
47 Guo et al. [38]	Zn products, rust, pits, tensile/fatigue	1 long wire/environment–duration–load cell; separate cleaned sections	Same long wire/cell; split sections; site NR	Same-site trajectory; replicated effect
48 Yan [42]	Scanned pits, minimum section, tensile	13 times × 5 exposed wires; one CASS protocol	Scan then tension; crack site NR	First Fe attack; coating phase
49 Gao [44]	Recovered-wire section and tensile	Six durations + control; 5 wires/group	Recovered-wire groups; site NR	Prior service; diameter conflict
50 Lan [74]/Bing [75]	Local-window fatigue; phase-field fit	71 life entries + 12 air runouts; linked reports	Linked test series; site match NR	Coating loss; observed initiation
51 Wang [69]	Air versus saline notched-wire life	1 listed wire/condition	Notched wire/condition; site NR	Natural pit; coating-stage history
52 Yang [76]	Six validation lives versus model	6 wires independent of calibration	Six held-out wires; terminal lives only	Time-resolved pit/crack onset; coating stage
53 Wang [89]	Local scar Zn/Fe EDS	Cycle-stop series; n/stop unresolved	Different cycle-stop groups; n NR	Whole-wire Zn loss; component life
54 Chong [90]	Wire/saddle and wire/wire local Zn	Different contact protocols; n unresolved	Different contact protocols; site NR	Counterface-only effect; cable force
55 Yao [116]	Stage products and fatigue	60 polymer/12 Zn-only; 48 fatigue unassigned	Coating/fatigue pairing NR	Zn-only fatigue relation
56 Xue [117]	Pit scan; static tensile; random-pit FE	7 durations × 5 wires = 35; FE from same series	Pit/tensile per wire; first-attack site NR	First Fe attack; independent FE validation; fatigue
57 Yuan [39]	Coating loss; local iron products; pit model	175 specimens / 20 sampled times	Destructive time groups; batch relation to [20] NR	Measured first Fe time; matched coating map; service life
58 Xu [84]	Air/humid/NaCl fatigue lives at $\Delta\sigma$=360 MPa	15/10/10 wires; means and COVs reported	Same pre-cut notch protocol; different wires	Natural breach; first crack; cable life
59 Hu [19]	Visible red rust; prepared-notch corrosion products	5 coating products; independent n/dispersion NR	Pre-cut notch and products; serial site map NR	Composition-only effect; force/crack endpoint
60 Yuan [98]	Wire static-strength model check; cable workflow	1910 rows incl. interpolation; 2121 modeled grid	Internal row split; wire data; no tested cable	Independent-batch check; measured cable force
61 Doshida [57]	Desorption-state tensile/RA contrast	Matched thermal control; n/spread NR	Controlled preparation groups; TDA/tensile separate	Galvanized uptake; fracture-site H; field transfer

Visible rust is a sampled detection endpoint, not first microscopic iron attack. Guo S1-47 has one reported long wire per condition cell and cleaned, separate test sections; Yan S1-48 scans cleaned wires before tensile testing without registering a pit-to-crack site or first iron attack. Lan/Bing S1-50 are conservatively one 71-plus-12 experimental family pending specimen-level proof. For model use, Yang S1-52 checks six held-out terminal wire lives, An S1-42 checks third/seventh break cycles in two published 69-wire cables, and Yuan S1-60 checks wire yield and ultimate strengths; the intended endpoint determines which check is relevant. Xue/Shen S1-28 compares the first nine breaks after boundary-stiffness adjustment to wire stress measured in the same 121-wire cable and therefore addresses same-test reproduction. Retained axial force is the axial-force response under specified loading or displacement, restraint and damage conditions. Residual ultimate load-bearing capacity is the maximum force the damaged assembly can sustain under a prescribed loading procedure and failure criterion. The proposed future main-cable study in Section 8 would assess the wire-level axial-force distribution at stated sections of a specified panel after a break, under prescribed end displacements and cable-band restraint. Retained axial force is checked against assembly-force measurements; residual ultimate load-bearing capacity requires corresponding residual-resistance or failure-limit measurements, or a model independently validated against such tests. Force redistribution alone does not validate capacity, and wire-strength or break-cycle agreement alone validates neither cable output. Contact-scar EDS in S1-53/S1-54 does not measure whole-wire zinc loss, S1-45 remains an idealized cable calculation, S1-57/S1-04 share a manufacturer and product specifications without established batch identity, and S1-58 starts with a prepared steel-exposing notch. These allocations neither join the records into an intact-coating-to-capacity history nor establish an absence of stronger studies elsewhere.

Table S1F. Evidence roles of 34 source-assessed DOI records. Ten records belong to nine families used for detailed comparisons; 24 records provide context for their stated endpoints. Each entry identifies the reported endpoint and its interpretation limit. The accompanying search archive supplies DOI, preparation, study unit and source location.

Search ID	Reported endpoint	Evidence role / interpretation limit
S004	Zn/Fe products; tensile	Context: inhibitor/current factors; no per-cell dispersion
S008	Product strength and torsion	Context: manufacture; no corrosion endpoint
S013	Scanned pits, section and tensile	Detailed S1-48; no first Fe attack or phase relation
S041	Recovered-wire pit shape	Context: cleaning removes coating state
S062	Recovered-wire section and tensile	Detailed S1-49; prior service and diameter conflict
S107	Residual stress and FIP	Context: prestressing wire; distinct H history
S113	Charged-wire crack path	Context: cathodic H; no coated exposure
S116	Notched H mechanics	Context: notch and cathodic supply
S121	Controlled desorption-state tensile/RA response	Detailed S1-61; thermal control; machined pearlite
S123	Preloaded notch cracking	Context: cathodic environment; prepared flaw
S124	Lamellar spacing and H	Context: controlled pearlite; no coated wire
S128	Drawing and H traps	Context: ferritic–pearlitic state differs
S139	Applied potential and H	Context: zinc removed before key test
S151	Drawn-wire H cracking	Context: cathodic supply; no coating history
S192	Multiaxial H fracture	Context: external H; no bridge coating
S202	Prefabricated pits and fatigue	Cited context: prescribed defect; no first breach
S203	Later phase-field fit to matching series	Detailed S1-50; no independent test series proved
S214	Corrosion-fatigue model	Cited context: model stages not site-observed
S222	Air/saline notched-wire life	Detailed S1-51; no natural pit or coating history
S230	Six model-check fatigue lives	Detailed S1-52; no in-situ initiation; initial coating unreported
S237	Original 71 local-window fatigue tests	Detailed S1-50; no intact-coating stage
S238	Multiple-pit notch fatigue	Context: notch surrogate; no active coating
S256	Pre-notched crack growth	Context: propagation after imposed defect
S257	Pit/crack geometry fatigue	Context: prefabricated defects
S275	Stress-ratio dependence	Context: mean stress, not coating onset
S291	Local saddle-scar Zn/Fe EDS	Detailed S1-53; n per cycle-stop unresolved
S294	Small-wire saddle contact	Context: related campaign; overlap unresolved
S295	Galvanized fretting model	Context: reuses Chehrazi fretting data; no new wet-corrosion test
S296	Stress-corrosion response	Context: accelerated electrochemistry
S313	Local wire/saddle and wire/wire Zn	Detailed S1-54; contact geometry and slip co-vary
S317	Beach-mark crack histories	Context: initiated cracks; no first steel exposure
S322	Ambient-air fretting life	Context: no wet coating corrosion
S344	Wire pit scans and fatigue; reliability model	Context: two wires/condition; coating state unestablished; no cable force test
S351	Corrosion-stage images and fatigue	Detailed S1-55; Zn-only fatigue allocation unresolved

The 24 contextual records remain eligible to inform their stated endpoints; they were not treated as full-text exclusions. S203/S237 are conservatively one experimental family because the reports give matching 71 corrosion-fatigue tests and 12 air runouts, although specimen-level identity has not been proved. Related contact reports may also overlap and are not counted as independent replication.

Table S1G. Candidate transition, reported endpoint and measurement-gap crosswalk. A denotes finished-wire evidence; B, machined pearlite or separately prepared specimens; C, related-material, contact, model or assembly support. The rows join neither specimens nor loading histories and do not constitute a validated serial failure pathway. Source-level details and allocation limits are given in Tables S1 and S1E.

Candidate link	Reported endpoint / source	State variable for matched test	Missing paired measurement	Evidence class
Coating → Fe attack	Sampled red rust [19, 37]; brown products [20]; local Fe products [39]; prepared-notch products [19]	Local Zn continuity; Fe-specific signal; wet time	Registered local coating state and first microscopic Fe attack at the same site	A: finished wire

Candidate link	Reported endpoint / source	State variable for matched test	Missing paired measurement	Evidence class
Fe attack → pit/section	Later pit/section–tensile [42, 43, 117]; split sections [38]	Registered pit profile; A_min; tensile force	Registered first Fe attack and subsequent same-site pit growth	A: finished wire
Hydrogen → response	Corroded-wire H [51]; separate TDS/APT [55, 56]; matched desorption control [57]	Load-time H supply; diffusible H; mechanical response	Controlled perturbation on an initially coated wire; fracture-site H	A/B: design-specific
Defect → fatigue	Dry pre-corrosion [79]; saline notch [81]; pre-cut spot environment [84]; held-out lives [76]	Pit geometry; wetness; first-crack cycle	Time-resolved pit/crack co-location	A/B/model: varied
Wire → cable	Two 69-wire cables: 3rd/7th breaks [99]; one 121-wire cable: 1st–9th breaks [100]; wire-model/cable workflow [98]; calculated model [115]	Break order; force recovery; retained axial force	Initial coating paired with retained axial force or residual ultimate load-bearing capacity	C: assembly/model

Table S1M. Method and specimen-history comparison for nine key wire or pearlitic-substrate study families used in Section 4. TDA/TDS denote the source's thermal-desorption analysis/spectroscopy; APT is atom-probe tomography; FIP is the accelerated ammonium-thiocyanate fracture test; NR means not established in the cited source. Each method arm is a record, not an independent experimental confirmation. A hydrogen measurement and a mechanical test on separate specimens are not paired evidence for the hydrogen population active at fracture.

Study / arm	Specimen / surface	Supply; T; j or potential	Transfer / extraction	Mechanical endpoint	H–mechanical pairing
Hirakami [61] / TDA + SSRT	Drawn 5 mm SWRS82B; notch; as-drawn/aged	0.1 N NaOH + 5 g/L NH_4SCN; 30 °C; 0.2–0.5 mA/cm² precharge; continued in SSRT	Separate TDA; 100 °C/h to 600 °C	Fracture-stress ratio; constant-load time	Different specimens
Zhang [8] / TDA + FIP	6 mm coated or HCl-stripped/polished products	20 wt.% NH_4SCN; 50 °C; no imposed j	TDA after 24 h; 100 °C/h to 850 °C	Separate FIP; 70/80% grade Fmax	Different specimens; allocation NR
Elices [60] / FIP	5.20 mm drawn/stabilized prestressing wire	20 wt.% NH_4SCN; 50 ± 1 °C; no imposed j	Hydrogen amount NR	Rupture time at 80% state Fmax	Mechanical test only
Nakamura [51] / thermal release	1570 MPa finished galvanized wire; corrosion level 2	Wet gauze; stressed programme 20 °C; unstressed programme 40 °C	Dry-ice storage; 100 °C/h extraction	Fatigue on other wires; 64 Hz	H/fatigue on different wires
Recio [53] / outgassing	5 mm galvanized prestressing wire/strand	pH 12.5–13.5 alkaline solution; 80% UTS; ≤48 h	Room-T Hg displacement; released H	Separate tensile/SSRT	No same-specimen H/fracture link
Enos [54] / permeation + TDS	Drawn ASTM A416 strand wire; notched CERT	Saturated $Ca(OH)_2$; controlled cathodic potential; 12 h precharge, maintained	Separate permeation/TDS; 2.5–10.3 °C/min TDS	Notched CERT/fracture initiation	Measurements on different specimens
Jia [55] / tensile	3 mm machined section from 7 mm wire	0.2 M NaOH + thiourea; 298 K; 1 mA/cm² ex-situ plus in-situ arms	Precharge/transfer or continued charge by arm	0.01 mm/min tensile; n=3/condition	TDS plates separate
Jia [55] / TDS	Separate 30 × 6 × 2 mm plates	0.1 M NaOH + thiourea; 5 mA/cm²; duration NR	100/200/300 K/h heating	No test on TDS plate	Not tensile specimen
Huang [56] / tensile	2.5 mm round; 50/10 mm gauges	0.1 M NaOH + NH_4SCN; 50 mA/cm²; 24 h	0/12 h room-T hold by arm	Tensile; n=3/condition	TDS/APT separate
Huang [56] / TDS	Separate 10 × 5 × 1 mm cuboids	Same H charge as tensile arm	0/2/12 h room-T holds; source Fig. 3D/E (highly strained specimens): 100/150/200 °C/h to 500 °C (Kissinger)	No test on TDS cuboid	Not tensile specimen

Study / arm	Specimen / surface	Supply; T; j or potential	Transfer / extraction	Mechanical endpoint	H–mechanical pairing
Huang [56] / APT	Independent D-tip matchsticks	0.1 M NaOH/D_2O; 2.2 V; 45 s	Cryotransfer or room-T hold	Local D distribution, no tensile	Different charge and specimen
Doshida [57] / tensile	Machined 3 mm × 20 mm; 1838 MPa	8 mass% NH_4SCN; 30 °C; 96 h	Peak 2: 200 °C/45 min; matched uncharged heat control	Stress–strain/RA; 4 temperatures; n NR	Controlled groups; TDA separate
Doshida [57] / TDA	Matched pearlite; #1000 polished	Immersion charge; no imposed j/potential	100 °C/h; room T–500 °C	Source-defined peak 1/2 release	Does not locate H at fracture

For Doshida's both-peak tensile branch, 30/70 °C tests continued solution charging; −196/−30 °C tests followed precharge. Peak-2-only specimens received the selective-desorption hold and were compared with a thermally matched uncharged state.

Table S1N. Selected fretting-fatigue records from Montalvo et al. [87], source Table 2. All six use 30° crossing, P = 1400 N, σm = 320 MPa and σa = 160 MPa in ambient air without grease. Bright denotes the inhibited-HCl-stripped product; rows are source-listed tests, not replicate means.

Wire state	δ* (μm)	Q* (N)	Slip	Scar area (mm^2)	Source cycles
Bright	9	88	PS	0.77	>10^6
Coated	10	73	PS	2.08	>10^6
Bright	81	673	PS	1.16	121,000
Coated	81	754	PS	4.04	174,000
Bright	210	1264	GS	21.02	>10^6
Coated	210	1208	GS	21.78	>10^6

PS, partial slip; GS, gross slip. δ* = δ*FA − δ*FA,0 is the source's effective actuator amplitude, not measured local slip. Q* is tangential force amplitude. Scar area is πaX aY from optical ellipse dimensions. The > signs retain runouts. Different δ* and/or Q* prevent isolated coating-effect ratios; area does not determine local contact pressure.

Tables S2A–S2C retain source-defined records without pooling different experiments. Wang's entries have unresolved independent n and aggregation; Li's potential-transition time, cycle and charge columns describe one operational endpoint; Miyachi's entries identify individual fatigue wires at one stress range. NR is not zero or one replicate.

Table S2A. Published failure-cycle entries for air and two neutral NaCl media in Wang et al. [81], source Table 4, p. 12. Group identities and stress extrema follow source Section 2.3 and Table 3, pp. 10–11. These condition-labelled entries are retained as a numerical source record and are not plotted as individual tests, means or effects.

Source labels: air / 0.5% / 3.5%	Δσ (MPa)	σmax (MPa)	σmin (MPa)	Nair (cycles)	N0.5 (cycles)	N3.5 (cycles)
A-1 / D-1 / B-1	360	643	283	41244	34583	34835
A-2 / D-2 / B-2	480	857	377	12864	10702	10881
A-3 / D-3 / B-3	600	1071	471	7229	4388	5769
A-4 / D-4 / B-4	720	1286	566	3531	2709	3351

Source groups A, D and B are air, 0.5 wt.% NaCl (pH 7) and 3.5 wt.% NaCl (pH 7), respectively. Prescribed notches in 7 mm wire were cycled at 0.5 Hz, room temperature and source-reported R = 0.44, with local gauze replenished every 10 min. The source uses σa for the full stress range; the tabulated extrema verify Δσ = σmax − σmin, while amplitude is σa = Δσ/2 here. Source group labels do not establish individual specimens or independent replicates; condition-level n, aggregation and dispersion are NR. The integer cycle entries show a published-table ordering, not a concentration law, uncertainty estimate or treatment effect.

Table S2B. Six source-listed D/E potential-transition conditions in Li et al. [85], with cycles and imposed charge calculated from each reported time. The conversions are numerical descriptions of the operational endpoint, not independent observations.

The operational endpoint is the intersection of fitted potential–time segments D and E. N = f × t and q = j × t, with j = 0.020 A/cm^2, are arithmetic expressions of the same reported times, not independent measurements or replicates.

Condition-level endpoint n and dispersion are not established. Neither complete zinc removal, current efficiency, constituent-specific zinc mass loss nor atmospheric service life is inferred.

Δσ (MPa)	f (Hz)	Endpoint t (s)	N = ft (cycles)	q = jt (C/cm²)
Unloaded	0	10241	0	204.82
180	10	9577	95770	191.54
360	10	6612	66120	132.24
540	10	3400	34000	68.0
360	5	5898	29490	117.96
360	1	4193	4193	83.86

Table S2C. Individual fatigue-wire records at Δσ = 650 MPa from Miyachi et al. [80], source Table 8, p. 11; numerical source for Figure 8A.

Source specimen ID	Wire state / level	Δσ (MPa)	σmin (MPa)	Nf (cycles)
2(new)	new / 0	650	500	112000
3(new)	new / 0	650	500	94000
4(new)	new / 0	650	500	90000
5(new)	new / 0	650	500	75282
6(new)	new / 0	650	500	75000
No.4	corroded / 1	650	500	153000
No.3	corroded / 1	650	500	142000
No.6	corroded / 2	650	500	608000
No.5	corroded / 2	650	500	521000

The five new and four 8300 h salt-spray corroded wires are distinct fatigue specimens tested at minimum stress 500 MPa and 10 Hz. Independent exposure batches and matched starting pit geometry were not reported. Other source stress settings are not pooled into this fixed-stress comparison; numerical non-overlap does not identify a beneficial corrosion effect.

Supplementary Note S1H. Targeted checks against alternative interpretations.

Coating comparisons. Jie (Section 4.1, Figure 19) and Zhao (reference 41) discuss earlier Zn/Zn–Al corrosion-fatigue comparisons [1, 3]. The present comparison combines separate local-corrosion and contact-fatigue observations: Yuan's local Fe-containing products precede its 1088 h average-depth endpoint [39], while Montalvo's interrupted test found a crack with zinc elsewhere in the contact scar [87]. These separate observations support assessing local steel condition before an average coating endpoint or despite zinc elsewhere; they do not establish an intact coating over a crack origin. Guo [38] supplies a corresponding loaded/unloaded exposure programme and later mechanical tests on separate sections of each sampled long wire. Its positive within-programme link is retained with the one-wire-per-condition allocation. Product-specific rust and acid-stripping contrasts do not establish a common intrinsic-alloy ranking. The relation to earlier reviews is specific: Jie [1], Section 2.1 and Figure 4 (PDF pp. 7–8) describes pit-to-short-crack-to-long-crack evolution, while Sections 2.2.1 and 4.6 also cover coating comparisons and engineering assessment. Zhao [3], Sections 1 and 4.1–4.3 (PDF pp. 2–3 and 12–14) already links micro-damage, component response and system assessment. For the assessments in Section 2.1, visible-product time compares the tested preparations within each exposure protocol. Fatigue after local steel exposure is judged using defect geometry and the environment maintained during loading, even when zinc remains elsewhere.

Hydrogen comparisons. Hu [2] combines Huang's APT and other analyses to explain the lower susceptibility of highly drawn pearlite through strong trapping by tangled dislocations. The comparison separates Huang's 12 h desorption–SSRT recovery from retention in independently prepared, 2 h-desorbed APT tips [56]. These observations support hydrogen participation and trapping; the separate preparations do not, on their own, isolate trapping's protective contribution to the between-state elongation-loss difference. Doshida–Takai's thermally matched selective-desorption control [57] tests the mechanical consequence of its retained peak 2 population, detecting no additional ductility loss under that protocol. Hu also discusses Doshida–Takai's non-diffusible-hydrogen negative result. Stabilization [60] and aging [61] improve their respective hydrogen-related responses despite opposite strength changes. These protocol-specific results define neither a pooled susceptibility scale nor a universally inactive high-temperature population. Hu's discussion of drawn-wire trapping and mechanical response (PDF pp. 7–10) and its service-coupling/multiscale outlook (PDF pp. 11–13) already cover these topics. For assessment, Huang's recovery supports hydrogen participation and its APT observations support local retention; Doshida–Takai's matched control addresses the mechanical response of its retained population. These distinctions determine whether a protective attribution has mechanical support.

Validation comparisons. Zhao's review [3], Section 4.3 (PDF p. 14), discusses load redistribution, cable-fatigue models and experimental comparisons, including residual-bearing-capacity assessment. The endpoint comparison distinguishes

calibration, same-source agreement and independent checks. Yang's six terminal wire-life tests [76] are independent of calibration, whereas Zheng and Wang's fitting/checks [77] use the same published sources (main text, Section 5.1). Within cable-break timing, An's two published 69-wire tests [99] check third/seventh breaks; Xue and Shen compare the first nine breaks [100] after adjusting boundary stiffness to stress measured in that same 121-wire cable. Yuan [98] checks wire yield/ultimate strengths; its 1,910 rows include interpolation and its 2,121 cases are numerical grid points. The proposed main-cable-panel assessment is placed in Future Research Directions (Section 8): the target is retained axial-force distribution after a wire break under prescribed end displacements and cable-band restraint. Local resistance measurements describe defect geometry, remaining section and exposure/loading history; break location, broken-end gap and tension recovery are force-transfer inputs. Testing that target requires independent assembly-force measurements; fitting the same force record is calibration or reproduction. Published checks support their measured wire properties or break events; retained axial force requires assembly force measurements, and residual ultimate load-bearing capacity requires ultimate-load tests. The proposal does not calculate force distribution, a safety margin or remaining life.

Table S3. Focused appraisal of the main comparisons

Table S3 summarizes the finding and main limitation of 59 study or protocol appraisals in short phrases. Each row links to a full record in Supplementary Appraisal Notes S3 below. The full findings, limitations and source notes are retained there. These entries supplement the 61-family index in Table S1E and do not enumerate additional independent studies. Model outputs remain identified as such.

S3A. Coating and local steel damage

Study / preparation	Reported finding	Main interpretation limit	Full record / source notes
Zhu [14]	Morphology, thickness and mean strength differ between preparations.	Co-varying processing; undefined dispersion; no corrosion-onset endpoint.	S1-03; S3-A01
Gu [18]	Coating products and electrochemical response tracked during salt spray.	No registered first steel exposure or mechanical-life endpoint.	S1-30; S3-A02
Hu [19]	Visible red-rust times differ among tested preparations.	Unmatched products and histories; alloy chemistry not isolated.	S1-59; S3-A03
Gelfi [21]	Cooling preparations differ in coating-crack depth.	Pre-drawing manufacturing contrast; no atmospheric-onset or universal threshold.	S1-01; S3-A04
Liu product comparison [29]	Product configurations show pit-associated fracture and post-exposure fatigue.	Product confounding; unresolved stress definitions; control is runout.	S1-11; S3-A05
Jarwali [35]	More damage under wet gauze than tested humid-air exposures.	No universal humidity set point; one strand per condition.	S1-22; S3-A06
Zhao [20]	Sampled brown-product times differ between Zn and Galfan products.	Unmatched products; first microscopic steel attack not registered.	S1-04; S3-A07
Li loaded rust [37]	Higher-load groups show earlier visible rust during salt spray.	Independent replication and maintained loading history not established.	S1-40; S3-A08
Guo loaded exposure [38]	Cyclically loaded preparations develop red rust earlier than unloaded.	One wire per condition; separate mechanical and geometry sections.	S1-47; S3-A09
Yuan [39]	Local Fe-bearing products precede the average-depth coating endpoint.	Unreconciled inventory; Zn products do not establish metallic zinc.	S1-57; S3-A10
Yang acid rain [40]	Mass loss varies with stress; highest-stress iron-bearing products.	First steel exposure unrecorded; current does not partition Zn/Fe.	S1-26; S3-A11
Yan [42]	3D pits and minimum section linked to tensile response.	Acid-cleaned specimens; first local steel exposure not recorded.	S1-48; S3-A12
Wan [43]	Section loss accompanies lower strength and elongation.	No first-breach timing or coating-thickness dose–response.	S1-31; S3-A13
Gao [44]	Recovered-wire mechanical states after further salt spray.	Service and new corrosion inseparable; diameter/area inconsistency.	S1-49; S3-A14
Recovered-wire fatigue [45]	Shorter fatigue lives for pitted than uniformly corroded specimens.	Matched source loads; no coating-onset or pooled effect.	Note S3P; S3-A15
Xue [117]	Pit and tensile observations distinguish exposed specimen states.	Early strength ranges overlap; force decrease is modeled.	S1-56; S3-A16
Yang profiles [46]	Profiles locate section minima; low-damage specimens reach stopping limit.	One profile wire/group; runouts; atmosphere and stress-area convention unresolved.	S1-12; Table S1E; S3-A17

S3B. Hydrogen and mechanical response

Study / preparation	Reported finding	Main interpretation limit	Full record / source notes

Study / preparation	Reported finding	Main interpretation limit	Full record / source notes
Nakamura–Suzumura [51]	Low-temperature hydrogen release below 0.2 ppm in tested wires	Study-specific 0.7 ppm criterion; separate hydrogen/fatigue specimens	S1-09; Table S1M; S3-B01
Cao [52]	Section-related mechanical loss under acidic wet–dry exposure	No observed hydrogen cracking; no universal exclusion threshold	S1-32; Table S1M; S3-B02
Recio [53]	Alkaline exposure: hydrogen release and ductility loss	Prestressing specimens; outgassing measures release, not fracture-local hydrogen	S1-36; Table S1M; S3-B03
Zhang [8]	Coated/stripped states show different desorption signals	Preparation/loading controls only; no quantitative coating effect or ranking	S1-02; Table S1M; S3-B04
Enos–Scully [54]	Fracture-initiation stress decreases with permeation-derived hydrogen	Notched configuration; FE interpretation, not atmospheric coated-wire threshold	S1-06; Table S1M; S3-B05
Jia [55]	Mechanical response varies with charging history	Machined specimens; separate TDS; ambiguous elongation assignment	S1-07; Table S1M; Note S3H; S3-B06
Huang [56]	Ductility recovery; retention observed in separately prepared specimens	Separate tensile/TDS/APT preparations; retention does not isolate trap protection	S1-08; Table S1M; Note S3H; S3-B07
Doshida–Takai [57]	Retained peak 2: no additional detected ductility loss	Thermally matched control; population-specific result under tested protocol	S1-61; Table S1M; S3-B08
Elices [60]	Stabilization: lower strength, surface-ferrite residual stress; longer fracture time	Strength, residual stress and force co-vary; replication unresolved	S1-10; Table S1M; S3-B09
Hirakami [61]	Aging increases strength and charged/uncharged fracture-stress ratios	Protocol-specific; no universal threshold or fracture-active peak assignment	S1-05; Table S1M; S3-B10
Tomatsu [62]	Orientation-sensitive fracture stress and paths in miniature specimens	Miniature notch/plasma protocol; nominal plane and local path distinct	S1-23; Table S1M; S3-B11

S3C. Fatigue, contact and sustained loading

Study / preparation	Reported finding	Main interpretation limit	Full record / source notes
Wang: notched environmental fatigue [81]	Solution-exposed entries below air; reversed NaCl concentration ordering	Aggregation unresolved; oxygen unmeasured; no quantitative concentration effect	S1-13; Table S2A; S3-C01
Wang: 1 mm notch/droplet tests [69]	Lower prepared-notch lives during saline-droplet exposure	Single specimens; prepared notch bypasses intact-coating history	S1-51; S3-C02
Lan/Bing: exposed-steel-window family [74, 75]	Exposed-window failures and air runouts in one family	Transition not tracked during loading; intact zinc bypassed	S1-50; S3-C03
Yao: polymer-overcoated/galvanized preparations [116]	Staged corrosion and fatigue across two coating categories	Fatigue allocation by coating category unreported	S1-55; S3-C04
Ma: crack-history updating [78]	Beach-mark histories constrain fitted crack growth	Bayesian updating illustrated using a simulated history	Note S3P; S3-C05
Deng and Deng: prepared-state comparison [79]	Different fatigue responses in polished and unpolished groups	Separate preparations alter geometry and potentially steel section	S1-21; S3-C06
Miyachi: new and salt-spray-corroded wires [80]	Corroded-wire lives exceed new-wire lives at stated stress range	Matched pits and independent batches unreported; benefit not established	S1-14; Table S2C; S3-C07
Xue and Shen: pre-corrosion then fatigue [111]	Fatigue changes after acid salt-spray pre-corrosion	Downstream damaged states; first coating breach not registered	S1-33; S3-C08
Cai: Zn–10Al–RE product [112]	Within-product fatigue changes after neutral salt-spray pre-exposure	Stress terminology prevents numerical pooling	S1-34; S3-C09

Study / preparation	Reported finding	Main interpretation limit	Full record / source notes
Xue and Shen: prefatigue then seawater fatigue [83]	Non-monotonic seawater-fatigue means after prior dry cycling	No zero-prefatigue control; trend not universal	S1-46; S3-C10
Lan: imposed-potential corrosion fatigue [82]	Non-monotonic life across imposed potentials	Unresolved dissolution, hydrogen and geometry; no universal relation	S1-43; S3-C11
Xu: prescribed 100 µm-deep cut [84]	Wet exposure: lower mean fatigue lives after steel-exposing cut	At 240 MPa: air runout omitted from published mean	S1-58; S3-C12
Li: electrochemical endpoint under cycling [85]	Coating cracks and earlier loaded-specimen potential transition	Imposed-current endpoint; neither complete zinc removal nor fatigue failure	S1-15; Table S2B; S3-C13
Liu: salt-spray/pickled starting state [86]	Multiple pit-associated origins in pre-corroded, pickled steel	Already exposed steel; no intact-coating starting state	Note S3P; S3-C14
Montalvo: crossed-wire fretting fatigue [87]	Contact crack with zinc elsewhere in the scar	No verified intact coating over crack origin	S1-16; Table S1N; S3-C15
Wang: reciprocating corrosion–wear [88]	Wear volume rises; normalized coefficient falls with displacement	Normalized wear coefficient is not friction coefficient	Note S3P; S3-C16
Wang/Chong: contact-scar chemistry [89, 90]	Local zinc depletion and iron enrichment in contact scars	Local EDS; differing contacts; reports potentially overlap	S1-53; S1-54; S3-C17
Liu: imposed-current corrosion–fretting fatigue [91]	Mean life decreases with imposed current in contact tests	Accelerated contact protocol; no atmospheric or cable-capacity law	S1-44; S3-C18
Chehrazi: dry saddle contact [92]	Wide galvanized-wire fatigue scatter in dry saddle contact	Different products and contacts; coating effect not isolated	S1-37; S3-C19
Zhang: polarized sustained loading [93]	Non-monotonic fracture times; anodic exposure reduces remaining section	Acid-stripped; separate desorption/fracture treatments; no specimen-linked hydrogen attribution	S1-17; S3-C20

S3D. Model and assembly endpoints

Study / preparation	Reported finding	Main interpretation limit	Full record / source notes
Yang et al. [76]	Six wire-life tests outside calibration check terminal predictions	Retained axial force and residual ultimate load-bearing capacity untested	S1-52; S3-D01
An et al. [99]	Two 69-wire tests check selected break cycles	Retained axial force and residual ultimate load-bearing capacity untested	S1-42; S3-D02
Zheng and Wang [77]	Wire-life reproduction and checks reuse fitted source series	Calibration/within-source agreement; no held-out prediction check	Note S3P; S3-D03
Duan et al.; Guo et al. [113, 114]	Staged models agree with selected calculations	Model comparisons do not independently validate experimental stage chronology	S1-38; S1-39; S3-D04
Lan et al. [101]	Equal-sharing model updates survivor stress after assumed breaks	Common properties; no measured spatial resistance or force recovery	S1-19; Note S3L; S3-D05
Li et al. [97]	Local saddle model predicts configuration-dependent contact fatigue	Specified geometry/loading; no pressure measurement or universal saddle-fatigue limit	S1-20; S3-D06
Ding et al. [102]	Pit-position model predicts greater stress concentration near restrained anchorage	Fixed pit dimensions/end rotation; transfer requires component evidence	S1-18; S3-D07
Zhao et al. [103]	Bridge frequency/deflection check modeled structural response	Structural-response check; coating chronology and lifetime remain unvalidated	S1-27; S3-D08
Yuan et al. [98]	Wire tests check yield and ultimate strengths	Augmented learning data; internal splitting is not batch validation	S1-60; S3-D09

Study / preparation	Reported finding	Main interpretation limit	Full record / source notes
Xue and Shen [100]	One cable records first-through-ninth wire breaks	Same-test stiffness adjustment; no residual ultimate load-bearing capacity test	S1-28; S3-D10
He [107]	Retired-wire bundles show depth-dependent electromagnetic corrosion detection	Laboratory depth limits; detection does not measure mechanical force	S1-29; S3-D11

Supplementary Appraisal Notes S3. Full study-by-study findings and limitations

Record identifiers match the last column of Table S3. Each record retains the reported finding, interpretation limit and detailed source notes for that row.

S3A. Coating and local steel damage

S3-A01. Zhu [14]. Reported finding: Coating morphology, thickness and reported mean strength differ between two dipping preparations. **Interpretation limit:** Temperature, coating development and substrate history co-vary. Three tensile specimens per state; dispersion and error-bar statistic undefined. No quantified phase fractions, specimen linkage or difference test; no corrosion-onset endpoint. **Source notes:** S1-03.

S3-A02. Gu [18]. Reported finding: Coating products and electrochemical responses tracked at sampled salt-spray times. **Interpretation limit:** 5.95 mm/2060 MPa-grade Zn–Al–Mg wire; ~60 µm coating; 5 wt.% neutral salt spray including 960 h. No registered first local steel exposure or mechanical-life endpoint. **Source notes:** S1-30.

S3-A03. Hu [19]. Reported finding: Visible red-rust times differ across five tested coating preparations. **Interpretation limit:** Acetate salt spray; 50–60 µm selected corrosion-area thickness. Separately selected comparators lack common heat and thermal history. Independent counts, dispersion and rust-site thickness are unreported; product times do not isolate alloy chemistry. **Source notes:** S1-59.

S3-A04. Gelfi [21]. Reported finding: Same-batch cooling preparations show through-thickness versus delta-layer-limited coating cracks. **Interpretation limit:** Galvanize-before-drawing C82D route; micrographs precede drawing and fine cracks precede torsion. This manufacturing contrast has no atmospheric-onset endpoint or universal thickness/phase threshold. **Source notes:** S1-01.

S3-A05. Liu product comparison [29]. Reported finding: Different product configurations show pit-associated fracture origins and post-exposure fatigue responses. **Interpretation limit:** Galvanized 5.0 mm/~50 µm and Zn–Al 5.4 mm/~70 µm products confound chemistry, inventory and geometry. Conflicting stress-range/amplitude and force-area definitions preclude stress-resolved comparison. The 2×10^6-cycle control is a runout. **Source notes:** S1-11.

S3-A06. Jarwali [35]. Reported finding: Persistent wet gauze produces more damage than the tested bulk-air humidity exposures. **Interpretation limit:** 19-wire Zn/Zn–10Al strands; 40 °C; applied 0.01% NaCl. One strand per coating/environment/time cell. At 150 d, 60% RH surfaces largely intact; 100% RH attack coating-only; wet gauze gives Zn-strand red rust. No universal humidity set point. **Source notes:** S1-22.

S3-A07. Zhao [20]. Reported finding: Zn and Galfan products first show sampled brown products at 384 and 1016 h. **Interpretation limit:** Coating masses 336/337 g/m^2; products differ in diameter and strength. Source thickness does not reconstruct from mass. Sampled different wires do not register first microscopic steel attack, matched rust-site thickness or a composition-only effect. **Source notes:** S1-04.

S3-A08. Li loaded rust [37]. Reported finding: Visible rust after 16 salt-spray cycles in 15–22 kN groups versus 23 cycles in 0/10 kN groups. **Interpretation limit:** 5 mm galvanized wire; independent replication and maintained force history during exposure not established. **Source notes:** S1-40.

S3-A09. Guo loaded exposure [38]. Reported finding: Cyclically loaded preparations develop visible red rust earlier than paired unloaded preparations. **Interpretation limit:** One long wire per environment–duration–load cell, not replicated wires per cell. Products removed with HCl/brushing; fatigue and geometry/tensile sections are separate. No registered coating-to-crack-site history. **Source notes:** S1-47.

S3-A10. Yuan [39]. Reported finding: Local Fe-bearing products precede the source's average-depth coating endpoint. **Interpretation limit:** Continuous salt spray on 5.25 mm Galfan wire; 175 cut specimens at 20 times. Thickness is not reconciled from mass; Zn products do not establish metallic Zn. Shared manufacturer/specifications with [20] do not establish shared batch or independent product replication. **Source notes:** S1-57.

S3-A11. Yang acid rain [40]. Reported finding: Endpoint mass loss varies with static-stress state; iron-bearing products occur in the highest-stress preparations. **Interpretation limit:** Polished 5 mm test regions; 61 cycles of 12 h spray/12 h without spray. Independent-wire uncertainty, first steel-exposure time and remaining section are unreported; whole current does not partition Zn/Fe. **Source notes:** S1-26.

S3-A12. Yan [42]. Reported finding: Scanned 3D pits and minimum section related to tensile response. **Interpretation limit:** 13 groups × five 7 mm wires, 48–1416 h copper-accelerated salt spray; 12% HCl cleaning for 1–2 min; first local steel exposure not recorded. **Source notes:** S1-48.

S3-A13. Wan [43]. Reported finding: Section loss accompanies lower strength and elongation across the salt-spray series. **Interpretation limit:** Eleven states, three 7 mm wires per group. Maximum reported mean section loss 18.79%; strength/elongation declines 13.48%/43.65%. These downstream contrasts do not locate first coating breach or define a thickness dose–response. **Source notes:** S1-31.

S3-A14. Gao [44]. Reported finding: Recovered-wire mechanical states after further salt spray. **Interpretation limit:** Five wires per group; 18-year service history followed by accelerated exposure. Service and new-corrosion contributions cannot be isolated; stated diameter conflicts with section-area table. **Source notes:** S1-49.

S3-A15. Recovered-wire fatigue [45]. Reported finding: Pitted specimens had shorter fatigue lives than uniformly corroded specimens under matched source loads. **Interpretation limit:** Recovered-wire state and geometry comparison, not a measured coating-onset or cross-study pooled effect. **Source notes:** Note S3P.

S3-A16. Xue [117]. Reported finding: Individual pit and tensile observations distinguish exposed specimen states. **Interpretation limit:** Five 5 mm wires/state; 0/15 d strength ranges overlap. The 36.03→28.9 kN early force change is a random-pit FE/fit result, not a measured contrast. The source's 60 d corrosion percentage differs between table and text. **Source notes:** S1-56.

S3-A17. Yang profiles [46]. Reported finding: Axial profiles locate local section minima; six low-damage fatigue specimens reach the stopping limit. **Interpretation limit:** One profile wire/group; sampling interval is not accuracy. Extrema 437/797 MPa give $\Delta\sigma = 360$ MPa and $R \approx 0.548$. Six 2×10^6-cycle outcomes are runouts; cycling atmosphere and stress-area convention are unresolved. **Source notes:** S1-12; Table S1E.

S3B. Hydrogen and mechanical response

S3-B01. Nakamura–Suzumura [51]. Reported finding: Low-temperature released hydrogen is below 0.2 ppm in the tested corrosion-level-2 wires. **Interpretation limit:** 1570 MPa-grade finished wire; the 0.7 ppm comparison is protocol-specific. Stressed and unstressed corrosion programmes differ in temperature; hydrogen extraction and fatigue use separate wires. **Source notes:** S1-09; Table S1M.

S3-B02. Cao [52]. Reported finding: Section-related mechanical loss is observed in acidic wet–dry exposure. **Interpretation limit:** No hydrogen-induced or stress-corrosion cracking observed in that protocol; products prevent reliable bulk-hydrogen determination. This is not a universal hydrogen exclusion threshold. **Source notes:** S1-32; Table S1M.

S3-B03. Recio [53]. Reported finding: Alkaline exposure produces hydrogen evolution, diffusible release and ductility loss. **Interpretation limit:** Galvanized prestressing wire/strand at 80% ultimate load. Mercury-displacement outgassing records release after exposure, not retained or fracture-local hydrogen; environment differs from atmospheric bridge cables. **Source notes:** S1-36; Table S1M.

S3-B04. Zhang [8]. Reported finding: Coated and stripped preparations show different desorption signals after thiocyanate exposure. **Interpretation limit:** FIP is a preparation/loading-control case only. Stripping, polishing and rod chemistry differ; condition allocation, aggregation and state-area normalization are unresolved. Separate desorption signals are not integrated contents or specimen-linked fracture evidence; no quantitative coating effect or ranking. **Source notes:** S1-02; Table S1M.

S3-B05. Enos–Scully [54]. Reported finding: Fracture-initiation stress decreases with permeation-derived hydrogen in notched prestressing steel. **Interpretation limit:** Measured supply–fracture response belongs to its notched configuration. The local critical-strain/colony-distance criterion is finite-element-assisted interpretation, not a directly measured coated-wire atmospheric threshold. **Source notes:** S1-06; Table S1M.

S3-B06. Jia [55]. Reported finding: Mechanical response varies with precharge duration and hydrogen supplied during deformation. **Interpretation limit:** Machined 3 mm tensile section; precharge 1 mA/cm²; three tests/condition. Conflicting condition assignment precludes one numerical elongation-loss comparison. TDS plates are separate, and no APT was reported. Imposed charging is not atmospheric entry flux. **Source notes:** S1-07; Table S1M; Note S3H.

S3-B07. Huang [56]. Reported finding: Within-state ductility recovers after desorption; separate specimens retain high-temperature release and local deuterium. **Interpretation limit:** Tensile gauges and processing histories differ; n = 3/condition. TDS/APT/tensile preparations are separate. Undefined source Error field is not SD; retention does not isolate a protective trap effect. Peak-shift, transport-input and APT-scale energy parameters are distinct. **Source notes:** S1-08; Table S1M; Note S3H.

S3-B08. Doshida–Takai [57]. Reported finding: Retained peak 2 hydrogen adds no detected ductility loss versus the thermally matched control. **Interpretation limit:** 1838 MPa pearlite; selective 200 °C/45 min desorption; ~2.6 mass ppm retained. Both-peak 30/70 °C tensile tests continue charging. The result belongs to that population and protocol, not every high-temperature peak. **Source notes:** S1-61; Table S1M.

S3-B09. Elices [60]. Reported finding: Stabilization lowers strength and surface-ferrite residual stress while increasing reported thiocyanate fracture time. **Interpretation limit:** Strength, residual stress and absolute test force co-vary. FIP sample-count narrative and tables disagree; XRD counts are measurements, not independent wires. Measured surface-ferrite stress differs from a mixture-rule macro-stress estimate. **Source notes:** S1-10; Table S1M.

S3-B10. Hirakami [61]. Reported finding: Aging raises strength and charged/uncharged fracture-stress ratios while lowering the approximately 100 °C peak hydrogen. **Interpretation limit:** 5 mm SWRS82B; 10 min at 100/300 °C. Notched tests precharge and continue charging; TDA uses 100 °C/h. The contrast supplies neither a universal hydrogen threshold nor identification of all peak hydrogen as fracture-active. **Source notes:** S1-05; Table S1M.

S3-B11. Tomatsu [62]. Reported finding: Miniature charged specimens show orientation-sensitive fracture stresses and paths. **Interpretation limit:** One air control/orientation; miniature notch and plasma charging. Transverse nominal fracture planes contain the drawing direction; local paths deviate. A stationary longitudinal subcrack is not a common final plane, service contact angle or saddle limit. **Source notes:** S1-23; Table S1M.

S3C. Fatigue, contact and sustained loading

S3-C01. Wang: notched environmental fatigue [81]. Reported finding: Source-listed solution-exposed lives are shorter than air entries; the two NaCl concentrations have reversed ordering. **Interpretation limit:** Prescribed notches; verified stress range, 0.5 Hz, R ≈ 0.44. Per-condition count, aggregation and dispersion are unresolved. The 3.5/0.5% NaCl ordering is descriptive; oxygen was not measured, and no quantitative effect is inferred. **Source notes:** S1-13; Table S2A.

S3-C02. Wang: 1 mm notch/droplet tests [69]. Reported finding: Prepared-notch lives are lower during saline-droplet exposure than in air at the reported settings. **Interpretation limit:** One source-listed specimen at each of four settings per medium; σa denotes stress range in the source. Prepared geometry and local droplets do not establish natural-pit initiation or intact-coating history. **Source notes:** S1-51.

S3-C03. Lan/Bing: exposed-steel-window family [74, 75]. Reported finding: One exposed-window experimental family records corrosion-fatigue failures and air runouts. **Interpretation limit:** Matching 71 tests plus 12 air runouts and linked preparation are treated as one family. A 1.5 mm imposed-potential steel window bypasses intact zinc. The modeled pit-to-crack transition was not directly followed during loading. **Source notes:** S1-50.

S3-C04. Yao: polymer-overcoated/galvanized preparations [116]. Reported finding: The source reports staged corrosion and fatigue observations across two coating categories. **Interpretation limit:** Methods allocate 60 polymer-overcoated and 12 galvanized-only wires but describe 48 fatigue specimens without coating-category allocation. Fatigue values cannot be assigned to the galvanized-only subset. **Source notes:** S1-55.

S3-C05. Ma: crack-history updating [78]. Reported finding: Measured beach-mark histories constrain a fitted crack-growth model. **Interpretation limit:** Physical model fitted to beach-mark histories from 12 wires; Bayesian updating illustrated with a simulated history. Distinguish measured fitted histories from the simulated updating example. **Source notes:** Note S3P.

S3-C06. Deng and Deng: prepared-state comparison [79]. Reported finding: Separate polished and unpolished groups show different pre-exposed fatigue responses. **Interpretation limit:** Separate recovered-wire groups; three wires per polished/unpolished state at each pre-exposure duration. ΔS is defined as range, with reported maximum stress and R consistent with ≈360 MPa. Polishing alters surface geometry and potentially steel section; no paired isolated-pit effect. **Source notes:** S1-21.

S3-C07. Miyachi: new and salt-spray-corroded wires [80]. Reported finding: Nine individual records place corroded-wire lives above new-wire lives at the stated stress range. **Interpretation limit:** Five new and four corroded individual

records do not overlap at $\Delta\sigma$ = 650 MPa. Independent corrosion batches and matched initial pit geometry were unreported. Report the reversal without attributing a beneficial effect to corrosion. **Source notes:** S1-14; Table S2C.

S3-C08. Xue and Shen: pre-corrosion then fatigue [111]. Reported finding: Post-exposure fatigue changes are recorded after acid salt spray. **Interpretation limit:** 7 mm Zn-coated wires received 15–90 days acid salt spray before fatigue. The protocol characterizes downstream pre-corroded states without registering first coating breach. **Source notes:** S1-33.

S3-C09. Cai: Zn–10Al–RE product [112]. Reported finding: A Zn–10Al–RE product shows within-product fatigue changes after pre-exposure. **Interpretation limit:** Three wires per exposure–stress cell after 0–150 days neutral salt spray. Stress terminology prevents numerical pooling; the series supplies within-product post-exposure observations. **Source notes:** S1-34.

S3-C10. Xue and Shen: prefatigue then seawater fatigue [83]. Reported finding: Subsequent seawater-fatigue means vary non-monotonically with prior dry cycling. **Interpretation limit:** Four nonzero prefatigue levels; some higher-precycle groups had longer subsequent mean lives, but the trend was not universal and no zero-prefatigue control was included. This history is not pooled with pre-corrosion or continuously wet tests. **Source notes:** S1-46.

S3-C11. Lan: imposed-potential corrosion fatigue [82]. Reported finding: Life varies non-monotonically across imposed potentials. **Interpretation limit:** Non-monotonic life response across imposed potentials is retained. Dissolution, hydrogen and evolving geometry were not resolved by registered crack histories; no universal potential–life relation is inferred. **Source notes:** S1-43.

S3-C12. Xu: prescribed 100 µm-deep cut [84]. Reported finding: Means decrease from air to humid air and salt spray after a prescribed steel-exposing cut. **Interpretation limit:** Steel deliberately exposed before loading. At $\Delta\sigma$ = 360 MPa, n = 15 in air and 10 per wet condition; plotted SD is calculated from reported COV. At 240 MPa in air, a 2×10^6-cycle runout was omitted from the published mean. **Source notes:** S1-58.

S3-C13. Li: electrochemical endpoint under cycling [85]. Reported finding: Loaded polarization specimens show coating cracks and an earlier potential-transition endpoint than unloaded specimens. **Interpretation limit:** 7 mm wire; nominal 0.5 cm^2 window; 20 mA/cm^2 in 3.5% NaCl near 25 °C. Endpoint is fitted D/E potential-segment intersection, not complete zinc removal or fatigue failure. Condition-level aggregation/dispersion unresolved; conversions $N = ft$ and $q = jt$ are arithmetic. **Source notes:** S1-15; Table S2B.

S3-C14. Liu: salt-spray/pickled starting state [86]. Reported finding: Fracture surfaces show multiple pit-associated origins and propagation regions in exposed steel. **Interpretation limit:** Fatigue followed salt-spray corrosion and pickling. Multiple pit-associated origins and propagation regions describe already exposed steel; the experiment does not start from an intact coating. **Source notes:** Note S3P.

S3-C15. Montalvo: crossed-wire fretting fatigue [87]. Reported finding: An interrupted fretting test detects a crack while zinc remains elsewhere in the contact scar. **Interpretation limit:** 4.85 mm Zn–5%Al/stripped wires; 30°, 1400 N, ambient ungreased contact. Effective displacement corrects elongation and depends on rig stiffness; it is not local slip. Crack coexists with zinc elsewhere in scar, not proven intact coating over the crack origin. **Source notes:** S1-16; Table S1N.

S3-C16. Wang: reciprocating corrosion–wear [88]. Reported finding: Total corrosion–wear volume rises while the distance-normalized wear coefficient falls with displacement. **Interpretation limit:** 100 N, 1 Hz, 30 °C, 10^5 cycles; imposed displacement 100–1000 µm. Total wear volume and load/distance-normalized wear coefficient have different trends. Wear coefficient is not friction coefficient. **Source notes:** Note S3P.

S3-C17. Wang/Chong: contact-scar chemistry [89, 90]. Reported finding: Contact-scar EDS shows local zinc depletion and iron enrichment. **Interpretation limit:** Surface EDS describes local scars, not whole-wire zinc loss. Cycle-stop points are not replicate counts per condition. Contact geometries and slips differ; potentially overlapping reports are not counted as independent confirmation. **Source notes:** S1-53; S1-54.

S3-C18. Liu: imposed-current corrosion–fretting fatigue [91]. Reported finding: Mean fatigue life declines with imposed current in the tested contact protocol. **Interpretation limit:** 5 mm wire, stated 0.88 mm displacement and 120 N; five source-listed lives per current group. Current-dependent mean life describes this accelerated contact protocol, not an atmospheric corrosion-rate or cable-capacity law. **Source notes:** S1-44.

S3-C19. Chehrazi: dry saddle contact [92]. Reported finding: Galvanized dry-contact fatigue lives vary widely within the sampled products. **Interpretation limit:** Bare and galvanized central wires came from different strand products with different contact states. Galvanized lives spanned more than fivefold at one condition; the ordering does not isolate a zinc-coating effect. **Source notes:** S1-37.

S3-C20. Zhang: polarized sustained loading [93]. Reported finding: Fracture time varies non-monotonically with polarization; strong anodic exposure also reduces the remaining section. **Interpretation limit:** Acid-stripped wire; 70%

source-specified maximum force; 20 wt.% NH4SCN, 50 °C. Desorption and fracture specimens had different treatments. Retain the non-monotonic fracture-time response and section measurement without assigning a measured hydrogen population to an individual fracture. **Source notes:** S1-17.

S3D. Model and assembly endpoints

S3-D01. Yang et al. [76]. Reported finding: Six wire-life tests outside calibration check terminal-life predictions. **Interpretation limit:** Solution-exposed-window protocol. Initial coating state and intermediate pit/crack history unreported; the independent check addresses terminal wire life, not cable force or capacity. **Source notes:** S1-52.

S3-D02. An et al. [99]. Reported finding: Two published 69-wire cable tests check selected third- and seventh-break cycles. **Interpretation limit:** Selected break timing is the endpoint; retained-force and residual-capacity predictions were not tested. **Source notes:** S1-42.

S3-D03. Zheng and Wang [77]. Reported finding: The model reproduces wire-life series used in fitting and within-source checks. **Interpretation limit:** Undamaged and corrosion-fatigue fits/checks reuse their respective published source series. Pre-cracked comparator specimens are not equivalent. This is calibration/within-source agreement, not held-out prediction. **Source notes:** Note S3P.

S3-D04. Duan et al.; Guo et al. [113, 114]. Reported finding: Staged models agree with selected calculations under their stated assumptions. **Interpretation limit:** Comparisons against other models do not independently validate the corresponding experimental stage chronology. **Source notes:** S1-38; S1-39.

S3-D05. Lan et al. [101]. Reported finding: An equal-sharing model updates survivor stress after assumed wire breaks. **Interpretation limit:** 300 wires with common stiffness/corrosion and independent initial lives. The arithmetic does not establish spatial resistance, measured force recovery or a cable-life factor. **Source notes:** S1-19; Note S3L.

S3-D06. Li et al. [97]. Reported finding: A local saddle model identifies configuration-dependent contact fatigue at the groove outlet. **Interpretation limit:** Five 5.65 mm wires; specified groove, curvature and imposed equivalent pressures. Calculated interlayer compression is not a measured local pressure field or universal saddle-fatigue limit. **Source notes:** S1-20.

S3-D07. Ding et al. [102]. Reported finding: An idealized pit-position model predicts greater concentration near a restrained anchorage. **Interpretation limit:** Pit dimensions fixed and end rotation prescribed; calculated sensitivity requires matching component strain or failure-location evidence for transfer. **Source notes:** S1-18.

S3-D08. Zhao et al. [103]. Reported finding: Bridge frequency and deflection check the modeled structural response. **Interpretation limit:** Imported Galfan-loss and coating/steel/pit/traffic inputs are distinct from this response check. It does not validate coating chronology or predicted lifetime. **Source notes:** S1-27.

S3-D09. Yuan et al. [98]. Reported finding: Wire tests check predicted yield and ultimate strengths. **Interpretation limit:** The 1,910 learning rows include interpolation and 2,121 cases are numerical grid points. Internal row splitting does not establish independent-batch or cable-force/capacity validation. **Source notes:** S1-60.

S3-D10. Xue and Shen [100]. Reported finding: One 121-wire cable supplies first-through-ninth break histories. **Interpretation limit:** Boundary stiffness is adjusted to stress measured in that same cable. The test stops before capacity failure; initial coating state and solution composition are unspecified. This is same-test reproduction. **Source notes:** S1-28.

S3-D11. He [107]. Reported finding: Laboratory bundles show depth-dependent electromagnetic detection of retired-wire corrosion. **Interpretation limit:** Four retired 7 mm wires; first-layer states distinguished, second-layer presence detectable, third-layer levels unresolved. Detection is not a mechanical-force endpoint. **Source notes:** S1-29.

Supplementary Note S3H. Desorption-energy quantities and specimen scale

Huang et al. [56] report 47 kJ/mol from Kissinger peak shifts in their 100, 150 and 200 °C/h heating series to 500 °C, use 80 kJ/mol as an Oriani/McNabb–Foster bulk-transport/trapping input, and require an effective APT-tip detrapping energy above 105 kJ/mol to reproduce retention. The 80 kJ/mol input predicted loss from tips within about 10 s, whereas deuterium remained after 2 h. These are a peak-shift estimate, a model input and a scale-specific effective parameter, not three measurements of one trap-binding energy. Jia's separately prepared plate specimens gave fitted peak values of 26.98 and 53.09 kJ/mol [55], with several candidate origins for the higher-temperature feature. Neither fitted peak energy alone identifies mechanically active hydrogen.

Supplementary Note S3P. Additional comparison identities

Recovered-wire fatigue. Li et al. [45] compared pitted and uniformly corroded recovered-wire specimens under matched study loads. The reported shorter pitted-specimen lives concern those downstream material states; the comparison does not locate first coating breach or provide a cross-study pooled effect.

Model calibration and history. Zheng and Wang [77] fitted and checked undamaged-wire parameters against the same published series. Their corrosion-fatigue fit and checks used another shared published source, including non-equivalent pre-cracked specimens; these are calibration and within-source checks. Ma et al. [78] fitted a physical model to beach-mark histories from 12 wires and illustrated Bayesian updating with a simulated history. Measured fitted histories, simulated updating and an independent endpoint test are different forms of evidence.

Prepared-steel and contact responses. Liu et al. [86] combined electrochemical measurements and fatigue tests after salt-spray corrosion and pickling; multiple pit-associated origins and propagation regions describe already exposed steel. Wang et al. [88] tested reciprocating galvanized-wire contact in 3.5 wt.% NaCl and deionized water at 100 N, 1 Hz and 30 °C for 10^5 cycles. Increasing displacement from 100 to 1000 µm increased total corrosion–wear volume but reduced the coefficient normalized by load and sliding distance. That wear coefficient is not a friction coefficient; its decline does not mean less total material loss.

Supplementary Note S3L. Illustrative equal-load-sharing arithmetic

The following calculation describes the assumptions of the published model; it is not a measured cable response or a validated service-life example.

For parallel-wire stays, Lan et al. represented fatigue as successive wire failures with load redistribution [101]. Their model assumed 300 wires, a common corrosion level and stiffness, equal load sharing, and independent initial fatigue lives. Under its equal-sharing equation, a nominal stress range of 335 MPa on each intact wire becomes 300 × 335/(300 − 15) = 352.6 MPa on each survivor after 15 assumed breaks, a 5.3% increase. This is conditional model arithmetic, not a measured cable stress or life. A cable with spatially correlated wetting, corrosion or initial defects need not satisfy those assumptions. Applying a wire fatigue distribution therefore requires corresponding distributions and correlations of resistance, stiffness, corrosion and load, followed by checks against break locations, sequence and measured redistribution in an assembly. Under the same equal-sharing assumption, 14, 15 and 16 broken wires give 351.4, 352.6 and 353.9 MPa per surviving wire. This ±1-break calculation is input sensitivity, not a confidence interval. With unequal stiffness or spatially correlated corrosion, joint distributions of remaining area, pit damage, stiffness, initial fatigue resistance and restraint must be propagated to the predicted break order, redistribution or retained force and checked against the matching assembly endpoint. Those input distributions are not reported for this example.
